# Supplementary material for: Uncomputably complex renormalisation group flows
Source: Nat Commun. 2022 Dec 9;13:7618. doi: 10.1038/s41467-022-35179-4 (PMC9734166; doi:10.1038/s41467-022-35179-4)
Supplement: Supplementary file 1 — Supplementary Information [file 41467_2022_35179_MOESM1_ESM.pdf]

Uncomputably Complex Renormalisation Group Flows

Supplementary Information

James D. Watson, Emilio Onorati, and Toby S. Cubitt

Department of Computer Science, University College London, UK

A Preliminaries

A.1 Notation

Throughout we will denote the $L \times W$ square lattice by $\Lambda(L \times W)$ and if $L = W$ we will write $\Lambda(L)$. For points $i, j \in \Lambda(L \times W)$, we will sometimes use $\langle i, j \rangle$ to indicate that they are nearest neighbours. For a Hilbert space $\mathcal{H}$, $\mathcal{B}(\mathcal{H})$ denotes the set of bounded linear operators on $\mathcal{H}$. $\lambda_0(A)$ is the minimum eigenvalue of an operator $A \in \mathcal{B}(\mathcal{H})$, and more generally $\lambda_k(A)$ represents the $(k+1)^{th}$ smallest eigenvalue. Furthermore, we define the spectral gap of an operator A as $\Delta(A) = \lambda_1(A) - \lambda_0(A)$.

We consider local interaction terms $h^{row}, h^{col} \in \mathcal{B}(\mathbb{C}^d \otimes \mathbb{C}^d)$ and $h^{(1)} \in \mathcal{B}(\mathbb{C}^d)$ which define a translationally invariant Hamiltonian on an $L \times L$ lattice,

$$H^{\Lambda(L)} = \sum_{j=1}^{L} \sum_{i=1}^{L-1} h^{row}_{i,i+1} + \sum_{i=1}^{L} \sum_{j=1}^{L-1} h^{col}_{j,j+1} + \sum_{i,j=1}^{L} h^{(1)}_{i,j}, \tag{A.1}$$

where the sums over i and j run over rows and columns respectively. It will occasionally be useful to distinguish h^{row} acting on given row j. When this is important, we write $h^{row}_{i,i+1}(j)$ to denote the interaction between columns i and $i+1$ in the jth row. Similarly $h^{col}_{j,j+1}(i)$ denotes the interaction between rows j and $j+1$ in the ith column.

We denote our renormalisation group map — which we will explicitly construct in the course of this work — by $\mathcal{R}$, and the k-fold consecutive applications of this map by $\mathcal{R}^{(k)}$. Renormalised quantities and operators will be

written with the prefix R, and with $R^{(k)}$ denoting the k-times renormalised case: for example, the renormalised Hamiltonians terms are $R(h^{row})^{i,i+1}$ and $R(h^{col})^{j,j+1}$, and the local terms after k-fold iterations $R^{(k)}(h^{row})^{i,i+1}$ and $R^{(k)}(h^{col})^{j,j+1}$. We then denote the Hamiltonian defined over the lattice by the renormalised interactions as $R(H)^{\Lambda(L)}$, and for the k-times iteration as $R^{(k)}(H)^{\Lambda(L)}$. We remark that in general $\mathcal{R}(h^{row}_{i,i+1}) \neq R(h^{row})^{i,i+1}$, and analogously for the other terms.
In the same vein, if the initial local Hilbert space is $\mathcal{H}$, then the local Hilbert space after k iterations of the RG map is indicated by $R^{(k)}(\mathcal{H})$. Throughout, we will denote a canonical set of local basis states by $\mathfrak{B}$, and after the renormalisation mapping has been applied k times it becomes $\mathfrak{B}^{(k)}$, so that $R^{(k)}(\mathcal{H}) = \operatorname{span}\{|x\rangle \in \mathfrak{B}^{(k)}\}$.

Following [1], we adopt the following precise definitions of gapped and gapless:

Definition 1 (Gapped)**.** *We say that $H^{\Lambda(L)}$ of Hamiltonians is gapped if there is a constant $\gamma > 0$ and a system size $L_0 \in \mathbb{N}$ such that for all $L > L_0$, $\lambda_0(H^{\Lambda(L)})$ is non-degenerate and $\Delta(H^{\Lambda(L)}) \geq \gamma$. In this case, we say that* the spectral gap is at least γ.

Definition 2 (Gapless)**.** *We say that $H^{\Lambda(L)}$ is gapless if there is a constant $c > 0$ such that for all $\epsilon > 0$ there is an $L_0 \in \mathbb{N}$ so that for all $L > L_0$ any point in $[\lambda_0(H^{\Lambda(L)}), \lambda_0(H^{\Lambda(L)}) + c]$ is within distance ϵ from $\operatorname{spec} H^{\Lambda(L)}$.*

We note that these definitions of gapped and gapless do not characterise all Hamiltonians; there are Hamiltonian which fit into neither definition, such as systems with closing gap or degenerate ground states. However, [1] showed that the particular Hamiltonians they construct always fall into one of these clear-cut cases, allowing sharp spectral gap undecidability results to be proven.

Finally, in this work we will be interested in phase transitions. In general, across the boundary of two phases there is a non-analytic change in some properties (usually a local observable). Thus phase transitions are often characterised in terms of an *order parameter*. For the purposes of this work, an order parameter is a scalar quantity which changes non-analytically across the phase boundary and hence defines a phase transition. We elaborate on the order parameter used in this work in Subsection A.3.3.

A.2 Hilbert Space Structure and Local Interaction Terms

To build the Hamiltonian with desired undecidable spectral energy properties, we break down the Hilbert space into two components, together with an auxiliary zero-energy state $|0\rangle$, namely,

$$\mathcal{H} = \mathcal{H}_u \otimes \mathcal{H}_d \oplus |0\rangle \,. \tag{A.2}$$

Here $\mathcal{H}_d$ is the Hilbert space associated with the dense spectrum Hamiltonian H_d, and $\mathcal{H}_u$ is the Hilbert space associated with the Hamiltonian with undecidable ground state energy H_u. Let $h_d^{(i,j)} \in \mathcal{B}(\mathbb{C}^2 \otimes \mathbb{C}^2)$ be the local interactions of the 1D critical XY model. Let H_d be the Hamiltonian composed of XY interactions along the rows of the lattice; this has a dense spectrum in the thermodynamic limit [2].

The local interactions along the edges and on the sites of the lattice act on this local Hilbert space as

$$h(\varphi)^{(i,j)} = |0\rangle \langle 0|^{(i)} \otimes \Pi_{ud}^{(j)} + \Pi_{ud}^{(i)} \otimes |0\rangle \langle 0|^{(j)} \tag{A.3}$$

$$+ h_u^{(i,j)}(\varphi) \otimes \mathbb{1}_d^{(i,j)} + \mathbb{1}_u^{(i,j)} \otimes h_d^{(i,j)} \tag{A.4}$$

$$h(\varphi)^{(1)} = -(1+\alpha_2)\Pi_{ud}, \tag{A.5}$$

where $\Pi_{ud}^{(j)} := (\mathbb{1} - |0\rangle \langle 0|)^{(j)}$ is a projector onto $\mathcal{H}_u \otimes \mathcal{H}_d$, and $\alpha_2 = \alpha_2(|\varphi|)$ is a constant depending only on $|\varphi|$. For completeness we note as per the Hamiltonian decomposition in eq. (1) of the main manuscript, $H_{guard} = \sum_{\langle i,j \rangle} |0\rangle \langle 0|^{(i)} \otimes \Pi_{ud}^{(j)} + \Pi_{ud}^{(i)} \otimes |0\rangle \langle 0|^{(j)}$. Crucially, this structure of the overall lattice Hamiltonian implies that its spectrum is

$$\operatorname{spec} H(\varphi) = \{0\} \cup \{\operatorname{spec}(H_u(\varphi)) + \operatorname{spec}(H_d)\} \cup S, \tag{A.6}$$

for a set S with all elements > 1. This means that if $\lambda_0(H_u^{\Lambda(L)}) \to -\infty$ then the overall Hamiltonian has a dense spectrum, while if $\lambda_0(H_u^{\Lambda(L)}) \to +\infty$ the overall Hamiltonian has a spectral gap > 1.

Let H_q be the modified version of the *Gottesman-Irani Hamiltonian* (more details in Subsection D.1) with associated local Hilbert space $\mathcal{H}_q$. Then, we further divide the local Hilbert space $\mathcal{H}_u$ into

$$\mathcal{H}_u = \mathcal{H}_c \otimes (\mathcal{H}_q \oplus |e\rangle), \tag{A.7}$$

where $|e\rangle$ is another zero-energy filler state[1]. In this framework the Hamiltonian $H_u(\varphi)$ has a ground state composed by two parts very different in nature: a classical "tiling layer" $|T\rangle_c \in \mathcal{H}_c^{\otimes\Lambda(L\times L)}$ and a highly entangled "quantum layer" $|\psi\rangle_{eq} \in (\mathcal{H}_q \oplus |e\rangle)^{\otimes\Lambda(L\times L)}$. Thus $\mathcal{H}_c$ is the Hilbert space corresponding to the classical tiling layer and $\mathcal{H}_q \oplus |e\rangle$ is the "quantum" layer.
More specifically, let $h_u^{(i,j)} \in \mathcal{B}(\mathbb{C}^d \otimes \mathbb{C}^d)$ be the local terms of the Hamiltonian H_u with the form

$$h_u = h_T^{(i,i+1)} \otimes \mathbb{1}_{eq}^{(i)} \otimes \mathbb{1}_{eq}^{(i+1)} + \mathbb{1}_c^{(i)} \otimes \mathbb{1}_c^{(i+1)} \otimes h_q^{(i,i+1)} + \text{"coupling terms"},$$

designed so that $H_u(\varphi) = \sum h_u(\varphi)$ has a ground state energy which depends on whether a universal Turing Machine (UTM) halts when given on input φ supplied in binary. In particular, on a lattice of size $L \times L$, the ground state energy is

$$\lambda_0(H_u^{\Lambda(L)}) = \begin{cases} -\Omega(L) & \text{if UTM does not halt on input } \varphi, \\ +\Omega(L^2) & \text{if UTM does halt on input } \varphi. \end{cases} \tag{A.8}$$

In the $\lambda_0(H_u^{\Lambda(L)}(\varphi)) = +\Omega(L^2)$ case, the ground state of the entire Hamiltonian is $|0\rangle^{\Lambda}$. In the $\lambda_0(H_u^{\Lambda(L)}(\varphi)) = -\Omega(L)$ case, the overall ground state is $|\psi_u\rangle \otimes |\psi_d\rangle$ where $|\psi_u\rangle$ and $|\psi_d\rangle$ are the ground states of $H_u(\varphi)$ and $H_d = \sum_{i\in\Lambda} h_d^{i,i+1}$ respectively. But since the halting problem is undecidable, determining which of the two ground state energies of $H_u(\varphi)$ occurs is undecidable.

The fundamental ingredient underlying this whole construction is the "QTM-to-Hamiltonian" mapping, originally developed by Gottesman and Irani [3] (but based on a construction by Kitaev [4]), which takes a QTM and maps its evolution to the ground state of a 1D, translationally invariant, nearest neighbour Hamiltonian. A QTM can be thought of as a classical TM, but where the TM head and tape configuration can be in a superposition of states. The successive steps of the QTM's operation and tape configuration at time t are then described by a transition unitary, U_t, such that the state is updated as $|\psi_i\rangle \mapsto U_t |\psi_i\rangle$. The QTM-to-Hamiltonian mapping takes

[1]We note that $|e\rangle$ is a change in notation from [1], which used $|0\rangle$ to denote this state. We change the notation to prevent confusion with the other $|0\rangle \in \mathcal{H}_3$ state

a given quantum QTM and creates a corresponding Hamiltonian which has a ground state which encodes the QTM's evolution. This quantum state is called a *history state*. Let $|\psi_t\rangle$ be the state describing the configuration of the QTM after t steps. Then the history state takes the general form

$$|\Psi_{hist}\rangle = \frac{1}{\sqrt{T}} \sum_{t=1}^{T} |\psi_t\rangle |t\rangle \,, \tag{A.9}$$

where $|t\rangle$ is a state labelling which step of the computation $|\psi_t\rangle$ corresponds to. It is then possible to add a local projector term to the Hamiltonian which gives an additional energy penalty to certain outcomes of the encoded computation. In particular, [1] penalise the halting state, so that if the encoded QTM halts at some point, the Hamiltonian defined by h_q picks up an additional energy contribution. As a result, the energy of the ground state differs depending on whether or not the QTM halts within time T.

A.3 Renormalisation Group Map

The notion of what exactly constitutes a renormalisation group scheme is somewhat imprecise, and there is no universally agreed upon definition in the literature. We therefore start from a minimal set of conditions that we would like a mapping on Hamiltonians to satisfy, if it is to be considered a reasonable RG map. The RG scheme we define for the Hamiltonian from [1] will satisfy all these conditions as well as additional desirable properties.

Definition 3 (Renormalisation Group (RG) Map)**.** *Let $\{h_i\}_i$ be an arbitrary set of r-local interactions $h_i \in \mathcal{B}((\mathbb{C}^d)^{\otimes r})$, for $r, d \in \mathbb{N}$. A renormalisation group (RG) map*

$$\mathcal{R}(\{h_i\}) = \{h_i'\} \tag{A.10}$$

is a mapping from one set of r-local interactions to a new set of r'-local interactions $h_i' \in \mathcal{B}((\mathbb{C}^{d'})^{\otimes r'})$, with $r', d' \in \mathbb{N}$, satisfying the following properties:

1. *$\mathcal{R}(\{h_i\})$ is a computable map.*
2. *Let H and $R^{(k)}(H)$ be the Hamiltonian defined by the original local terms and the k-times renormalised local terms respectively. If H is gapless, then $R^{(k)}(H)$ is gapless, as per Definition 2. If H is gapped, then*

$R^{(k)}(H)$ is gapped, as per Definition 1.

3. *If the order parameter for the system has a non-analyticity between two phases of H, then there is a renormalised order parameter which also has a non-analyticity between the two phases for $R^{(k)}(H)$.*

4. *If the initial local Hamiltonian terms can decomposed into as*

$$h_i = \sum_j \alpha_j O_j, \tag{A.11}$$

for some operator $\{O_j\}_j$, then k-times renormalised local Hamiltonian terms are of the form

$$R^{(k)}(h)_i = \sum_j \alpha_j^{(k)} R^{(k)}(O)_j, \tag{A.12}$$

where $\alpha_i^{(k)} = f(\{\alpha_i^{(k-1)}\}_i)$ for some function f.

The motivation for points 2 and 3 of Definition 3 is that we want to preserve the quantum phase diagram of the system. Point 3 of Definition 3 requires that if we start in phase A, the system should remain in phase A under the RG flow: a key property of any RG scheme. Furthermore, any indicators of a phase change still occur (e.g. non-analyticity of the order parameter). Point 4 asks that the “form” of the Hamiltonian is preserved.
We note that many well-known renormalisation group schemes fit the criteria given in Definition 3 when applied to the appropriate Hamiltonians. On the other hand, a given RG scheme may satisfy the conditions for the family of Hamiltonians it was designed for, but will not necessarily satisfy all the desired conditions when applied to an arbitrary Hamiltonian.

Hamiltonians under RG flows have *fixed points* which are invariant with respect to the action of the RG procedure. If $H^* = \sum h^*$ is the fixed point of some Hamiltonian H converging under the RG flow, then the local terms of H can be rewritten with respect to their deviation from the fix point as

$$h = h^* + \sum_i \beta_i O_i$$

and after renormalisation

$$R^{(k)}(h) = h^* + \sum_i \beta_i^{(k)} O_i',$$

where if $\beta_i^{(k)} \to 0$ as $k \to \infty$ then O_i is said to be an *irrelevant operator*; if $\beta_i^{(k)} \to \infty$, then O_i is a *relevant operator*; and if $\beta_i^{(k)} \to c$ for a constant c, then O_i a *marginal operator* [5].

A.3.1 Comparison to Well Known RG Schemes

Classical 1D Ising Model
A particularly famous RG scheme which satisfies Definition 3 is the *decimation* scheme for the classical 1D Ising model [5]. The Hamiltonian is given as

$$H = J \sum_i \sigma_i \sigma_{i+1} + h \sum_i \sigma_i$$

Here the ground states are trivially either $\sigma_i = 1$ or -1 for all sites i. Under the decimation RG procedure, half the spins are removed by "averaging out" the others. The order parameter for the phase is the magnetisation: $M = \frac{1}{N} \sum_{i=1}^{N} \sigma_i$ and it can be seen to undergo a non-analytic change between phases. This is true even after renormalisation, thus satisfying point 3 of Definition 3. The decimation mapping further gives a transformation of the form

$$\mathcal{R} : J \sum_i \sigma_i \sigma_{i+1} + h \sum_i \sigma_i + CN \to J' \sum_i \sigma_i \sigma_{i+1} + h' \sum_i \sigma_i + C'N,$$

thus satisfying condition 4.

MERA
A more recent and widely studied RG flow scheme in the quantum information literature is the multi scale entanglement renormalisation ansatz (MERA) developed in [6]. This is implemented by iteratively applying isometries to the local terms to produce new local Hamiltonian terms and density matrices. This (approximately) preserves expectation values and hence can often be made to satisfy 3. Whether conditions 2 and 4 are satisfied is

dependent on the Hamiltonian and isometries in question.

A.3.2 The Block Spin Renormalisation Group Map

We base our RG map on a blocking technique widely used in the literature to study spin systems, often called the Block Spin Renormalisation Group (BRG)[2] [7, 8, 9, 10]. Modifications and variations of this RG scheme have also been extensively studied [11, 12].

The BRG is among the simplest RG schemes. The procedure works by grouping nearby spins together in a block, and then determining the associated energy levels and eigenstates of this block by diagonalisation. Having done this, high energy (or otherwise unwanted) states are removed resulting in a new Hamiltonian.

As an explicit example, we review the RG process in [8] for the 1D isotropic X-Y model defined below as:

$$H = -\frac{J}{2}\sum_{i=1}^{N-1}(X_iX_{i+1} + Y_iY_{i+1}) + B\sum_{i=1}^{N} Z_i.$$

We then group terms into blocks of 2:

$$\begin{aligned} H &= -\frac{J}{2}\sum_{i\ odd}^{N-1}(X_iX_{i+1} + Y_iY_{i+1}) - \frac{J}{2}\sum_{i\ even}^{N-1}(X_iX_{i+1} + Y_iY_{i+1}) + B\sum_{i=1}^{N} Z_i \\ &= -\frac{J}{2}\sum_{i\ odd}^{N-1}(X_iX_{i+1} + Y_iY_{i+1}) + \sum_{i\ even}^{N-1} h_i \end{aligned}$$

where $h_i = -\frac{J}{2}(X_iX_{i+1} + Y_iY_{i+1}) + BZ_i + BZ_{i+1}$ now contains all terms acting within the two site blocks. Diagonalising this gives 4 states with energies $\{E_0^{(1)}, E_1^{(1)}, E_2^{(1)}, E_3^{(1)}\}$ in ascending order. We truncate the states associated with the two higher energies, and keep the lowest two which we label as $|0\rangle^{(1)}, |0\rangle^{(1)}$ with energies $E_0^{(1)}, E_1^{(1)}$ respectively. We now replace this operate with a new operator, acting on a single block-spin site with the form

$$\frac{(E_0^{(1)} - E_1^{(1)})}{2} Z_i^{(1)} + \frac{(E_0^{(1)} + E_1^{(1)})}{2} \mathbb{1}^{(1)}.$$

[2]This is also sometimes called the "quantum renormalisation group". We will not use this name to avoid potential confusion.

The between-block interaction now need to be determined: to replicate this we use $X = \xi^{(1)}X^{(1)}$, where $\xi^{(1)}$ can be determined by looking at the matrix elements under the new renormalised block basis: i.e. $\langle 0|^{(1)} X |1\rangle^{(1)} = \xi^{(1)} \langle 0|^{(1)} X^{(1)} |1\rangle^{(1)}$. The new two 2-local terms acting on the block spins are then:

$$h^{(1)}_{i,i+1} = -\frac{J^{(1)}}{2}\sum_{i\ odd}^{N/2-1}(X^{(1)}_i X^{(1)}_{i+1} + Y^{(1)}_i Y^{(1)}_{i+1}),$$

where $J^{(1)} = \xi^{(1)2}J$. By introducing an extra depending on the identity, we find a renormalised Hamiltonian:

$$H^{(1)} = -\frac{J^{(1)}}{2}\sum_{i\ odd}^{N/2-1}(X^{(1)}_i X^{(1)}_{i+1} + Y^{(1)}_i Y^{(1)}_{i+1}) + B^{(1)}\sum_{i=1}^{N/2} Z^{(1)}_i + C^{(1)}\sum_{i=1}^{N/2}\mathbb{1}^{(1)}_i,$$

where $C^{(1)} = (E^{(1)}_0 + E^{(1)}_1)/2$. After n iterations of the RG mapping, we have a Hamiltonian

$$H^{(n)} = -\frac{J^{(1)}}{2}\sum_{i\ odd}^{N/2^n-1}(X^{(n)}_i X^{(n)}_{i+1} + Y^{(n)}_i Y^{(n)}_{i+1}) + B^{(n)}\sum_{i=1}^{N/2^n} Z^{(n)}_i + C^{(n)}\sum_{i=1}^{N/2^n}\mathbb{1}^{(n)}_i,$$

where the constants are defined by the same procedure: $J^{(n)} = \xi^{(n)2}J^{(n-1)}$, $B^{(n)} = B^{(n-1)} + (E^{(n)}_0 - E^{(n)}_1)/2$, $C^{(n)} = C^{(n-1)} + (E^{(n)}_0 + E^{(n)}_1)/2$.

Alternative Formulation: The BRG mapping can be reformulated in terms of a series of isometries (or unitaries followed by subspace restrictions). Indeed, we will formulate our RG construction in terms of sets of isometries. Given the local terms of some Hamiltonian, $h_{i,i+1} \in \mathcal{B}(\mathbb{C}^d \otimes \mathbb{C}^d)$, we will consider renormalisation mappings of the form

$$\mathcal{R} : h_{i,i+1} \to V^\dagger h_{i,i+1} V \tag{A.13}$$

where $V : \mathbb{C}^d \to \mathbb{C}^{d'}$ is an isometry which will take a states in the initial set of basis states to a restricted new set of renormalised basis states.

Equivalently we can formulate this in terms of a unitary U and a sub-

space Γ, as:

$$\mathcal{R} : h_{i,i+1} \to U^\dagger h_{i,i+1} U|_\Gamma . \tag{A.14}$$

The unitary U maps the original basis states to the new set (this transformation is what we call *blocking*). This is followed by a restriction to the subspace Γ which is the "low-energy" subspace: that is, all basis states which locally pick up too much energy are removed. This subspace restriction is called *truncating*. In our particular variation of the BRG, the truncation step is not done entirely based on energy truncation, but also on overlap with a particular state.

A.3.3 Order Parameters

Back to claim 3 of Definition 3, we now discuss order parameters in more detail. As noted in [13], the two phases[3] of the Hamiltonian (which we label A and B for convenience) can be distinguished by an order parameter $\langle O_{A/B} \rangle$, where $O_{A/B}$ defined as:

$$O_{A/B} = \frac{1}{|\Lambda|} \sum_{i \in \Lambda} |0\rangle\langle 0|^{(i)} . \tag{A.15}$$

In particular, upon moving from one phase to another, the expectation value of the order parameter is generally undergoing a non-analytic change. In the case $\lambda_0(H_u^{\Lambda(L)}(\varphi)) = +\Omega(L^2)$ the ground state of the entire Hamiltonian is then $|0\rangle^\Lambda$ and hence $\langle O_{A/B} \rangle = 1$, and otherwise $\langle O_{A/B} \rangle = 0$. This is true even if we restrict $O_{A/B}$ to subsections of the lattice, hence $O_{A/B}$ is a local order parameter (as opposed to the global order parameters required to distinguish topological phases) undergoing a non-analytic change between phases, which itself demonstrates a phase transition. More generally for a ball $B(r)$ of radius r, and for a state $|\nu\rangle \in \mathcal{H}^{\otimes \Lambda}$ we can define a local observable:

$$O_{A/B}(r) = \frac{1}{|B(r)|} \sum_{i \in B(r)} |\nu\rangle\langle \nu|^{(i)} , \tag{A.16}$$

which acts as a local order parameter. For this work $|\nu\rangle = |0\rangle$.

[3]Phase in this context refers to the state of matter, not a quantum mechanical phase factor (of the form $e^{i\theta}$).

A.4 Spectral Gap Undecidability Construction

Constructing a mathematically rigorous RG flow for the undecidable Hamiltonian exhibited in [1, 14] presents particular challenges, since its properties are uncomputable. Nonetheless, we are able to achieve this by carefully analysing the local structure and properties of this Hamiltonian, depending on one external parameter, which is gapped iff a universal Turing Machine halts on an input related to the Hamiltonian parameter. The spectral gap problem for this Hamiltonian is therefore equivalent to the Halting Problem, hence undecidable. Here is the explicit definition:

Definition 4 (Main Theorem 3 of [1]). *For any given universal Turing Machine (UTM), we can construct explicitly a dimension d, $d^2 \times d^2$ matrices A, A', B, C, D, D', Π and a rational number β which can be as small as desired, with the following properties:*

1. *A is diagonal with entries in $\mathbb{Z}$.*
2. *A' is Hermitian with entries in $\mathbb{Z} + \frac{1}{\sqrt{2}}\mathbb{Z}$,*
3. *B, C have integer entries,*
4. *D is diagonal with entries in $\mathbb{Z}$,*
5. *D' is Hermitian with entries in $\mathbb{Z}$.*
6. *Π is a diagonal projector.*

For each natural number n, define:

$$\begin{aligned}
h_1(n) &= \alpha(n)\Pi, \\
h_{col}(n) &= D + \beta D', \qquad \textit{independent of } n \\
h_{row}(n) &= A + \beta\left(A' + e^{i\pi\varphi}B + e^{-i\pi\varphi}B^\dagger + e^{i\pi 2^{-|\varphi|}}C + e^{-i\pi 2^{-|\varphi|}}C^\dagger\right),
\end{aligned}$$

where $\alpha(n) \leq \beta$ is an algebraic number computable from n and $|\varphi|$ denotes the length of the binary representation of φ. Then:

1. *The local interaction strength is bounded by 1, i.e.* $\max(\|h_1(n)\|, \|h_{row}(n)\|, \|h_{col}(n)\|) \leq 1$.
2. *If UTM halts on input n, then the associated family of Hamiltonians $\{H^{\Lambda(L)}(n)\}$ is gapped with gap $\gamma \geq 1$.*

3. *If UTM does not halt on input n, then the associated family of Hamiltonians $\{H^{\Lambda(L)}(n)\}$ is gapless.*

B Overview of the Proof

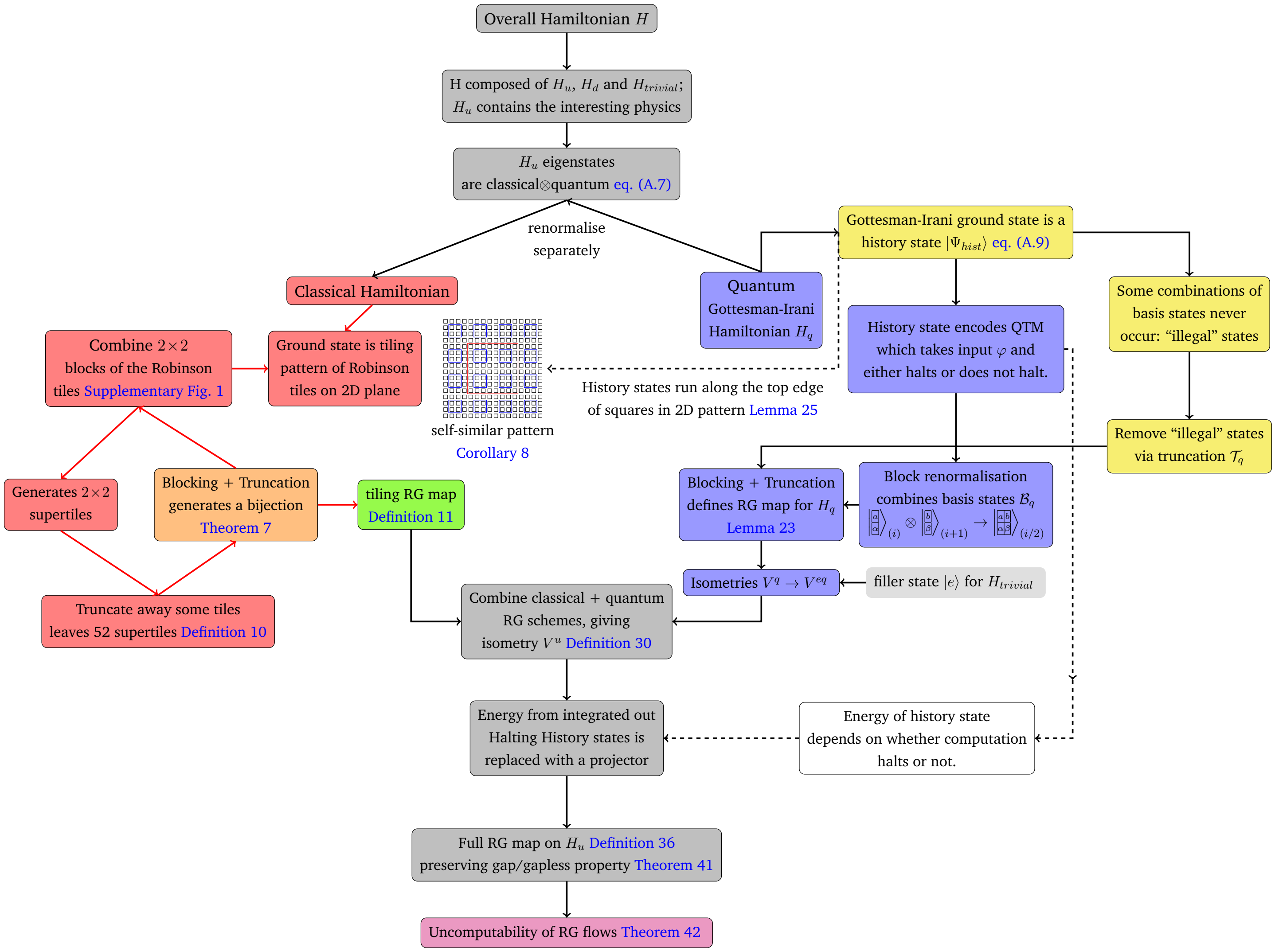

The renormalisation group scheme we will construct is a variant of the BRG scheme, where we block 2×2 groups of spins to a single "super-spin" which preserves the relevant properties of the original system. To simplify the procedure, our strategy is to divide it into separate tasks, which we can then assemble together for our final result. In other words, we will first renormalise the different parts $H_u, H_d, |0\rangle$ of the Hamiltonian separately,

then combine these RG maps into the complete scheme.

As shown in Lemma 26, the eigenstates of $H_u^{\Lambda(L)} = \sum_{\langle i,j \rangle} h_u^{(i,j)}$ have a tensor product form $|T\rangle_c \otimes |\psi\rangle_{eq}$ for $|T\rangle_c \in \mathcal{H}_c^{\otimes\Lambda(L)}$ and $|\psi\rangle_{eq} \in \mathcal{H}_{eq}^{\otimes\Lambda(L)}$. This key property allows us to renormalise again separately the (classical) tiling Hamiltonian and the (quantum) Gottesman-Irani Hamiltonian.

Renormalising the Tiling Hamiltonian The set of *Robinson tiles* (explained in detail in Subsection C.1) can be mapped to a 2D, translationally invariant, nearest neighbour, classical Hamiltonian by connecting each tile type to a state in the local Hilbert space and introducing local interactions that apply an energy penalty to neighbouring pairs which do not satisfy the tiling rules. That is, the local terms are defined as $(h_T)_{i,i+1} := \sum_{(t_\alpha,t_\beta)\notin A} |t_\alpha, t_\beta\rangle\langle t_\alpha, t_\beta|_{i,i+1}$ where A is the set of allowed neighbouring tiles.

Then, the ground state of the classical part of the 2D lattice, $|T\rangle_c$, corresponds to a specific Robinson tiling pattern as shown in Supplementary Fig. 3, where smaller squares are nested within larger ones. Any other configuration must violate a tiling rule and thus receives an energy penalty. Notably, this pattern is self-similar under the BRG construction we define in Definition 6; in simple words, "zooming out" the tiling image will result in the same pattern (Corollary 8). Crucially, this means that the density of the Gottesman-Irani ground states running over the top edges of the squares appearing in the pattern will be the same at every RG iteration.

Our blocking procedure takes a set of 2×2 Robinson tiles, which we call *supertiles*, and following the adjacency rules derived according to the markings of the single tiles, it maps these supertiles back onto Robinson tiles. This induces a bijection between a subset of 56 supertiles and the Robinson tiles set (Theorem 7). We also note that not all supertiles assembled following adjacency rules find a corresponding Robinson tiles under the BRG scheme: these supertiles, however, do not appear in the pattern of Supplementary Fig. 3 and hence must be discarded. We do so by constructing an explicit isometry acting on local terms of the Hamiltonian interaction to filter out those supertiles not appearing in the pattern (Definition 10); we then use the isometry to formalise the tiling Hamiltonian renormalisation (Definition 11) under which we show that the local Hamiltonian terms are mapped back onto themselves (Lemma 12), such that RG procedure preserves both the ground state energy and the tiling pattern (Theorem 13).

The full analysis of the tiling Hamiltonian renormalisation is contained in Section C.

Renormalising the Gottesman-Irani Hamiltonian The Gottesman-Irani Hamiltonian H_q is a 1D Hamiltonian which serves as a QTM-to-Hamiltonian map [3]. In the Hamiltonian from Cubitt, Pérez-García and Wolf [1, 14], ground states of Gottesman-Irani Hamiltonians appear along the top edge of the Robinson tiles. We initially focus on developing an RG mapping for the 1D Hamiltonian, before then considering it in 2D.

We design our RG scheme so that the energy of the Gottesman-Irani ground state attached to a square in the tiling pattern remains the same, even when the square size is halved under the RG mapping. Our strategy is to divide the RG operation into two parts: a blocking operation $\mathcal{B}_q$ and a truncation operation $\mathcal{T}_q$.

For every pair of basis states on neighbouring spins with local dimensions d, $\mathcal{B}_q$ maps them onto a single new renormalised spin of dimensions d^2 (Definition 20). The result is a new combined spin $\mathbb{C}^{d^2}$ and a new Hamiltonian induced over these spins.

The truncation operation $\mathcal{T}_q$ (Definition 21) takes these renormalised states and removes any that are guaranteed to not be part of the overall ground state. As with the BRG, we consider the new 1-local terms an consider the energy of the associated states. The local states which pick up an energy penalty in the new Hamiltonian are removed[4]. This generates a renormalised Hamiltonian acting on the new combined spins, each of which as a local Hilbert space dimension bounded as $< d^2$ (but still $> d$). This blocking procedure will preserve whether the Hamiltonian has a zero energy ground state or a ground state with energy > 0.

The renormalisation procedure is implemented by a series of isometries (Lemma 22) acting on the original Gottesman-Irani terms. This is then a QTM-to-Hamiltonian mapping preserving the overall form of H_q and its ground state (Lemma 23). We refer the reader to Section D, and in particular Lemma 23, for full details.

Renormalising H_u We now want to renormalise H_u. We might naïvely try to straightforwardly combine the classical isometry transformation, V^C,

[4]With the exception of states which represent the halting state.

and the one for the Gottesman-Irani Hamiltonian, V^{GI}, to get an isometry which applies an RG procedure for H_u. However, V^{GI} is only defined for a 1D Hamiltonian while V^C acts on a 2D lattice; furthermore, the action of V^{GI} is defined on $\mathcal{H}_q$ while the full Hilbert space that must be considered is $(\mathcal{H}_q \oplus |e\rangle)$, where $|e\rangle$ is a trivial filler state. This approach is thus not viable. Noting that the local terms have the following structure on a 2D lattice,

$$h_u = h_T^{(i,i+1)} \otimes \mathbb{1}_{eq}^{(i)} \otimes \mathbb{1}_{eq}^{(i+1)} + \mathbb{1}_c^{(i)} \otimes \mathbb{1}_c^{(i+1)} \otimes h_q^{(i,i+1)} + \text{ coupling terms},$$

we instead devise the following strategy:

1. Choose a 2×2 block of spins.
2. Renormalise the classical tiling part of the Hamiltonian by conjugating with V^C.
3. We extend the definition of V^{GI} so that it essentially acts trivially on the state $|e\rangle$.
4. To renormalise the quantum part of the Hamiltonian, break the 2×2 block into two 2×1 blocks (i.e. two rows of length 2). Renormalise these two rows of the block separately, as per RG scheme for the Gottesman-Irani Hamiltonian using V^{GI}. The 2×2 block is now a 1×2 block (i.e. a column of height 2). Then map both sites in the column to a single site.
5. Trace out part of the Hilbert space such the new site is still a single site, but the size of the Hilbert space is reduced. Furthermore, the trace operation is performed such that we are left with 1-local and 2-local projector terms which introduce an energy shift. This energy shift exactly compensates for any energy contribution lost in removing the states.

We can implement step 4 by defining the isometry

$$V^{eq}_{(i,i+1)(j,j+1)} := W_{(i,i+1)(j,j+1)} \left(V^q_{(i,i+1)}(j) \otimes V^q_{(i,i+1)}(j+1) \right), \tag{B.1}$$

where the bracketed j and $j+1$ refer to the row the isometry $V^q_{(i,i+1)}$ acts on, and where $W_{(i,i+1)(j,j+1)}$ combines the two renormalised rows into a single

site on the new lattice. Then the overall isometry for both the classical and quantum part of the Hamiltonian is

$$V^b_{(i,i+1)(j,j+1)} = V^C_{(i,i+1)(j,j+1)} \otimes V^{eq}_{(i,i+1)(j,j+1)}. \tag{B.2}$$

Finally we need to slightly modify this to implement step 5, so we multiply $V^b_{(i,i+1)(j,j+1)}$ with a projector which projects out history states. This gives us an overall renormalising isometry unitary $V^u_{(i,i+1)(j,j+1)}$ (see Definition 30). Now, conjugating the relevant terms with this isometry gives

$$H_u \to \bigotimes_{i,j\in 2\mathbb{N}} V^{u\dagger}_{(i,i+1)(j,j+1)} H_u \bigotimes_{i,j\in 2\mathbb{N}} V^u_{(i,i+1)(j,j+1)} \tag{B.3}$$

which has local interaction terms with the same structure as the original Hamiltonian:

$$R(h_u) = R(h_T)^{(i,i+1)} \otimes R(\mathbb{1})^{(i)}_{eq} \otimes R(\mathbb{1})^{(i+1)}_{eq} + R(\mathbb{1})^{(i)}_c \otimes R(\mathbb{1})^{(i+1)}_c \otimes R(h_q)^{(i,i+1)}$$
$$+ \text{renormalised coupling terms},$$

where the identity terms now act on the renormalised Hilbert space. In particular, R preserves many of the important properties of the Hamiltonian, including the product state structure, and approximately preserves the ground state energy. Furthermore, the ground state still consists of a product state of a classical Robinson tiling with a quantum state, such that renormalised Gottesman-Irani ground states run along the top of the Robinson squares, as required by construction.

We summarise these points in the following lemma.

Lemma 5 (H_u Renormalisation (Informal version of Lemma 33))**.** *Let* $H_u(L) = \sum h_u^{row(j,j+1)} + \sum h_u^{col(i,i+1)}$. *Then the k times renormalised Hamiltonian* $R^{(k)}(H_u)^{\Lambda(L\times W)}$ *has the following properties:*

1. *For any finite region of the lattice, the restriction of the Hamiltonian to that region has an eigenbasis of the form* $|T\rangle_c \otimes |\psi_i\rangle$ *where* $|T\rangle_c \in R^{(k)}(\mathcal{H}_c)^{\Lambda(L\times W)}$ *is a classical tiling state.*

2. *Furthermore, for any given* $|T\rangle_c \in R^{(k)}(\mathcal{H}_c)^{\Lambda(L\times W)}$*, the lowest energy choice for* $|\psi\rangle_q \in R^{(k)}(\mathcal{H}_{ud})^{\Lambda(L\times W)}$ *consists of renormalized Gottesman-Irani ground states between certain (renormalised) Robinson tiling, such*

that the pattern is self-similar after renormalisation.

3. *The ground state energy is approximately preserved.*

Full details are provided in Subsection E.1 (in particular Definition 31) and Subsection E.2 together with Section F, illustrating respectively Lemma 33 and the proof thereof.

Renormalising the Entire Hamiltonian The overall renormalisation scheme acts on H_u as above, and essentially leaves H_d and $|0\rangle\langle 0|$ unchanged. In other words, H_u, H_d and $|0\rangle\langle 0|$ have their respective ground state energies preserved (approximately) under the RG procedure, regardless of whether the ground state is $|0\rangle^\Lambda$ or the more complex ground state of $\sum R^{(k)}(h_u)_{i,j}$, is preserved.

The RG process can then be iterated arbitrarily many times: we show the relevant properties — and in particular, the spectral gap — are preserved throughout. The key argument to our uncomputability result (Theorem 42) is that, since determining the properties of the ground state and spectral gap is undecidable for the unrenormalised Hamiltonian, and since these properties are preserved by the RG mapping (Lemma 39), it is then also undecidable for the renormalised Hamiltonians. We highlight the fact that the RG flow constructed here satisfies all the desired features of an RG flow list in Theorem 1 of the main manuscript, which was indeed a fundamental prerequisite to our result. Furthermore, we show that the renormalised Hamiltonian belongs to a family of Hamiltonian characterised by a set of parameters, and that at least one of these parameters has an uncomputable trajectory under the RG flow (Theorem 42).

The renormalisation of the entire Hamiltonian is given in detail in Subsection E.5. The proof of our main theorems, namely, Theorem 41 (the formal version of Theorem 1) and Theorem 42, are given in Subsection E.7.

C Renormalisation of the tiling Hamiltonian

C.1 Robinson Tiling

Robinson tiles are 28 square tiles of unit length with markings on each side, together with rules stipulating that a pair of tiles can only be placed next to

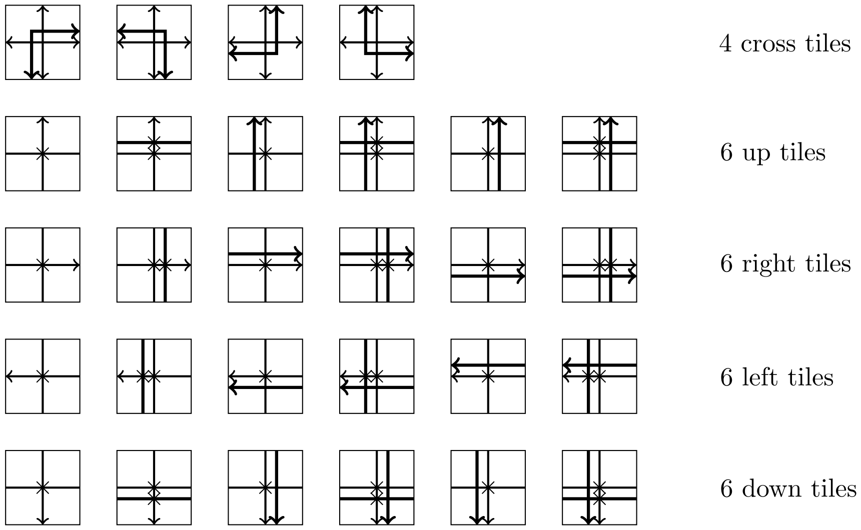

Supplementary Figure 1: the set of (modified) Robinson tiles used in our construction to encode the ground state of the classical Hamiltonian.

each other if the markings on their adjacent sides are compatible. That is, the head(s) of the arrow(s) in one tile and the tail(s) of the arrow(s) in the other tile must match exactly on the edges put into contact; we refer to [15] for a complete description. We will however consider a slightly modified version of Robinson tiles, used in [1], that we illustrate in Supplementary Fig. 1.

Following Robinson, these arrow markings are augmented with 4 parity tiles in a way that gives rise to 56 total different tiles. More precisely, we consider the coloured tiles given in Supplementary Fig. 2, which are called *parity tiles*, yielding an additional adjacency rule stating that only borders with the same colour can be placed next to each other.

Each parity tile can then be thought of as being coupled with a Robin-

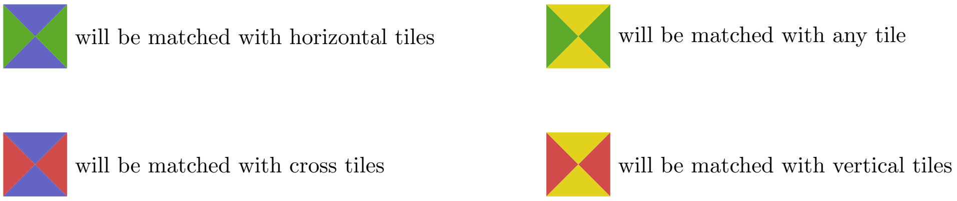

Supplementary Figure 2: the four parity tiles associated to Robinson tiles.

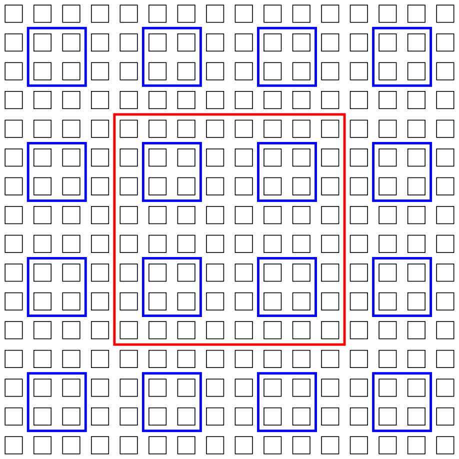

Supplementary Figure 3: Tiling pattern for the classical ground state $|T\rangle_c$.

son tile, hence forming a 2-layer tile. In this way, two tiles are allowed to be placed next to each other only if both their arrow markings and parity colours match along the edge in contact. When placed on a 2D grid such that the tiling rules are satisfied, the markings on the tiles form an aperiodic pattern consisting of a series of nested squares of sizes $4^n + 1$, for all $n \in \mathbb{N}$, as shown in Supplementary Fig. 3.

We will use the following terminology. We will call "Robinson tiles" the modified 2-layer tiles with both arrow marking and parity colours. Cross tiles matched with the red/blue parity tile will be called "parity crosses"; horizontal tiles coupled with green/blue tiles will be denoted as "parity horizontal" and analogously vertical tiles linked to red/yellow parity will be called "parity vertical". Conversely, any tile associated to the green/yellow tile will be called "free", so we will have "free crosses / horizontal / vertical" tiles.
When building adjacency rules, both arrow and parity rules must be obeyed.

Parity tiles will force the following structure. Considering the plane as a grid of cells where the tiles are to be placed, then parity cross tiles will appear in alternating rows and alternating columns. The same applies for parity horizontal and parity vertical tiles. Thus, if we consider a grid of 2×2 blocks over the plane, each 2×2 supertile will have the same inner parity structure. Depending on where we place the grid, we will obtain one of the configurations illustrated in Supplementary Fig. 4a, repeated over the whole plane.

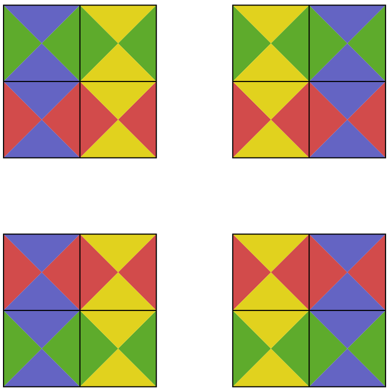

(a) The four parity structures of a 2× 2 cell

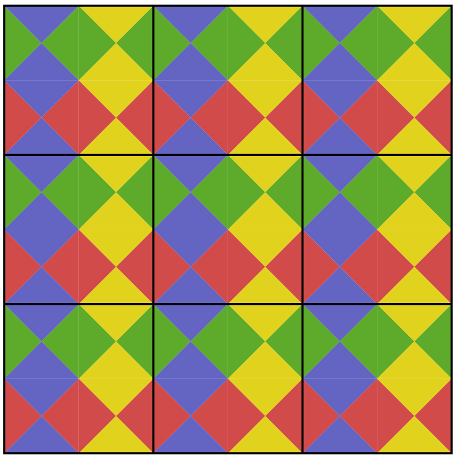

(b) pattern of the parity structure of the plane

Supplementary Figure 4: parity tiles pattern.

C.2 Tiling Renormalisation

In the following we will construct an RG map under which the two graphs representing respectively the *adjacency relations* (roughly speaking, the rules telling us what tiles can stay above / below / left / right of a given tile) for the Robinson tiles and for a specific subset of $2{\times}2$ supertiles are isomorphic. This implies that the pattern produced by the tiling of the 2D plane using Robinson tiles is scale-invariant. This property is crucial in order to ensure that the density of the Gottesman-Irani ground states (corresponding to the top edges of the squares appearing in the pattern) which encode the QTM is preserved under the renormalisation procedure.

We start the BRG construction of the tiling by imaging to place a grid over the pattern. When increasing the grid size, we go from 1×1 Robinson tiles to 2×2 supertiles. As we noted above, depending on the positioning of the grid, we will obtain one of the inner parity structures given in Supplementary Fig. 4a . From this point we will consider the first supertile on the top-left in Supplementary Fig. 4a, that is, the one with the parity cross on the bottom-left. The parity structure of the plane will then look as shown in Supplementary Fig. 4b. Note that, as explained in Subsection C.5, the other three options for the placement of the grid will produce equivalent results.

With this parity structure in mind, we generate all $2{\times}2$ supertiles permitted by the arrows rules. There are a total of 68 such supertiles, that we will

call *allowed supertiles*. Our aim is to identify a bijection between a subset of these 56 supertiles and the Robinson tiles. In other words, we consider the adjacency relations of the 2×2 tiles: they will generate a *directed graph*. We want to prove that this graph is isomorphic to the one describing the relations of the original Robinson tiles. We achieve this by trying to replicate the tiling pattern of the 2D plane with 2×2 supertiles, as described in detail in Subsection C.3. Interestingly, from this approach we observe that we can formulate the projection from 2×2 to 1×1 tiles by looking at the two tiles that occupy the bottom-left and the top-right position of the supertile. Indeed, once the tiles on the bottom-left and top right position are placed, there is only one possible choice for the two remaining tiles, which must also obey the inner parity structure of the supertile (see Supplementary Fig. 4a). This fact leads to the following definition of the renormalisation map.

Definition 6 (Tiles Renormalisation Map)**.** *Given an allowed 2×2 supertile, we consider its top-right tile with free parity, that we denote by T, and the parity cross on the bottom-left position, that we call C. The associated Robinson tile under the renormalisation map has the same marking as T and parity characterised by C according to the correspondence given in Supplementary Fig. 5.*

Under this projection, 12 supertiles (illustrated in Subsection C.4) are not mapped to any Robinson tile. The reason for this is that there does not exist a Robinson tile with matching of arrows and parity: the supertiles of type 1 in Supplementary Fig. 6 would be mapped to vertical arms with horizontal parity, and conversely type 2 supertiles would correspond to hori-

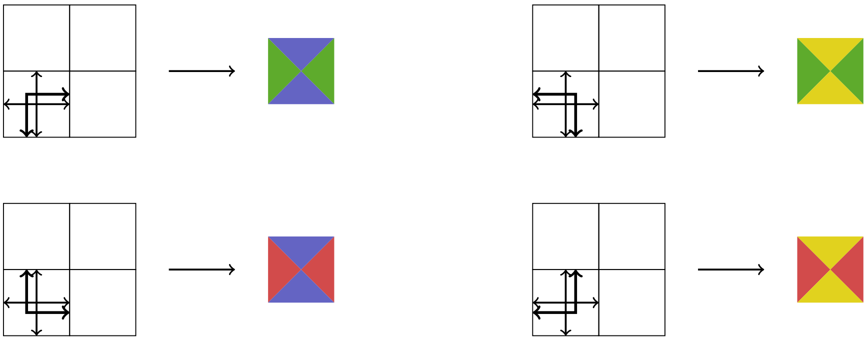

Supplementary Figure 5: Correspondence between supertiles and parity tiles under the RG mapping.

zontal arms with vertical parity. Crucially, none of these supertiles appear in the pattern of the plane. Hence, for our renormalisation procedure we shall consider only a subset of 56 supertiles. We have verified in a *Mathematica* notebook (cfr. Subsection C.3.1) that, under the map in Definition 6, the adjacency relations of these 56 allowed supertiles and their corresponding Robinson tiles are equivalent, proving the following result.

Theorem 7. *(Adjacency Relations Isomorphism) Let T_1 be the set of Robinson tiles and A_1 be the corresponding adjacency relations. Let T_2 be the set of 2×2 supertiles, obtained from all combinations allowed by A_1 of four Robinson tiles placed in a 2×2 square, and A_2 be the adjacency relations of T_2, derived from the principle that two supertiles can be placed next to each other only if the Robinson tiles on the edges that are put adjacent respect A_1. Let $T'_2 \subset T_2$, $|T'_2| = |T_1| = 56$, be the subset of supertiles finding a correspondent Robinson tile under the RG map in Definition 6 with relations $A'_2 = A_2|_{T'_2}$. Then, A_1 and A'_2 are equivalent.*

From this result it follows that

Corollary 8. *(Scale Invariance of the Robinson Tiling) Under the RG map in Definition 6 the Robinson tiling pattern is preserved.*

C.3 Reconstructing Robinson pattern of 2D plane

A first interesting fact is that 2×2 supertiles having a parity cross on the bottom left pointing up-right must have the following structure. A parity left tile on top-left corner, a parity down tile in the bottom-right corner, and consequently there must be a free cross on the top-right of the supertile. The orientation of the free cross will uniquely determine the type of left tile and down tile in the same supertile. Thus, there are only 4 supertiles with a parity cross pointing up-right:

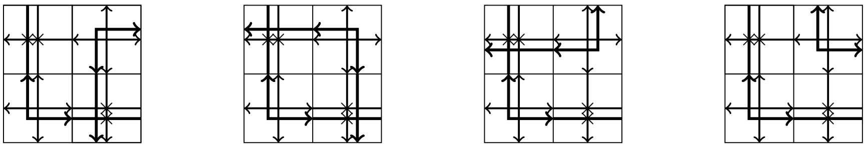

We make a first educated guess: each of these four supertiles corresponds to the 1×1 parity cross having the same orientation as the free cross contained in the top-right of the supertile.

If we want to tile the plane according to the Robinson pattern, these supertiles must then appear in alternate positions in alternate rows. To this aim, we will assign parity rules to the supertiles according to the orientation of the 1×1 parity cross on the bottom-left of each supertile, as shown in Supplementary Fig. 5 (note: this is a parity associated to the supertile as a whole and it is different to its inner parity structure).

Thus, 2×2 supertiles with a up-right 1×1 parity cross in the bottom left must be interleaved in the vertical direction with a supertile with a 1×1 parity cross pointing bottom-right and in the horizontal direction with a supertile having a 1×1 up-left parity cross. Finally, supertiles with a down-left cross will alternate on the diagonal with the supertiles having a up-right cross.

Using these parity rules and the usual arrow heads/tails constraints, we shall obtain the adjacency relations for the supertiles which have to be obeyed. We make a point here: the only constraints that we will use in the tiling of the 2D plane are the ones set by these adjacency rules.

We shall now reconstruct the basic 3-square in Robinson's argument, this time using 2×2 supertiles. At the corners of these 3-squares there must be the four supertiles that we have identified as parity crosses. By strictly following the adjacency rules, we will end up with exactly four possible 3-squares, that we will relate to the 3-squares with 1×1 tiles. No other configuration of a 3-square is allowed!

(1)

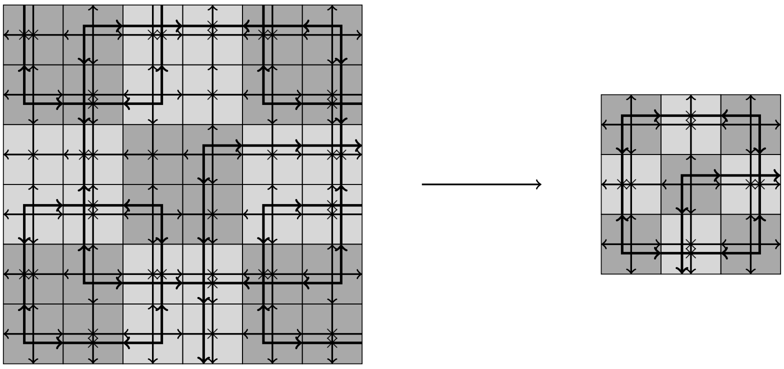

(2)

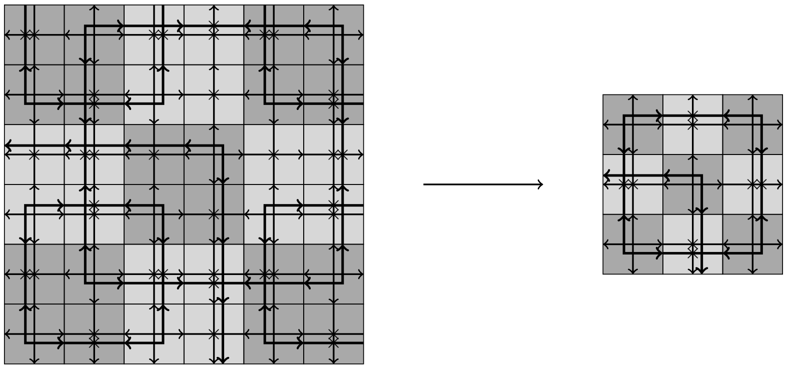

(3)

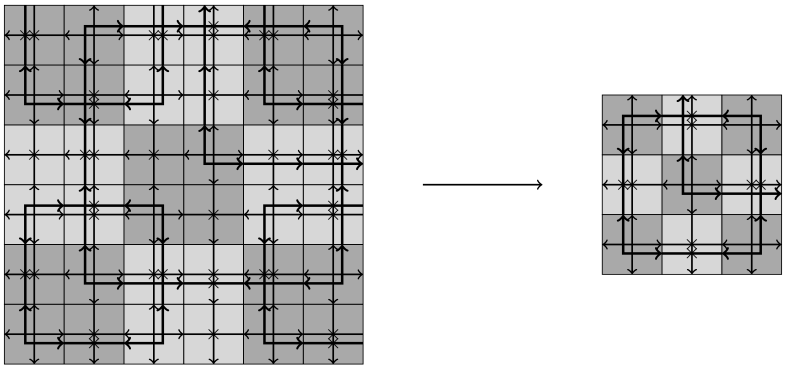

(4)

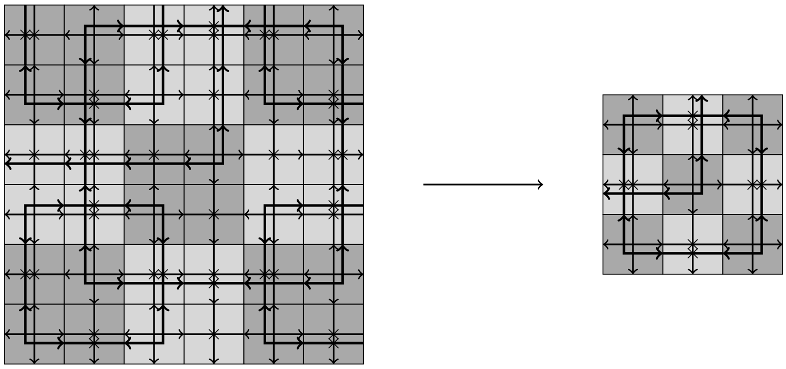

We remark that the position of the 2×2 parity crosses at the corner of the 3-square are fixed, and that the central 2×2 supertile of these 3-squares

— corresponding to one of the 1×1 free crosses — uniquely determines the remaining ones.

So far we have recognised the first 20 tiles. Among these are the parity cross and free cross tiles. The remaining ones will be determined by those supertiles placed between the 3-squares, again in analogy to the Robinson pattern.

We consider the 3-squares in the illustration above, labelled from 1 to 4. When we pick the first 3-square, we note that only the second 3-square can be placed at its right, and they must be interleaved with a string made of three supertiles put in vertical order: only three configurations for these strings are allowed. Below we illustrate their arrow markings as well as their renormalisation onto Robinson tiles. Note that the central Robinson tile in each renormalised string has free parity and is hence different from the parity vertical tile having the same arrow markings appearing in the 3-squares. Note also that the tile at the top of each string is the identical to the one at its bottom (both in markings and parity).

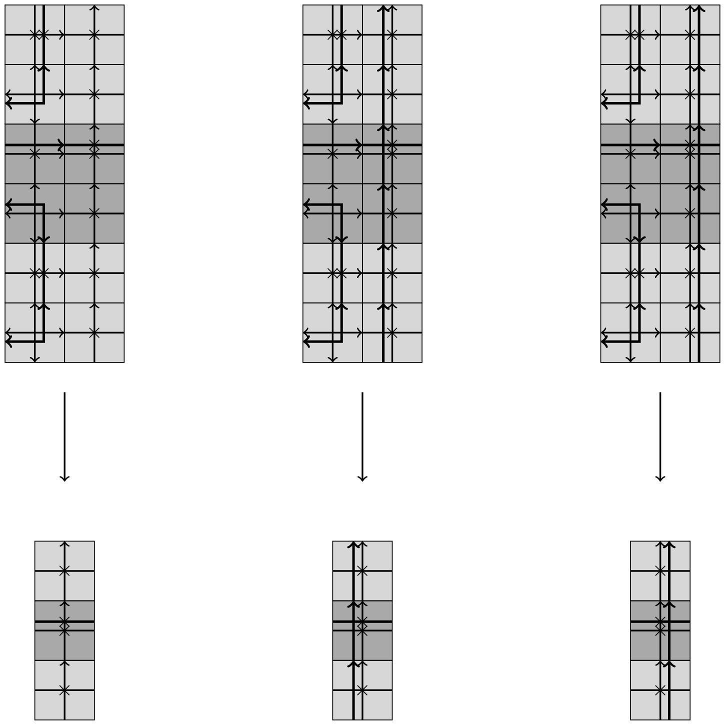

Analogously, starting from the third 3-square only the forth one is allowed to be placed at its right, and between them there must be one of the following strings of supertiles, that we renormalise as illustrated below.

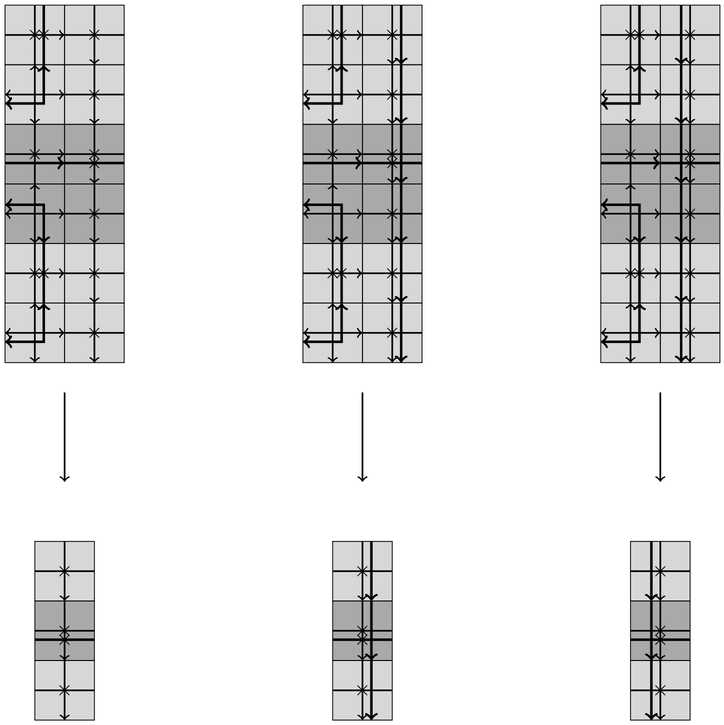

Vertical Robinson tiles with free parity and a single horizontal line correspond to supertiles between 3-squares whose free crosses do not face each others. More precisely, the mapping is given by

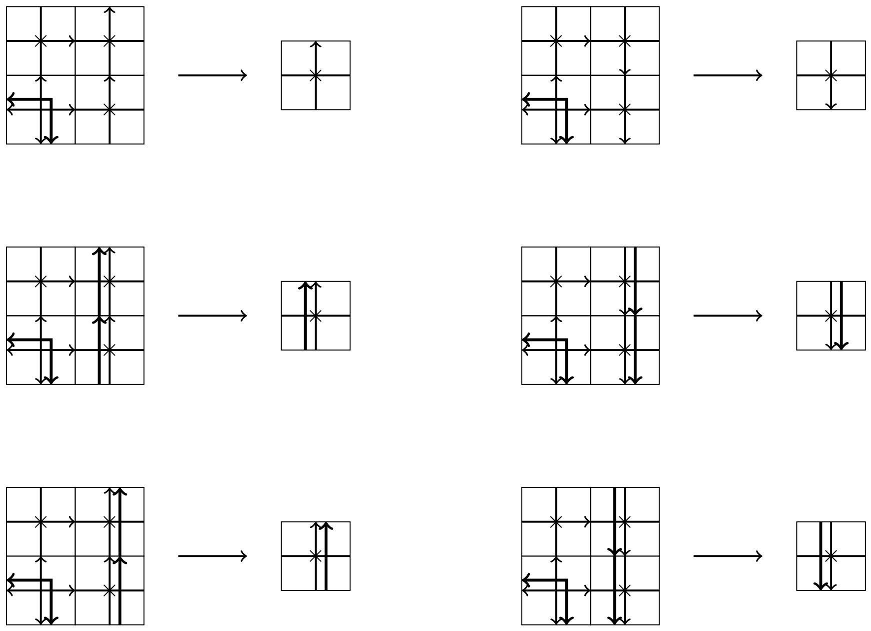

We have at this point a correspondence between 38 tiles and 38 supertiles; in addition to parity crosses and free crosses, now all vertical arms associated with both parities have been identified. The remaining 18 tiles are horizontal arms. To find them, we proceed in analogous way.

Below the first 3-square we can place only the third 3-square, interleaved with one of the following strings of three supertiles put in horizontal order. Again, we note that the left and right supertiles of each string coincide and are thus mapped to the same Robinson tile and that the central tile has free parity.

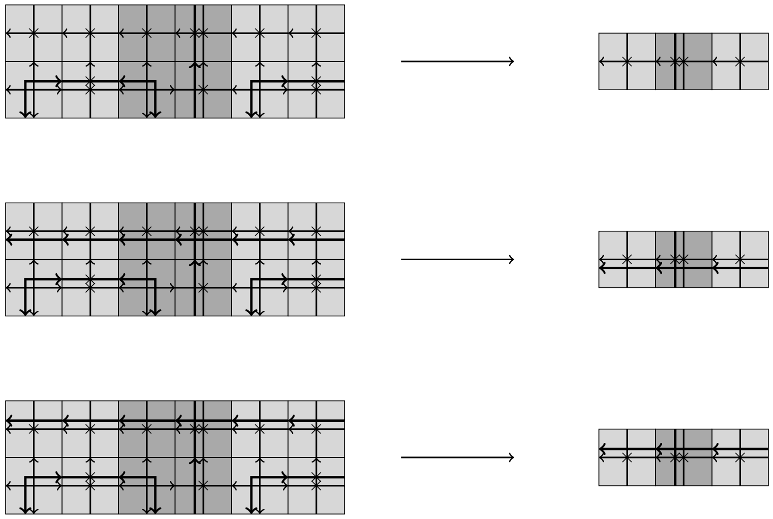

We have other three strings of supertiles that are allowed to stay between the second three square placed above the fourth 3-squares.

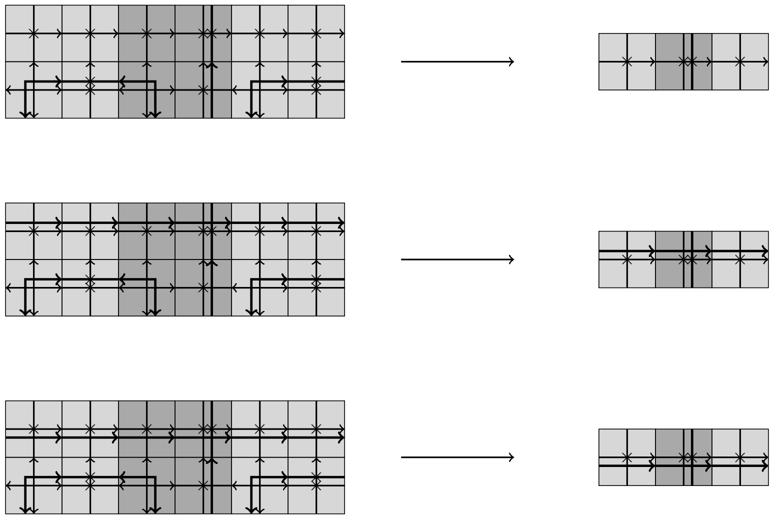

It remains to associate the last 6 free horizontal Robinson tiles to the supertiles that are still unmatched. These must be placed between 3-squares whose free crosses do not face each other.

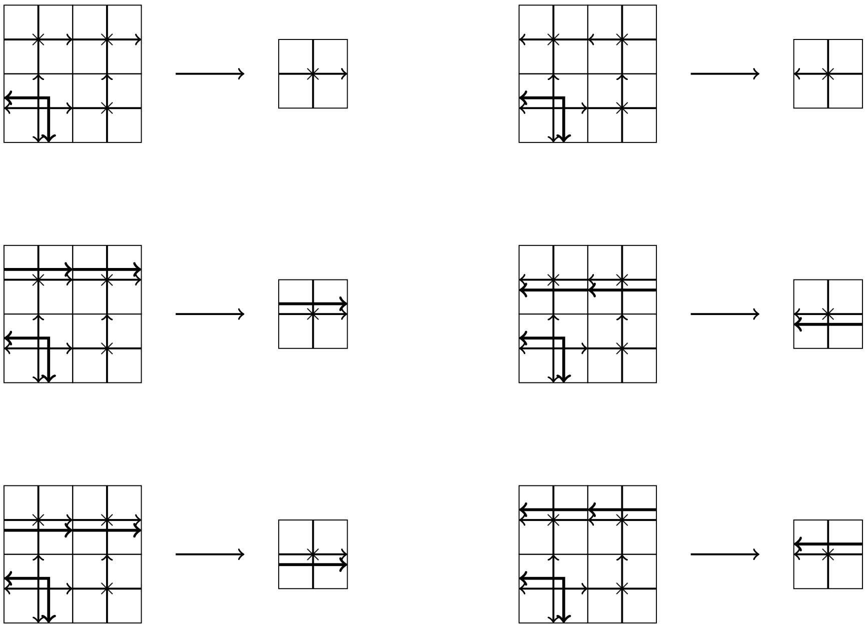

All 56 Robinson tiles have finally been identified with a subset of the allowed 2×2 supertiles. With this renormalisation, one can verify that it is possible to reproduce the Robinson pattern of the plane with $2{\times}2$ supertiles. More importantly, one can ascertain that the adjacency rules for the 2×2 supertiles, under this projection, correspond exactly to the rules for the Robinson tiles (cfr. Subsection C.3.1). Stated in another way, the directed graphs representing respectively the adjacency rules of the 56 supertiles and the rules for the Robinson tiles are isomorphic. Thus, we have achieved a complete renormalisation under which Theorem 7 and Corollary 8 hold.

C.3.1 Mathematica notebook

Available as a supplementary file is a *Mathematica* notebook for the explicit construction of the renormalisation map in Definition 6, in the case where the parity cross occupies the bottom-left position of the grid.[5]

We implement by hand the adjacency relations for the Robinson tiles, where the tiles are numbered according to the order given in Supplementary Fig. 1, from left to right, top to bottom. For each tile we list what are

[5]The notebook is available at https://gitlab.com/ucl_cs_quantum/RG_flows_uncomputability

the ones that can stay above (variable *adjup1x1* in the notebook) or on its right (variable *adjright1x1*), respectively. Using these rules, we then construct all allowed supertiles with a parity cross in the bottom-left position; the total number of those new elements is 68. In the notebook, each supertile is represented by a 2×2 matrix whose entries are numbers from 1 to 56 corresponding to the Robinson tiles which are composing it. We then construct adjacency relations for these supertiles by obeying arrow markings and parity constraints on the shared edge.

The renormalisation map in Definition 6 is represented by the variable *labelrenormalization*: the number at the position j corresponds to the Robinson tile associated to the supertile j. The supertiles that are not appearing in the Robinson pattern discussed in Subsection C.4 are mapped to numbers from 57 to 68; these are not connected to any Robinson tile, and subsequently removed from the bijection. Finally, we re-arrange the adjacency relations for supertiles under this bijection and confirm that the graph is isomorphic to the one of the Robinson tiles.

C.4 Allowed but not appearing supertiles

Consider the subset of supertiles allowed by the adjacency rules which have a 1×1 parity cross in the bottom-left. There are 68 such tiles, however, there are only 56 Robinson tiles. The result of this is that 12 tiles cannot be mapped under the renormalisation procedure. These have two distinct structures, as shown in Supplementary Fig. 6, where we have used the abbreviated notation used in [15], indicating only the direction of the arms.

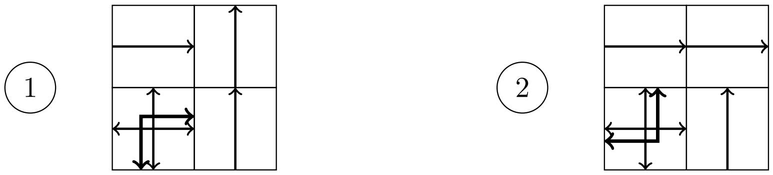

Supplementary Figure 6: allowed supertiles not appearing in the pattern.

For each of these two structures, we have 6 possible combinations. Importantly, these supertiles cannot appear in any tiling of the plane and thus it is not necessary to design our RG procedure in order to incorporate them. Consider the structure 1. By imposing the parity rules for supertiles that we

described previously, a supertile of type 1 there must have one of the parity cross supertiles above it, which has the (abbreviated) form shown in Supplementary Fig. 7. Clearly, no supertile with the structure 1 can be placed below a parity cross supertile because of the arrow rules. Analogously, by parity rules, on the right of a supertile with structure 2 must lie a parity cross supertile. Again, it is clear that this is not allowed by the arrow rules.

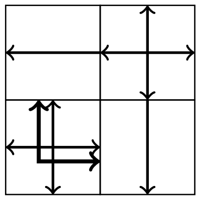

Supplementary Figure 7: supertile with two crosses in the tiling pattern.

C.5 Shifting the Grid

The previous analysis was done by placing a 2×2 grid over the Robinson pattern on the plane with the parity cross lying on the bottom-left of each 2×2 cell. Naturally, there are 4 possible ways that we could place our 2×2 grid. The same investigation has been performed for all other three cases when shifting the 2×2 grid one cell right, upwards, and diagonally, so that the parity cross will occupy the bottom-right, top-left and top-right position of the supertiles, respectively. The analysis for these other settings is completely equivalent to the case we have discussed. For any of the four positioning of the parity cross in the 2×2 grid, there exist 68 allowed supertiles, and a subset of 56 will tile the plane and build adjacency relations isomorphic to the Robinson tiles.

Remark 9. *The four possible placements of the 2×2 grid give rise to four completely disjoint sets of 68 supertiles each. This is a direct consequence of the different inner parity structure of the supertiles illustrated in Supplementary Fig. 4a.*

Let the set of possible renormalised supertiles, but restricted to those which actually appear, be denoted:

$$\mathcal{T}_1 \oplus \mathcal{T}_2 \oplus \mathcal{T}_3 \oplus \mathcal{T}_4. \tag{C.1}$$

which each $\mathcal{T}_i$ corresponding to a different supertile parity structure. Then the set of supertiles that occurs for our choice of basis, T_2', is equal to one of these four disjoint sets. Which set occurs depends on where the 2×2 grid is placed.

C.6 Renormalising the Classical Hamiltonian

We are now in a position to show that there is an RG transformation on the tiling Hamiltonian which preserves the ground state. In a nutshell, the RG scheme takes the form of restricting to sets of allowed 2×2 blocks, and then applies an isometry mapping these 2×2 blocks to new supertiles belonging to the set T_2'.

The Initial Tiling Hamiltonian

Let $h_T^{row} = \sum_{(t_i,t_j)\notin A_1^H} |t_i t_j\rangle\langle t_i t_j|$ and $h_T^{row} = \sum_{(t_i,t_j)\notin A_1^V} |t_i t_j\rangle\langle t_i t_j|$ be the local interaction terms of the tiling Hamiltonian, where A_1^H and A_1^V are, respectively, the horizontal and vertical adjacency rules for tiles in T_1. Then the ground state of $H = \sum_{i\in\Lambda(L)} h_{T,i,i+1}^{row} + \sum_{j\in\Lambda(L)} h_{T,j,j+1}^{col}$ has a zero energy ground state which is given by the tiling of the plane according to the Robinson pattern.

We now construct the RG scheme for the Hamiltonian. We first define the following isometry.

Definition 10 (Tiling Renormalisation Isometry)**.** *Let T_2' be one of the disjoint subsets of 2×2 Robinson tiles which appear in the previously described renormalisation scheme. Let $V_{(i,i+1),(j,j+1)} : T_1^{\otimes 2\times 2} \to T_2'$ be the isometry mapping these 2×2 blocks to allowed supertiles which appear in the Robinson pattern,*

$$V_{(i,i+1),(j,j+1)} = \sum_{|T_\alpha\rangle\in T_2'} |T_\alpha\rangle_{I,J} \langle t_a|_{i,j} \langle t_b|_{i+1,j} \langle t_k|_{i,j+1} \langle t_l|_{i+1,j+1}, \qquad \text{(C.2)}$$

where $|t_m\rangle \in T_1$, with the set of T_2' tiles that maps to the Robinson tiles as described in Definition 6.

Then we have:

Definition 11 (Tiling Hamiltonian Renormalisation)**.** *Let $h_T^{col}, h_T^{row} \in \mathcal{B}(\mathbb{C}^T \otimes \mathbb{C}^T)$ be the local interactions describing the tiling Hamiltonian. Let $h_{i,i+1}^{row}(j)$ denote the row interaction between sites (i,j), $(i+1,j)$ and similarly let $h_{j,j+1}^{col}(i)$*

be the interaction between $(i,j),(i,j+1)$. Let the 2×2 supertiles be assigned at $(i,j),(i+1,j),(i,j+1),(i+1,j+1)$ and sites consistent with it. Then the renormalised Hamiltonian has local terms $R(h_T^{col}), R(h_T^{col}) \in \mathcal{B}(\mathbb{C}^T \otimes \mathbb{C}^T)$.

$$\begin{aligned}
R(h_T^{col})_{\lceil j/2-1\rceil,\lceil j/2-1\rceil+1} &= V_{(i,i+1),(j+2,j+3)} V_{(i,i+1),(j,j+1)} \left(h^{col}_{T,j+1,j+2}(i) + h^{col}_{T,j+1,j+2}(i+1)\right)\big|_{T_2'} \\
&\quad \times V^\dagger_{(i,i+1),(j,j+1)} V^\dagger_{(i,i+1),(j+2,j+3)} \\
R(h_T^{row})_{\lceil i/2-1\rceil,\lceil i/2-1\rceil+1} &= V_{(i+2,i+3),(j,j+1)} V_{(i,i+1),(j,j+1)} \left(h^{row}_{T,i+1,i+2}(j) + h^{row}_{T,i+1,i+2}(j+1)\right)\big|_{T_2'} \\
&\quad \times V^\dagger_{(i,i+1),(j,j+1)} V^\dagger_{(i+2,i+3),(j,j+1)}
\end{aligned}$$

In the above we have used the standard abbreviation that each local term is implicitly tensored with the appropriate identity terms, e.g. $h^{col}_{T,j+1,j+2}(i)$ is actually $\mathbb{1}_{i,j} \otimes \mathbb{1}_{i+1,j} \otimes h^{col}_{T,j+1,j+2}(i) \otimes \mathbb{1}_{i,j+3} \otimes \mathbb{1}_{i+1,j+3}$.

Note that this renormalisation map is computable, as each V simply describes the mapping of tiles in the initial set to those in the new set in the way illustrated previously.

We now prove that the local Hamiltonian terms are mapped back onto themselves when this RG transformation is applied.

Lemma 12. *The matrix form of the initial and renormalised Hamiltonian are the same, i.e.,*

$$R(h_T^{row})_{i,i+1} = h^{row}_{T,i,i+1} \qquad \textit{and} \qquad R(h_T^{col})_{j,j+1} = h^{col}_{T,j,j+1}. \tag{C.3}$$

Proof. We consider two neighbouring 2×2 blocks $(i,j),(i+1,j),(i,j+1),(i+1,j+1)$ and $(i+2,j),(i+3,j),(i+2,j+1),(i+3,j+1)$, and determine how the row and column interactions transform under this renormalisation process. We can then write

$$h^{row}_{i,i+1}(j) = \sum_{(t_k,t_l\in H)} |t_k\rangle_{i,j} |t_l\rangle_{i+1,j} \langle t_k|_{i,j} \langle t_l|_{i+1,j} \tag{C.4}$$

and, with $|T_\alpha\rangle \in T_2'$, then

$$V_{(i,i+1),(j,j+1)} = \sum_{|T_\alpha\rangle\in T_2'} |T_\alpha\rangle \langle t_a|_{i,j} \langle t_b|_{i+1,j} \langle t_k|_{i,j+1} \langle t_l|_{i+1,j+1}\,, \tag{C.5}$$

where $|t_a\rangle_{i,j} |t_b\rangle_{i+1,j} |t_k\rangle_{i,j+1} |t_l\rangle_{i+1,j+1}$ is an allowed 2×2 supertile, and we sum over all such allowed 2×2 blocks.

Now consider the two blocks: there are 6 relevant row interactions, namely

$$h^{row}_{i,i+1}(j) + h^{row}_{i,i+1}(j+1) \tag{C.6}$$
$$+h^{row}_{i+1,i+2}(j) + h^{row}_{i+1,i+2}(j+1) \tag{C.7}$$
$$+h^{row}_{i+2,i+3}(j) + h^{row}_{i+2,i+3}(j+1). \tag{C.8}$$

Restrict to the set of appearing 2×2 supertiles, T_2', which are centred on the 2×2 blocks $(i,j), (i+1,j), (i,j+1), (i+1,j+1)$ and $(i+2,j), (i+3,j), (i+2,j+1), (i+3,j+1)$. In this case we see that by enforcing only allowed supertiles, then $(h^{row}_{i,i+1}(j)+h^{row}_{i,i+1}(j+1))|_{T_2'} = 0$ and $(h^{row}_{i+2,i+3}(j)+h^{row}_{i+2,i+3}(j+1))|_{T_2'} = 0$.

Finally we need to consider the terms

$$V_{(i+2,i+3),(j,j+1)} V_{(i,i+1),(j,j+1)} (h^{row}_{i+1,i+2}(j) + h^{row}_{i+1,i+2}(j+1))|_{T_2'} \tag{C.9}$$
$$\times V^\dagger_{(i,i+1),(j,j+1)} V^\dagger_{(i+2,i+3),(j,j+1)}. \tag{C.10}$$

The application of the isometries maps the tiles to supertiles. Hence we can write

$$R(h^{row})_{i/2,i/2+1}(j) = V_{(i,i+1),(j,j+1)} V_{(i+2,i+3),(j,j+1)} \left(h^{row}_{i+1,i+2}(j) + h^{row}_{i+1,i+2}(j+1)\right)|_{T_2'} \times \tag{C.11}$$
$$\times V^\dagger_{(i,i+1),(j,j+1)} V^\dagger_{(i+2,i+3),(j,j+1)}. \tag{C.12}$$

Note that $R(h^{row})_{i,i+1}(j)$ acts on T_2' and we see that there is an energy assigned to a particular term in $R(h^{row})_{i,i+1}(j)$ iff there is a corresponding term in $h^{row}_{i,i+1}$. Furthermore $R(h^{row})_{i,i+1}(j)$ is the same for all j, hence $R(h^{row})_{i,i+1} = h^{row}_{i,i+1}$. □

From this block RG scheme, our main theorem for the classical Hamiltonian renormalisation follows.

Theorem 13 (Robinson Tiling Hamiltonian Renormalisation)**.** *Let $h_T \in \mathbb{C}^T \otimes \mathbb{C}^T$ be the local interactions which describe the Robinson tiling Hamiltonian. Then there exists a renormalisation group mapping $\mathcal{R}_T$ satisfying $R_T(h_T) = h_T'$, where $h_T' \in \mathbb{C}^T \otimes \mathbb{C}^T$, such that $R_T(h_T)$ preserves both the ground state energy and the tiling pattern.*

D Renormalisation of the Quantum Hilbert Space

In this section we will deal with the renormalisation of the quantum Hamiltonian. For this, we will need a number of definitions from [1].

Definition 14 (Standard Basis States). *Let the single site Hilbert space be $\mathcal{H} = \otimes_i \mathcal{H}_i$ and fix some orthonormal basis for the single site Hilbert space. Label the set of single site basis states for site i as $\mathfrak{B}_q^{(i)}$. Then a* standard basis state *for $\mathcal{H}^{\otimes L}$ are product states over the single site basis.*

Definition 15 (Penalty Terms and Transition Rules). *The two-local quantum Hamiltonian will contain two types of terms:* penalty terms *and* transition rule terms. *Penalty terms have the form $|ab\rangle\langle ab|$ where $|a\rangle$ and $|b\rangle$ are standard basis states. This adds a positive energy contribution to any configuration containing the state $|ab\rangle$, which we call an* illegal pair. *Transition rule terms take the form $\frac{1}{2}(|ab\rangle - |cd\rangle)(\langle ab| - \langle cd|)$ with $|ab\rangle \neq |cd\rangle$, where $|ab\rangle$ and $|cd\rangle$ act on the same pair of adjacent sites.*

Definition 16 (Legal and Illegal States). *We call a standard basis state* legal *if it does not contain any illegal pairs, and* illegal *otherwise*

We then define a standard form Hamiltonian on the joint system

$$\mathcal{H}_C \otimes \mathcal{H}_Q \coloneqq (\mathbb{C}^C \otimes \mathbb{C}^Q)^{\otimes L} = (\mathbb{C}^C)^{\otimes L} \otimes (\mathbb{C}^Q)^{\otimes L}. \tag{D.1}$$

Definition 17 (Standard-Form Hamiltonian [1, 16]). *We say that a Hamiltonian $H = H_{trans} + H_{pen} + H_{in} + H_{out}$ acting on $\mathcal{H}_C \otimes \mathcal{H}_Q$ is of* standard form *if it takes the form*

$$H_{trans,pen,in,out} = \sum_{i=1}^{L-1} h_{trans,pen,in,out}^{(i,i+1)} \tag{D.2}$$

where the local interactions $h_{trans,pen,in,out}$ satisfy the following conditions:

1. *$h_{trans} \in \mathcal{B}\left((\mathbb{C}^C \otimes \mathbb{C}^Q)^{\otimes 2}\right)$ is a sum of transition rule terms, where all the transition rules act diagonally on $\mathbb{C}^C \otimes \mathbb{C}^C$ in the following sense. Given standard basis states $a, b, c, d \in \mathbb{C}^C$, exactly one of the following holds:*

   - *there is no transition from ab to cd at all; or*

- *$a, b, c, d \in \mathbb{C}^C$ and there exists a unitary U_{abcd} acting on $\mathbb{C}^Q \otimes \mathbb{C}^Q$ together with an orthonormal basis $\{|\psi^i_{abcd}\rangle\}_i$ for $\mathbb{C}^Q \otimes \mathbb{C}^Q$, both depending only on a, b, c, d, such that the transition rules from ab to cd appearing in h_{trans} are exactly $|ab\rangle|\psi^i_{abcd}\rangle \to |cd\rangle U_{abcd}|\psi^i_{abcd}\rangle$ for all i. There is then a corresponding term in the Hamiltonian of the form $(|cd\rangle \otimes U_{abcd} - |ab\rangle)(\langle cd| \otimes U^\dagger_{abcd} - \langle ab|)$.*

2. *$h_{pen} \in \mathcal{B}\left((\mathbb{C}^C \otimes \mathbb{C}^Q)^{\otimes 2}\right)$ is a sum of penalty terms which act non-trivially only on $(\mathbb{C}^C)^{\otimes 2}$ and are diagonal in the standard basis, such that $h_{pen} = \sum_{ab \text{ illegal}} |ab\rangle\langle ab|_C \otimes \mathbb{1}_Q$, where $|ab\rangle$ are members of a disallowed/illegal subspace.*

3. *$h_{in} = \sum_{ab} |ab\rangle\langle ab|_C \otimes \Pi_{ab}$, where $|ab\rangle\langle ab|_C \in (\mathbb{C}^C)^{\otimes 2}$ is a projector onto $(\mathbb{C}^C)^{\otimes 2}$ basis states, and $\Pi^{(in)}_{ab} \in (\mathbb{C}^Q)^{\otimes 2}$ are orthogonal projectors onto $(\mathbb{C}^Q)^{\otimes 2}$ basis states.*

4. *$h_{out} = |xy\rangle\langle xy|_C \otimes \Pi_{xy}$, where $|xy\rangle\langle xy|_C \in (\mathbb{C}^C)^{\otimes 2}$ is a projector onto $(\mathbb{C}^C)^{\otimes 2}$ basis states, and $\Pi^{(in)}_{xy} \in (\mathbb{C}^Q)^{\otimes 2}$ are orthogonal projectors onto $(\mathbb{C}^Q)^{\otimes 2}$ basis states.*

Importantly the Gottesman-Irani Hamiltonian we will be considering will be of standard form.

D.1 The Gottesman-Irani Hamiltonian

The particular QTM-to-Hamiltonian mapping encoded in h_u will be important when it comes to renormalising the overall Hamiltonian. We will follow the overall structure used in [1], which is a modification of the original construction from [3].

QTMs can be encoded into a 1D, translationally-invariant, nearest-neighbour Hamiltonian, which we refer to as *a Gottesman-Irani Hamiltonian*, denoted by $H_q(L) \in \mathcal{B}((\mathbb{C}^d)^{\otimes L})$. This is summarised by Theorem 32 of [1]; we write out a slightly simpler version here as the specific details are not important for our purposes. These constructions will be needed in order to formulate Lemma 23 for the block-renormalisation of the quantum Hamiltonian.

Theorem 18 (Informal Version of Theorem 32 of [1]).
Let $\mathbb{C}^d = \mathbb{C}^C \otimes \mathbb{C}^Q$ *be the local Hilbert space of a 1-dimensional chain of length* L*, with special marker states* $|\circledless\rangle, |\circledgtr\rangle$*. Denote the orthogonal complement of* $\mathrm{span}(|\circledless\rangle, |\circledgtr\rangle)$ *in* $\mathbb{C}^d$ *by* $\mathbb{C}^{d-2}$*. Let* d, Q *and* C *all be fixed.*

For any well-formed unidirectional Quantum Turing Machine $M = (\Sigma, Q, \delta)$ *and any constant* $K > 0$*, we can construct a two-body interaction* $h \in \mathcal{B}(\mathbb{C}^d \otimes \mathbb{C}^d)$ *such that the 1-dimensional, translationally-invariant, nearest-neighbour Hamiltonian* $H(L) = \sum_{i=1}^{L-1} h^{(i,i+1)} \in \mathcal{B}(\mathcal{H}(L))$ *on the chain of length* L *has the following properties:*

1. d *depends only on the alphabet size and number of internal states of* M.
2. $h \geq 0$*, and the overall Hamiltonian* $H(L)$ *is frustration-free for all* L.
3. *Denote* $\mathcal{H}(L-2) := (\mathbb{C}^{d-2})^{\otimes L-2}$ *and define* $\mathcal{S}_{br} = \mathrm{span}(|\circledless\rangle) \otimes \mathcal{H}(L-2) \otimes \mathrm{span}(|\circledgtr\rangle) \subset \mathcal{H}$*. Then the unique ground state of* $H(L)|_{\mathcal{S}_{br}}$ *is a computational history state (cf. eq. (A.9) for a definition) encoding the evolution of* M.

Moreover, the action of M *satisfies:*

1. *The computational history state always encodes* $\Omega(2^L)$ *time-steps. If* M *halts in fewer than the number of encoded time steps, exactly one* $|\psi_t\rangle$ *has support on a state* $|\top\rangle$ *that encodes a halting state of the QTM. The remaining time steps of the evolution encoded in the history state leave* M*'s tape unaltered, and have zero overlap with* $|\top\rangle$.
2. *If* M *runs out of tape within a time* T *less than the number of encoded time steps, the computational history state only encodes the evolution of* M *up to time* T*. The remaining steps of the evolution encoded in the computational history state leave* M*'s tape unaltered.*

We provide in the following a sketch of how the modified Gottesman-Irani construction works, and refer the reader to [1, 3] for a more detailed description. We begin by considering the general setup. The local Hilbert space at each site is the tensor product of the local Hilbert space of each of the six tracks $\mathcal{H} = \bigotimes_{i=1}^{6} \mathcal{H}_i$. To respect this structure, we choose our basis states for $(\mathbb{C}^d)^{\otimes L}$ (i.e. the chain of length L) to have the structure shown in Supplementary Fig. 8. That is, the basis consists of each of the 6 tracks at a particular time-step in the evolution of a QTM.

⧀	···	Track 1: Clock oscillator	···	⧁
⧀	···	Track 2: Counter TM head and state	···	⧁
⧀	···	Track 3: Counter TM tape	···	⧁
⧀	···	Track 4: QTM head and state	···	⧁
⧀	···	Track 5: QTM tape	···	⧁
⧀	···	Track 6: Time-wasting tape	···	⧁

Supplementary Figure 8: the six tracks of the QTM computation.

The outline of the construction is the following: tracks 1 encodes the evolution of an oscillator which goes back and forth along its track as per Supplementary Fig. 9. Tracks 2 and 4 contain the heads of a classical and quantum TM respectively. These heads are only able to move when the oscillator on track 1 passes by their heads — in this way their evolution can be encoded with only local Hamiltonian terms. Tracks 3 and 5 are the read/write tapes for the respective TMs.

The classical TM encoded by the track 2 head will be a simple counter: it will write out binary number on its tape (on track 3) and then increment it by one to the next binary number. This continues until the tape has the number of maximum possible value written on it (i.e. the value of the number written on the tape cannot be increased within the allowed tape space), at which point it halts along with the clock oscillator.

The QTM on tracks 4 and 5 will be a generic QTM. The QTM evolves as per its transition rules until either: (a) the counter TM runs out of space and hence the oscillator stops, or (b) the QTM finishes its computation and halts.

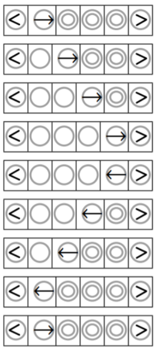

Supplementary Figure 9: evolution of the Track 1 clock oscillator.

If the QTM halts before the counter TM runs out of steps, it places a halting marker on track 5. The head then moves to track 6 where it performs some arbitrary time wasting computation which is guaranteed not to halt before the counter TM.

We also note that tracks 1-3 evolved entirely classically whereas tracks 4-6 will contain quantum states. As such, we decompose the local Hilbert space into a classical and quantum part $\mathbb{C}^C \otimes \mathbb{C}^Q$.

D.2 Subspace Structure of the Gottesman-Irani Hamiltonian

Lemma 43 of [1] identifies three subspaces of states for the Gottesman-Irani Hamiltonian, all of which which are closed under the action of H_q.

1. **Illegal Subspace**, $\mathcal{S}_1$: All $|x\rangle \in \mathcal{S}_1 \subset \mathfrak{B}^{\otimes L}$ are in the support of H_{pen} and hence $\langle x| H |x\rangle \geq 1$. By Lemma 43 in [1], the minimum eigenvalue of these subspaces is
$$\lambda_0(H|_{\mathcal{S}_1}) \geq 1. \tag{D.3}$$

2. **Evolve-to-Illegal Subspace**, $\mathcal{S}_2$: All standard basis states $|x\rangle \in \mathcal{S}_2 \subset \mathfrak{B}^{\otimes L}$ will evolve either forwards or backwards in time to an illegal state in $O(L^2)$ steps under the transition rules. As per Lemma 5.8 of [16], the minimum eigenvalue of these subspaces is
$$\lambda_0(H|_{\mathcal{S}_2}) = \Omega(L^{-2}). \tag{D.4}$$

3. **Legal Subspace**, $\mathcal{S}_3$: all standard basis states in $\mathcal{S}_3$ are legal and *do not* evolve to illegal states. By Lemma 43 in [1], they have zero support on H_{pen} or H_{in}.

In our renormalisation procedure we seek to preserve only the low energy subspace, hence at any point where we can locally identify states as being in subspace $\mathcal{S}_1$ or $\mathcal{S}_2$, we will remove them from the state space in the renormalisation step.

However, we note that in the general case we cannot locally identify all states in $\mathcal{S}_2$. That is, determining the whether a state evolves to an illegal state under the action of the transitions may be impossible in general if we only look at what the state looks like on a $O(1)$-subset of the sites.

D.2.1 The Ground States

From [1] we know that there are two cases we need to consider depending on whether the QTM encoded in $H_q(L)$ halts or does not halt.

Lemma 19. *Let a given UTM be encoded in the Gottesman-Irani Hamiltonian $H_q(L)$. Then $H_q(L)$ has a ground state energy that is either 0 if the UTM does not halt within time $T(L)$ or $1-\cos(\frac{\pi}{2T})$ if the UTM does halt within $T(L)$. $T(L)$ is a fixed, predetermined function. In the non-halting case, the ground state is*

$$|\Psi_{hist}(L)\rangle = \frac{1}{\sqrt{T}} \sum_{t=1}^{T(L)} |t\rangle |\psi_t\rangle , \tag{D.5}$$

and in the halting case it is

$$|\Psi_{halt}(L)\rangle = \sum_{t=1}^{T(L)} 2\cos\left(\frac{(2t+1)\pi t}{4T}\right) \sin\left(\frac{\pi}{4T}\right) |t\rangle |\psi_t\rangle , \tag{D.6}$$

where $|t\rangle$ is the state of the clock register and $|\psi_t\rangle = \prod_{j=1}^{t} U_j |\psi_0\rangle$ and $|\psi_0\rangle$ is the initial state of the computational register and the $\{U_t\}$ represent the action of the QTM at time step t.

Proof. Combine the standard form property of H_q from [1] with Lemma 5.10 of [16]. □

D.3 Block Renormalisation of the Gottesman-Irani Hamiltonian

In this section we will construct a renormalisation scheme for the Gottesman-Irani Hamiltonian. For a given spin at site i, we write each possible conventional basis state (i.e. basis state before the RG procedure has started) as $\left|\begin{bmatrix} a \\ \alpha \end{bmatrix}\right\rangle_{(i)} \in \mathbb{C}^C \otimes \mathbb{C}^Q$, where the top cell indicates the classical tracks of the construction encoded in [1], while the bottom cell indicates the quantum tracks (see Subsection D.1).

We then define a pair of operations: the blocking operation $\mathcal{B}_q$ and the truncation operation $\mathcal{T}_q$. Given a line of qudits $\mathcal{B}_q$ will essentially combine two lattice sites into a single site with a larger local Hilbert space dimension, while $\mathcal{T}_q$ will remove any of the new single site states which can be locally

detected to have non-zero overlap with the ground state. Thus $\mathcal{T}_q$ reduces the local Hilbert space dimension.

We note that we do not truncate all high energy states since in the halting case this would remove the ground state of the Gottesman-Irani Hamiltonian. Instead, we removed states based on a combination of high energy and a priori knowledge of the ground state.

Blocking $\mathcal{B}_q$

The blocking part of the renormalisation procedure is defined as follows.

Definition 20 (Gottesman-Irani Blocking, $\mathcal{B}_q$)**.** *Let $|\psi\rangle \in \mathcal{H}_q^{(i)} \otimes \mathcal{H}_q^{(i+1)}$, $i \in \mathbb{N}$. The blocking operation, $\mathcal{B}_q : \mathcal{H}_q^{(i)} \times \mathcal{H}_q^{(i+1)} \to \mathcal{H}_q'^{(i/2)}$, is given by the action of the unitary $U_{i,i+1} : \mathcal{H}_q^{(i)} \times \mathcal{H}_q^{(i+1)} \to R(\mathcal{H}_q)'$ as*

$$\mathcal{B}_q^{(i,i+1)} : |\psi\rangle \mapsto U_{i,i+1} |\psi\rangle$$

where

$$U_{i,i+1} = \sum_{|x\rangle, |y\rangle \in \mathfrak{B}} |xy\rangle_{i/2} \langle x|_i \langle y|_{i+1} .$$

We extend this to $|\chi\rangle \in \mathcal{H}_q^{\otimes L}$ as

$$\mathcal{B}_q : |\chi\rangle \mapsto U |\chi\rangle ,$$

where $U = \bigotimes_{i \in 2\mathbb{N}}^{i \leq L/2} U_{i,i+1}$.

This can be expressed more intuitively in terms of basis states:

$$\mathcal{B}_q^{(i,i+1)} : \left| \begin{array}{|c|} \hline a \\ \hline \alpha \\ \hline \end{array} \right\rangle_{(i)} \otimes \left| \begin{array}{|c|} \hline b \\ \hline \beta \\ \hline \end{array} \right\rangle_{(i+1)} \longrightarrow \left| \begin{array}{|c|c|} \hline a & b \\ \hline \alpha & \beta \\ \hline \end{array} \right\rangle_{(i/2)} .$$

Here we have assumed i is odd. Note that $\mathcal{B}_q$ is just a relabelling of the space, so the local Hilbert space dimension is now $\mathbb{C}^{d^2}$ and part of the tensor product structure is lost. We denote by $\mathcal{H}_q'$ this new local Hilbert space spanned by the basis $\mathfrak{B}'^{(1)}$.

Truncation $\mathcal{T}_q$

The truncation part of the RG map truncates the local Hilbert space to discard those states which locally have support on the penalty terms.

Definition 21 (Gottesman-Irani Truncation Mapping, $\mathcal{T}_q$). *Let $\mathfrak{B}^{(1)}$ be the set of basis states defined by $\mathcal{B}_q$ such states with a preimage $|a\rangle|b\rangle$, such that $|a\rangle, |b\rangle \in \mathfrak{B}$ cannot be locally identified as being in subspace $\mathcal{S}_1$ or $\mathcal{S}_2$. That is*

$$\langle a| \langle b| h_{pen}^{i,i+1} |a\rangle |b\rangle = \langle a| \langle b| h_{in}^{i,i+1} |a\rangle |b\rangle = 0, \tag{D.7}$$

$$\langle a| \langle b| h_{trans}^{(i,i+1)} h_{pen}^{(i,i+1)} h_{trans}^{(i,i+1)} |a\rangle |b\rangle = 0. \tag{D.8}$$

The truncation mapping is then $\mathcal{T}_q^{(i,i+1)} : R(\mathcal{H}_q)' \to R(\mathcal{H}_q)$ for $R(\mathcal{H}_q) = \mathrm{span}\{\mathfrak{B}_q^{(1)}\} \subset R(\mathcal{H})'_q$. Then the full restriction is $\mathcal{T}_q : \mathcal{H}_q'^{\otimes L/2} \to R(\mathcal{H}_q)^{\otimes L/2}$.

We now combine the unitary and subspace restriction to give an isometry which implements $\mathcal{T}_q \circ \mathcal{B}_q$.

Lemma 22 (Renormalisation Unitary Structure). *Let the renormalisation isometry $V_{i,i+1}^{GI}$ be the unitary map follow by subspace restriction previously described. Define $V^{GI} : \mathcal{H}_q^{\otimes L} \to R(\mathcal{H}_q)^{\otimes L/2}$ to implement the mapping $\mathcal{T}_q \circ \mathcal{B}_q$ on a state in $\mathcal{H}_q^{\otimes L}$, as*

$$\mathcal{T}_q \circ \mathcal{B}_q : |\chi\rangle \mapsto U |\chi\rangle |_{R(\mathcal{H}_q)^{\otimes L/2}} =: V^{GI} |\chi\rangle . \tag{D.9}$$

where U is defined in Definition 20 and $R(\mathcal{H}_q)$ is defined in Definition 21. Then V^{GI} can be defined as and decomposed as

$$V^{GI} := \bigotimes_{i \in 2\mathbb{N}}^{i \le \lfloor L/2 \rfloor} V_{i,i+1}^{GI} = \bigotimes_{i \in 2\mathbb{N}}^{i \le \lfloor L/2 \rfloor} \left(\bigotimes_{j=1}^{6} V_{i,i+1}^{GI\ (j)} \right), \tag{D.10}$$

with

$$V_{i,i+1}^{GI} : \mathcal{H}_q^{\otimes 2} \to R(\mathcal{H}_q) \tag{D.11}$$

and where each part of the decomposition acts on one of the six different tracks,

$$V_{i,i+1}^{GI\ (j)} : \mathcal{H}_{q,j}^{\otimes 2} \to R(\mathcal{H}_q)_j . \tag{D.12}$$

Proof. The tensor structure $V^{GI} = \bigotimes_{i \in 2\mathbb{N}}^{i \le \lfloor L/2 \rfloor} V_{i,i+1}^{GI}$ is evident from the block procedure. The decomposition $V_{i,i+1}^{GI} = \bigotimes_{j=1}^{6} V_{i,i+1}^{GI\ (j)}$ arises from the fact

that the procedure keeps each basis state as a product across the different tracks and hence the different $\mathcal{H}_{q,j}$. $\square$

We now need to define how the Hamiltonian acts with respect to the RG procedure. We want to break down the Hamiltonian into different subspaces and renormalise them separately while preserving the ground state (in both the halting and non-halting cases) and its energy.

We introduce the following lemma which will essentially define the way the RG scheme acts on the quantum part of the Hilbert space, and thus forms an key part of what will be our overall RG scheme.

Lemma 23 (Renormalised Gottesman-Irani Hamiltonian)**.** *Let h_q be the local terms of a nearest neighbour, translationally invariant Hamiltonian*

$$H_q(L) = \sum_{i=1}^{L} h_q^{(i,i+1)} = H_{trans} + H_{pen} + H_{in} + H_{out}, \tag{D.13}$$

such that $H(L)$ is standard form. Let $V^{GI} : \mathbb{C}^d \otimes \mathbb{C}^d \to \mathbb{C}^{f(d)}$, be the isometry from Lemma 22. Then the renormalised Hamiltonian, defined as

$$\mathcal{R}(H_q(2L)) = V^{GI} H_q(2L) V^{GI\dagger} = \sum_{i=1}^{L/2} V^{GI} h_q^{(i,i+1)} V^{GI\dagger} = R(H_q)(L), \tag{D.14}$$

is a translationally invariant, nearest-neighbour Hamiltonian with local interactions $R(h_q)^{(i/2,i/2+1)} = V^{GI}(h_q^{(i-1,i)} + h_q^{(i+1,i+2)})V^{GI\dagger}$ and $R(h_q)^{i/2} = V^{GI} h_q^{(i,i+1)} V^{GI\dagger}$. Furthermore, $R(H_q)(L)$ has the following properties:

1. *$R(H_q)(L)$ is a standard form Hamiltonian.*

2. *$R(H_{trans})$ encodes a transition $V^{GI}(|ab\rangle |\psi_{abcd}\rangle) \to V^{GI}(|cd\rangle U_{abcd} |\psi_{abcd}\rangle)$ iff H_{trans} encodes the transition $|ab\rangle |\psi_{abcd}\rangle \to |cd\rangle U_{abcd} |\psi_{abcd}\rangle$.*

3. *$R(H_{pen}), R(H_{in}), R(H_{out})$ have support on a renormalised basis state $V^{GI}(|ab\rangle |\psi\rangle)$ iff H_{pen}, H_{in}, H_{out} respectively have non-zero support on $|ab\rangle |\psi\rangle$.*

4. *$\lambda_0(H_q(L)) = \lambda_0(R(H_q)(L/2))$ (the ground state energy is preserved).*

5. *$R(H_q)$ maintains the six-fold tensor product structure of the original Hamiltonian H_q in Subsection D.1, that is, $R(\mathcal{H}_q) = \bigotimes_{j=1}^{6} R(\mathcal{H}_q)_j$.*

Proof. First note that for all $i \in 2\mathbb{N}$, $V^{GI}_{i,i+1} h^{(i,i+1)} V^{GI\dagger}_{i,i+1} \in \mathcal{B}(\mathbb{C}^{f(d)})$ is now a 1-local term in the new renormalised Hamiltonian. However,

$$V^{GI}_{i+2,i+3} V^{GI}_{i,i+1} h^{(i+1,i+2)} V^{GI\dagger}_{i,i+1} V^{GI\dagger}_{i+2,i+3} \in \mathcal{B}(\mathbb{C}^{f(d)} \otimes \mathbb{C}^{f(d)})$$

is 2-local.

Claims 1 and 2

From the linearity of V^{GI}, we see that $\mathcal{R}(H_q(L)) = R(H_{trans}) + R(H_{pen}) + R(H_{in}) + R(H_{out})$. It is straightforward to see that $R(H_{trans}) = V^{GI} H_{trans} V^{GI\dagger} = \sum_{ab\to cd} (V^{GI} |cd\rangle \otimes U_{abcd} - V^{GI} |ab\rangle)(\langle cd| \otimes U^{\dagger}_{abcd} V^{GI\dagger} - \langle ab| V^{GI\dagger})$, and hence encodes transitions between the renormalised states. This also shows $R(H_{trans})$ satisfies Claim 2. Due to the properties of decomposition of V^{GI}, as shown in Lemma 22, we preserve that H_{trans} acts diagonally on the states in $\mathbb{C}^C$. Likewise, it preserves the form of H_{pen}, H_{in}, H_{out} as projectors onto a subset of states.

Claim 3: Consider the penalty terms: given a renormalised state $V^{GI} |\psi\rangle$, it is clear that

$$(\langle\psi| V^{GI\dagger}) V^{GI} H_{pen} V^{GI\dagger} (V^{GI} |\psi\rangle) = \langle\psi| H_{pen} |\psi\rangle = 1,$$

hence $V^{GI} |\psi\rangle$ is penalised by the renormalised Hamiltonian iff $|\psi\rangle$ is penalised by the unrenormalised Hamiltonian. The same applied to H_{in} and H_{out}.

Claim 4: First note that any state

$$|\Psi\{a_t\}\rangle = \sum_{t=1}^{T} a_t (|t\rangle |\psi_t\rangle). \tag{D.15}$$

which encodes a valid evolution is in the kernel of H_{in}, H_{pen}, and is contained in subspace $\mathcal{S}_3$. Thus, $V^{GI} |\Psi\{a_t\}\rangle \in R(\mathcal{H})^{\otimes L/2}$, and after the RG procedure $\mathcal{T}_q \circ \mathcal{B}_q$ the corresponding renormalised state is

$$|\Psi'\{a_t\}\rangle = \sum_{t=1}^{T} a_t V^{GI} (|t\rangle |\psi_t\rangle). \tag{D.16}$$

To see the energy of such states is preserved, note that

$$\left\langle \Psi'\{a_t\}\right| V^{GI} H_q(L) V^{GI\dagger} \left|\Psi'\{a_t\}\right\rangle = \langle \Psi\{a_t\}| H_q(L) |\Psi\{a_t\}\rangle . \tag{D.17}$$

From Lemma 19 the ground states are of the form $|\Psi\{a_t\}\rangle$. We know that the state $V^{GI}|\Psi\{a_t\}\rangle$ has the same energy. Since the minimum eigenvalue is given by

$$\lambda_0(H_q(L)) = \min_{|x\rangle \in \mathcal{H}_q^{\otimes L}} \frac{\langle x| H_q(L) |x\rangle}{\langle x|x\rangle} \tag{D.18}$$

$$= \min_{|x\rangle \in \mathcal{H}_q^{\otimes L}} \frac{\langle x| UU^\dagger H_q(L) U^\dagger U |x\rangle}{\langle x| U^\dagger U |x\rangle} \tag{D.19}$$

$$\leq \min_{\substack{|x\rangle \in \mathcal{H}_q^{\otimes L} \\ V^{GI}|x\rangle \neq 0}} \frac{\langle x| V^{GI} V^{GI\dagger} H_q(L) V^{GI\dagger} V^{GI} |x\rangle}{\langle x| V^{GI\dagger} V^{GI} |x\rangle} \tag{D.20}$$

$$= \lambda_0(R(H_q)(L/2)), \tag{D.21}$$

where going from eq. (D.19) to eq. (D.20) we have used the fact that we have restricted the subspace to remove the states that are integrated out by V^{GI}. Since $\lambda_0(R(H_q)(L/2)) = \lambda_0(H_q(L/2))$, then we can confirm $V^{GI}|\psi_{halt}\rangle$ and $V^{GI}|\psi_{hist}\rangle$ are the appropriate ground states after the renormalisation procedure.

Claim 5: The preservation of the six-fold tensor product structure in Subsection D.1 follows directly from the tensor product form of the isometry given in eq. (D.10) applied according to the renormalisation method described by eq. (D.14).

□

D.4 Multiple Iterations

Consecutive steps of the RG procedure can be derived straightforwardly. The Hilbert space obtained after k-th RG steps of can be constructed by induction

$$(\mathcal{T}_q \circ \mathcal{B}_q)^{\circ(k)} = \mathcal{T}_q \circ \mathcal{B}_q \circ (\mathcal{T}_q \circ \mathcal{B}_q)^{\circ(k-1)}. \tag{D.22}$$

We first combine two basis elements in the space $\mathfrak{B}^{(k-1)}$ into a new state, i.e.,

$$\left|\begin{array}{|c|c|c|c|}\hline a_1 & a_2 & \cdots & a_{2(k-1)} \\ \hline \alpha_1 & \alpha_2 & \cdots & \alpha_{2(k-1)} \\ \hline\end{array}\right\rangle \otimes \left|\begin{array}{|c|c|c|c|}\hline b_1 & b_2 & \cdots & b_{2(k-1)} \\ \hline \beta_1 & \beta_2 & \cdots & \beta_{2(k-1)} \\ \hline\end{array}\right\rangle = \left|\begin{array}{|c|c|c|c|c|c|c|c|}\hline a_1 & a_2 & \cdots & a_{2(k-1)} & b_1 & b_2 & \cdots & b_{2(k-1)} \\ \hline \alpha_1 & \alpha_2 & \cdots & \alpha_{2(k-1)} & \beta_1 & \beta_2 & \cdots & \beta_{2(k-1)} \\ \hline\end{array}\right\rangle$$

We then truncate the basis set according to the criteria described in the previous section. This will generate the set of renormalised local basis states $\mathfrak{B}^{(k)}$. The local Hilbert space after k RG iterations is denoted by $R^{(k)}(\mathcal{H})$. We note that this can still be decomposed it as $R^{(k)}(\mathcal{H}) = \bigotimes_{i=1}^{6} R^{(k)}(\mathcal{H})_i$ corresponding to the 6 tracks of the original construction.

We can thus concatenate multiple renormalisations of the Gottesman-Irani Hamiltonian in one isometry, $V^{GI}(k) : R^{(k-1)}(\mathcal{H}_q)^{\otimes 2L} \to R^{(k)}(\mathcal{H}_q)^{\otimes L}$, given by

$$V^{GI}[k] = \prod_{j=1}^{k} V^{GI}_{L/2^j} \tag{D.23}$$

where $V^{GI}_{L/2^j}$ is the isometry outlined in Lemma 22, but now acting on the appropriate local Hilbert space, and the subscript $L/2^j$ indicates that the operator is acting on a 1D chain of $L/2^j$ sites. We use square brackets $[,]$ to emphasise that $[k]$ applies to the number of renormalisation steps that have been performed, not any spatial position or lattice site Accordingly, the renormalised Hamiltonian is then

$$R^{(k)}(H_q(L)) = V^{GI}[k] H_q(L) V^{GI\dagger}[k]. \tag{D.24}$$

It follows immediately from Lemma 23 is that this RG mapping takes standard form Hamiltonians to standard form Hamiltonians while preserving the energy of the ground state. Thus we have

Corollary 24. *Multiple iterations of the RG map applied to $H_q(L)$ preserve the properties (1-5) in Lemma 23.*

E Putting it all Together

In this section we combine the renormalisation group schemes for the separate parts of the Hamiltonian. First recall Lemma 51 of [1] which characterises the ground state of the Hamiltonian defined by the local terms h_u:

Lemma 25 (Tiling + quantum layers, Lemma 51 of [1]). *Let $h_c^{\text{row}}, h_c^{\text{col}} \in \mathcal{B}(\mathbb{C}^C \otimes \mathbb{C}^C)$ be the local interactions of a 2D tiling Hamiltonian H_c, with two distinguished states (tiles) $|L\rangle, |R\rangle \in \mathbb{C}^C$. Let $h_q \in \mathcal{B}(\mathbb{C}^Q \otimes \mathbb{C}^Q)$ be the local interaction of a Gottesman-Irani Hamiltonian $H_q(r)$, as in Section D. Then there is a Hamiltonian on a 2D square lattice with nearest-neighbour interactions $h_u^{\text{row}}, h_u^{\text{col}} \in \mathcal{B}(\mathbb{C}^{C+Q+1} \otimes \mathbb{C}^{C+Q+1})$ with the following properties: For any region of the lattice, the restriction of the Hamiltonian to that region has an eigenbasis of the form $|T\rangle_c \otimes |\psi\rangle_q$, where $|T\rangle_c$ is a* product *state representing a classical configuration of tiles. Furthermore, for any given $|T\rangle_c$, the lowest energy choice for $|\psi\rangle_q$ consists of ground states of $H_q(r)$ on segments between sites in which $|T\rangle_q$ contains an $|L\rangle$ and an $|R\rangle$, a 0-energy eigenstate on segments between an $|L\rangle$ or $|R\rangle$ and the boundary of the region, and $|e\rangle$'s everywhere else.*

The $|L\rangle$ and $|R\rangle$ tiles are identified in [1] with the right-down and left-down cross with red markings in the Robinson tiles respectively (see Supplementary Fig. 1 in Section C). Importantly, these are the tiles which appear at the top-left and top-right of Robinson squares in the Robinson tiling. The ground state can then be shown to be the ground state of the Robinson tiling Hamiltonian plus a "quantum layer" in which the Gottesman-Irani ground states appear only over the tops of the Robinson squares. Everywhere else in the quantum layer is a filler state $|e\rangle$.

A key point is that the eigenstates are all product states across $\mathcal{H}_c$ and $\mathcal{H}_{eq}$. We wish for the RG mapping to preserve this property. This restricts the type of renormalisation isometries we use, as detailed in the following lemma.

Lemma 26 (Separable Eigenstates). *Let $H_u^{\Lambda(2L)}$ denote the Hamiltonian in Lemma 25. Then for an isometry $Z = Z_c \otimes Z_{eq}$ where $Z_c : \mathcal{H}_c^{\otimes 2\times 2} \to R(\mathcal{H}_c)$ and $Z_{eq} : \mathcal{H}_{eq}^{\otimes 2\times 2} \to R(\mathcal{H}_{eq})$, the operator $ZH_u^{\Lambda(2L)}Z^\dagger$ also has eigenstates of the form $|T'\rangle_c \otimes |\psi\rangle_{eq}$ for $|T'\rangle_c \in R(\mathcal{H}_c)^{\otimes \Lambda(L)}$ and $|\psi\rangle_{eq} \in R(\mathcal{H}_{eq})^{\otimes \Lambda(L)}$.*

Proof. As per Lemma 25, the eigenstates of $H_u^{\Lambda(2L)}$ decompose as product states $|T_c\rangle \otimes |\psi_i\rangle_{eq}$, hence we can write

$$H_u^{\Lambda(2L)} = \sum_i \lambda_i |T_i\rangle\langle T_i| \otimes |\psi_i\rangle\langle\psi_i|_{eq} . \tag{E.1}$$

Applying the renormalisation isometry Z gives

$$ZH_u^{\Lambda(2L)}Z^\dagger = \sum_i \lambda_i Z_C \left|T_i\middle\rangle\!\middle\langle T_i\right|_c Z_C^\dagger \otimes Z_{eq} \left|\psi_i\middle\rangle\!\middle\langle\psi_i\right|_{eq} Z_{eq}^\dagger$$
$$=: \sum_i \lambda_i \left|T_i'\middle\rangle\!\middle\langle T_i'\right|_{c'} \otimes \left|\psi_i'\middle\rangle\!\middle\langle\psi_i'\right|_{eq'}.$$

Thus the product structure across the two subspaces is preserved. □

In Corollary 8 we showed that the ground state of the renormalised tiling Hamiltonian preserves the tiling pattern of the unrenormalised Hamiltonian. Here we show that renormalising the full Hamiltonian preserves this Robinson tiling plus Gottesman-Irani ground state structure.

We start by considering how to renormalise the Gottesman-Irani Hamiltonian in the presence of filler states on a 2D lattice (as opposed to the 1D chain considered previously). After this we show the ground state energy Hamiltonian is preserved under the RG map.

E.1 Renormalising $\mathcal{H}_T \otimes (\mathcal{H}_e \oplus \mathcal{H}_q)$

From Lemma 26, we know the eigenstates of the Hamiltonian defined by h_u are product states across the classical-quantum Hilbert space partition and this structure is preserved under a tensor product of isometries on the two subspace separately. Thus we can consider the basis states of $\mathcal{H}_T$ and $\mathcal{H}_{eq}$ separately, develop RG schemes for them separately, and then show the separability property preserves the desired properties.

Blocking Operation $\mathcal{B}_u$ We know that V^C from Lemma 12 will renormalise the classical state space by mapping sets of 2×2 tiles to new tiles which recreate the tiling pattern at all but the lowest level. We use this isometry unaltered, acting on the classical part of the Hilbert space.

Consider first the quantum Hilbert space $\mathcal{H}_{eq}$ and recall that the Gottesman-Irani Hamiltonian to be renormalised is a standard form Hamiltonian, and so can be renormalised as per Subsection D.3. However, the blocking procedure from Subsection D.3 is not sufficient for our purposes as it (a) takes a set of 2×1 lattice sites to a single lattice site and so is not appropriate for a 2D lattice, and (b) does not include the filler state $|e\rangle_e$. To remedy this we

need an isometry which acts as:

$$V^{eq}_{(i,i+1)(j,j+1)} : \mathcal{H}^{(i,j)}_{eq} \otimes \mathcal{H}^{(i+1,j)}_{eq} \otimes \mathcal{H}^{(i,j+1)}_{eq} \otimes \mathcal{H}^{(i+1,j+1)}_{eq} \to (\mathcal{H}'_{eq} \otimes \mathcal{H}'_{eq})^{(i/2,j/2)}. \tag{E.2}$$

We will find it useful to define the following notation:

Definition 27 (k-times Blocked Basis States)**.** *Let* $|x_1\rangle, |x_2\rangle, \ldots, |x_{2^k}\rangle \in \mathfrak{B} \cup |e\rangle_e$*, then we denote the corresponding renormalised basis state after* k *applications of the RG mapping as* $|x_1 x_2 \ldots x_{2^k}\rangle$*.*

Now define $V^q_{(i,i+1)}(j)$ as follows, where $V^{GI}_{i,i+1}$ is the isometry used in Lemma 23:

$$V^q_{(i,i+1)}(j) = V^{GI}_{i,i+1} + |ee\rangle_{i/2,j/2} \langle e|_{i,j} \langle e|_{i+1,j} \tag{E.3}$$

$$+ |xe\rangle_{i/2,j/2} \langle x|_{i,j} \langle e|_{i+1,j} + |ex\rangle_{i/2,j/2} \langle e|_{i,j} \langle x|_{i+1,j} . \tag{E.4}$$

This defines a new set of quantum basis states which now reflect the fact $|e\rangle_e$ is part of the Hilbert space. Denote this

$$\mathfrak{C}^{(1)} := \mathfrak{B}^{(1)} \cup |ee\rangle \bigcup_{x\in\mathfrak{B}} |ex\rangle \bigcup_{x\in\mathfrak{B}} |xe\rangle . \tag{E.5}$$

These isometries essentially apply the same mapping as V^{GI}, but now account for the additional $|e\rangle_e$ state we have introduced. However, V^q only maps 2×1 spins to a single spin. We need an operator which maps a 2×2 spin to a single spin. Define $W : \mathcal{H}'^{(i/2,j)}_{eq} \otimes \mathcal{H}'^{(i/2,j+1)}_{eq} \to (\mathcal{H}'_{eq} \otimes \mathcal{H}'_{eq})^{(i/2,j/2)}$, as simply

$$W_{(i,i+1)(j,j+1)} = \sum (|x\rangle_{q_1} \otimes |y\rangle_{q_2})_{i/2,j/2} \langle x|_{i/2,j} \otimes \langle y|_{i/2,j+1} . \tag{E.6}$$

This unitary acts to map the 1×2 set of sites to a single lattice site in the renormalised lattice. The isometry:

$$V^{eq}_{(i,i+1)(j,j+1)} := W_{(i,i+1)(j,j+1)} \left(V^q_{(i,i+1)}(j) \otimes V^q_{(i,i+1)}(j+1) \right), \tag{E.7}$$

then maps 2×2 spins to a single spin. The overall blocking map $\mathcal{B}_u$ is then given by:

Definition 28 (Blocking Isometry, V^b, $\mathcal{B}_u$)**.** *Let V^C and V^{eq} be the isometries from Definition 11 and eq. (E.7) respectively. Then the blocking isometry for H_u is given by*

$$V^b_{(i,i+1)(j,j+1)} = V^C_{(i,i+1)(j,j+1)} \otimes V^{eq}_{(i,i+1)(j,j+1)}. \tag{E.8}$$

We now need to consider the full renormalisation process: the isometry defined above will map a certain subset of states to states on the renormalised lattice. However, some parts of the Hilbert space will be "integrated out". For convenience we will sometimes use indices I, J to indicate row and column indices on the new lattice after the RG transformation.

Let $h_q^{(i,i+1)}(j), h_q^{(i,i+1)}(j+1)$ be the local terms of the quantum Hamiltonian before renormalisation, then we see that:

$$\begin{aligned}
&V^{eq}_{(i,i+1)(j,j+1)} \left(h_q^{(i,i+1)}(j+1) + h_q^{(i,i+1)}(j) \right) V^{eq\dagger}_{(i,i+1)(j,j+1)} \\
&= h_q^{(1)\prime(I,J)} \otimes \mathbb{1}_{q_2} + \mathbb{1}_{q_1} \otimes h_q^{(1)\prime(I,J)}
\end{aligned}$$

and

$$\begin{aligned}
&V^{eq}_{(i+2,i+3)(j,j+1)} V^{eq}_{(i,i+1)(j,j+1)} \left(h_q^{(i+1,i+2)}(j) + h_q^{(i+1,i+2)}(j) \right) V^{eq\dagger}_{(i+2,i+3)(j,j+1)} \\
&\times V^{eq\dagger}_{(i,i+1)(j,j+1)} = h'^{(I,I+1)}_{q_1}(J) \otimes \mathbb{1}^{(I,J)}_{q_2} \otimes \mathbb{1}^{(I+1,J)}_{q_2} + \mathbb{1}^{(I,J)}_{q_1} \otimes \mathbb{1}^{(I+1,J)}_{q_1} \otimes h'^{(I,I+1)}_{q_2}(J).
\end{aligned}$$

Truncation Operation $\mathcal{T}_u$ The operator W has essentially merged two sites into a single site. We now wish to integrate out one of these parts of the Hilbert space and restrict to the set of "allowed states" in the other. We will implement this using the 1-local projector $\Pi_{gs}(k)$

Definition 29 (Truncation Operation $\mathcal{T}_u$)**.** *Let $|\psi\rangle \in \mathcal{H}_c \otimes \mathcal{H}_{eq}$, then*

$$\mathcal{T}_u : |\psi\rangle \mapsto (\mathbb{1}_c \otimes \mathbb{1}_{q_1} \otimes \Pi_{gs}(k)) \, |\psi\rangle \,, \tag{E.9}$$

where

$$\Pi_{gs}(k) = \begin{cases} \left|e^{\times 2^k}\right\rangle\!\left\langle e^{\times 2^k}\right| & k \text{ even} \\ \left|\psi_{hist}(4^n+1)e^{\times 2^k-4^n-1}\right\rangle\!\left\langle \psi_{hist}(4^n+1)e^{\times 2^k-4^n-1}\right| & \text{if } k \text{ odd, } 2^{k-1} < 4^n+1 < 2^k, \\ & \text{and non-halting} \\ \left|\psi_{halt}(4^n+1)e^{\times 2^k-4^n-1}\right\rangle\!\left\langle \psi_{halt}(4^n+1)e^{\times 2^k-4^n-1}\right| & \text{if } k \text{ odd, } 2^{k-1} < 4^n+1 < 2^k, \\ & \text{and halting,} \end{cases} \tag{E.10}$$

and where $|\psi_{hist}(L)\rangle$ and $|\psi_{halt}(L)\rangle$ are defined in Lemma 19. This extends to states $|\chi\rangle \in (\mathcal{H}_c \otimes \mathcal{H}_{eq})^{\otimes \Lambda(L)}$, as

$$\mathcal{T}_u : |\chi\rangle \mapsto \bigotimes_{(I,J)\in\Lambda(L)} (\mathbb{1}_c^{(I,J)} \otimes \mathbb{1}_{q1}^{(I,J)} \otimes \Pi_{gs}^{(I,J)}(k)) \, |\chi\rangle \, . \tag{E.11}$$

Definition 30 (Renormalisation Isometry, V^u)**.** *Let $V^b_{(i,i+1)(j,j+1)}$ and Π_{gs} be as defined in Definition 28 and eq. (E.10) respectively. We define the isometry implementing the entire renormalisation scheme on H_u as*

$$V^u_{(i,i+1)(j,j+1)} := (\mathbb{1}_c \otimes \Pi_{gs}) V^b_{(i,i+1)(j,j+1)}. \tag{E.12}$$

To see why this is appropriate note that the Hamiltonian after the application of the blocking isometries has two sets of local terms: a 1-local term and a 2-local term (see Definition 31 and the discussion following). First consider the 1-local term $h_q^{(1)\prime(I,J)} \otimes \mathbb{1}_{q_2} + \mathbb{1}_{q_1} \otimes h_q^{(1)\prime(I,J)}$ and examine how it transforms under $\mathcal{T}_u$ and Π_{gs}. The idea is that Π_{gs} will "integrate out" the q_2 subspace by removing all states which are not the ground state while maintaining the energy contribution from this subspace. If the site is large enough to contain a full history state of length 4^n+1, for some $n \in \mathbb{N}$, then we keep only that state and the relevant renormalised $|e\rangle$ states. Otherwise we keep only the renormalised $|e\rangle$ states. Hence

$$\Pi_{gs}^{(I,J)}(k)(h_{q_1}^{(1)\prime(I,J)} \otimes \mathbb{1}_{q_2}^{(I,J)} + \mathbb{1}_{q_1}^{(I,J)} \otimes h_{q_2}^{(1)\prime(I,J)}) \Pi_{gs}^{(I,J)}(k) \tag{E.13}$$
$$= h_{q_1}^{(1)\prime(I,J)} \otimes \Pi_{gs}^{(I,J)}(k) + \operatorname{tr}\left(\Pi_{gs}^{(I,J)}(k) h_{q_2}^{\prime(I,J)}\right) \mathbb{1}_{q_1}^{(I,J)} \otimes \Pi_{gs}^{(I,J)}(k). \tag{E.14}$$

Since Π_{gs} is a projector onto a 1-dimensional subspace, we will often omit

it when writing the Hamiltonian. Thus we obtain the term

$$h_q^{(1)\prime(I,J)} + \mathrm{Tr}\left(\Pi_{gs}(k) h_{q2}^{\prime(I,J)}\right) \mathbb{1}_q. \tag{E.15}$$

Now examine how the 2-local terms transform:

$$\Pi_{gs}(k)^{(I,J)} \otimes \Pi_{gs}(k)^{(I+1,J)} \big(h_q^{\prime(I,I+1)} \otimes \mathbb{1}_{q_2}^{(I,J)} \otimes \mathbb{1}_{q_2}^{(I+1,J)} \tag{E.16}$$
$$+ \mathbb{1}_{q_1}^{(I,J)} \otimes \mathbb{1}_{q_1}^{(I+1,J)} \otimes h_q^{\prime(I,I+1)}\big) \Pi_{gs}(k)^{(I,J)} \otimes \Pi_{gs}(k)^{(I+1,J)} \tag{E.17}$$
$$= h_q^{\prime(I,I+1)} \otimes \Pi_{gs}(k)^{(I)} \otimes \Pi_{gs}(k)^{(I+1)} \tag{E.18}$$
$$+ \mathrm{tr}\left(h_q^{\prime(I,I+1)} \Pi_{gs}(k)^{(I)} \otimes \Pi_{gs}(k)^{(I+1)}\right) \mathbb{1}_{q_1}^{(I,J)} \otimes \Pi_{gs}(k)^{(I)} \otimes \Pi_{gs}(k)^{(I+1)}. \tag{E.19}$$

Importantly $\mathrm{tr}\left(h_q^{\prime(I,I+1)} \Pi_{gs}(k)^{(I)} \otimes \Pi_{gs}(k)^{(I+1)}\right)$ only picks up a non-zero contribution from the terms proportional to $\mathbb{1}^{(I)} \otimes \mathbb{1}^{(I+1)}$ (we also note that this latter term is zero for interactions going along columns). Again the subspace spanned by $\Pi_{gs}(k)^{(I)} \otimes \Pi_{gs}(k)^{(I+1)}$ is a 1-dimensional subspace and hence we will often omit writing it explicitly. Thus the 2-local terms effectively become $h_q^{\prime(I,I+1)} + \mathrm{tr}\left(h_q^{\prime(I,I+1)} \Pi_{gs}(k)^{(I)} \otimes \Pi_{gs}(k)^{(I+1)}\right) \mathbb{1}_q^{(I,J)} \otimes \mathbb{1}_q^{(I+1,J)}$.

Multiple Iterations The above is the RG transformation for a single iteration; in the following we construct the further iterations of the RG mapping analogously to the above.

First define the set of local basis states in the quantum part of the Hilbert space,

$$\mathfrak{C}^{\prime(k)} := \mathfrak{B}^{(k)} \bigcup_{j=0^{2^K}} \bigcup_{|x_i\rangle \in \mathfrak{B} \cup |e\rangle} |x_1 ... x_{2^k}\rangle \,, \tag{E.20}$$

such that $|x_1 \dots x_{2^{k-1}}\rangle \in \mathfrak{C}^{(k-1)}$. From this we can define $\mathcal{H}_{eq}^{\prime(k)} = \mathrm{span}\left\{|x\rangle \,\middle|\, |x\rangle \in \mathfrak{C}^{\prime(k)}\right\}$.

Then $\mathcal{B}_u : (R^{(k-1)}\mathcal{H}_{eq})^{\otimes 2\times 2} \to \mathcal{H}_{eq}^{\prime(k)}$. Finally we truncate the basis states which are either bracketed or can immediately be identified as being illegal or evolving to an illegal state using $\mathcal{T}_u$. This leaves us with the basis $\mathfrak{C}^{(k)}$ as the set of basis states and the renormalised local quantum Hilbert space as $R^{(k)}(\mathcal{H}_{eq}) = \mathrm{span}\{|x\rangle \,|\, |x\rangle \in \mathfrak{C}^{(k)}\}$.

The $\mathcal{T}_u \circ \mathcal{B}_u$ operation can be implemented analogously to the previously described transformation: we apply V^b — now defined on $R^{(k-1)}(\mathcal{H}_u)$ — across the lattice which blocks and truncates part of the Hilbert space. We then apply $\Pi_{gs}(k)$, as defined in eq. (E.10), to project out the local ground

state (which may pick up energy).

We formalise the overall RG mapping in the following definition:

Definition 31 (h_u Renormalisation Mapping). *Let* $h_u^{col(i,i+1)}, h_u^{row(j,j+1)} \in \mathcal{B}(\mathbb{C}^d \otimes \mathbb{C}^d)$ *and* $V^u_{(i,i+1)(j,j+1)}$ *be as in Definition 30. Then the renormalised local terms are given by*

$$\mathcal{R} : h_u^{row(i+1,i+2)}(j) + h_u^{row(i+1,i+2)}(j+1) \to V^u_{(i+2,i+3)(j,j+1)} V^u_{(i,i+1)(j,j+1)} \times \tag{E.21}$$
$$\left(h_u^{row(i+1,i+2)}(j) + h_u^{row(i+1,i+2)}(j+1)\right) V^{u\dagger}_{(i,i+1)(j,j+1)} V^{u\dagger}_{(i+2,i+3)(j,j+1)} \tag{E.22}$$
$$=: R(h_u^{row})^{(i,i+1)} \tag{E.23}$$
$$\mathcal{R} : h_u^{col(j+1,j+2)}(i) + h_u^{col(j+1,j+2)}(i+1) \to V^u_{(i+2,i+3)(j,j+1)} V^u_{(i,i+1)(j,j+1)} \times \tag{E.24}$$
$$\left(h_u^{col(j+1,j+2)}(i) \ + h_u^{col(j+1,j+2)}(i+1)\right) V^{u\dagger}_{(i,i+1)(j,j+1)} V^{u\dagger}_{(i+2,i+3)(j,j+1)}, \tag{E.25}$$
$$=: R(h_u^{col})^{(i,i+1)} \tag{E.26}$$

$$\mathcal{R} : h_u^{row(i,i+1)}(j) + h_u^{row(i+1,i+2)}(j+1) + \sum_{\substack{k=0,1\\ \ell=1,2}} \left(h_u^{(1)(i+k,j+\ell)}\right) \to \tag{E.27}$$
$$V^u_{(i,i+1)(j,j+1)} \left(h_u^{row(i,i+1)}(j) + h_u^{row(i+1,i+2)}(j+1) + \sum_{\substack{k=0,1\\ \ell=1,2}} \left(h_u^{(1)(i+k,j+\ell)}\right)\right) V^{u\dagger}_{(i,i+1)(j,j+1)} \tag{E.28}$$
$$=: R(h_u^{(1)})^{(i)}. \tag{E.29}$$

$R^{(k)}(h_u^{row}), R^{(k)}(h_u^{col})^{(i,i+1)}, R^{(k)}(h_u^{(1)})^{(i)}$ *are defined in the same way but with the appropriate isometries for the* k^{th} *iteration of the RG mapping.*

Remark 32. $R^{(k)}(h_u^{(1)})^{(i)}$ *and* $R^{(k)}(h_u^{row})^{(i,i+1)}$ *have local projector terms of the form* $\sum_{m=1}^k 4^m \kappa^{(m)} \mathbb{1}^{(i)}$ *and* $\sum_{m=1}^k 2^m \gamma^{(m)} \mathbb{1}^{(i)} \otimes \mathbb{1}^{(i+1)}$*, where* $\gamma^{(k)}$ *and* $\kappa^{(k)}$ *are given by*

$$\kappa^{(k)} := \mathrm{Tr}\left(\Pi_{gs}(k) h'^{(I,J)}_{q_2}\right) \tag{E.30}$$
$$\gamma^{(k)} := \mathrm{tr}\left(h'^{(I,I+1)}_q \Pi_{gs}(k)^{(I)} \otimes \Pi_{gs}(k)^{(I+1)}\right). \tag{E.31}$$

E.2 Properties of the RG Mapping

With the following results we examine how the relevant properties of the full Hamiltonian, and in particular its ground state energy, are preserved under the RG mapping.

Lemma 33 (H_u Renormalisation)**.** *Let $H_u(L) = \sum h_u^{row(j,j+1)} + \sum h_u^{col(i,i+1)}$, where*

$$
\begin{aligned}
h^{\text{col}}_{j,j+1} =& h^{\text{col}}_c \otimes \mathbb{1}^{(j)}_{eq} \otimes \mathbb{1}^{(j+1)}_{eq} && \text{(E.32a)}\\
h^{\text{row}}_{i,i+1} =& h^{\text{row}}_c \otimes \mathbb{1}^{(i)}_{eq} \otimes \mathbb{1}^{(i+1)}_{eq} && \text{(E.32b)}\\
&+ \mathbb{1}^{(i)}_c \otimes \mathbb{1}^{(i+1)}_c \otimes h_q && \text{(E.32c)}\\
&+ |L\rangle\langle L|^{(i)}_c \otimes (\mathbb{1}_{eq} - |\circledless\rangle\langle\circledless|)^{(i)} \otimes \mathbb{1}^{(i+1)}_{ceq} && \text{(E.32d)}\\
&+ (\mathbb{1}_c - |L\rangle\langle L|_c)^{(i)} \otimes |\circledless\rangle\langle\circledless|^{(i)} \otimes \mathbb{1}^{(i+1)}_{ceq} && \text{(E.32e)}\\
&+ \mathbb{1}^{(i)}_{ceq} \otimes |R\rangle\langle R|^{(i+1)}_c \otimes (\mathbb{1}_{eq} - |\circledgtr\rangle\langle\circledgtr|)^{(i+1)} && \text{(E.32f)}\\
&+ \mathbb{1}^{(i)}_{ceq} \otimes (\mathbb{1}_c - |R\rangle\langle R|)^{(i+1)}_c \otimes |\circledgtr\rangle\langle\circledgtr|^{(i+1)} && \text{(E.32g)}\\
&+ \mathbb{1}^{(i)}_c \otimes |0\rangle\langle 0|^{(i)}_e \otimes |R\rangle\langle R|^{(i+1)}_c \otimes \mathbb{1}^{(i+1)}_{eq} && \text{(E.32h)}\\
&+ |L\rangle\langle L|^{(i)}_c \otimes \mathbb{1}^{(i)}_{eq} \otimes \mathbb{1}^{(i+1)}_c \otimes |0\rangle\langle 0|^{(i+1)}_e && \text{(E.32i)}\\
&+ \mathbb{1}^{(i)}_c \otimes |0\rangle\langle 0|^{(i)}_e \otimes (\mathbb{1}_c - |L\rangle\langle L|)^{(i+1)}_c \otimes (\mathbb{1}_{eq} - |0\rangle\langle 0|)^{(i+1)}_e && \text{(E.32j)}\\
&+ (\mathbb{1}_c - |R\rangle\langle R|)^{(i)}_c \otimes (\mathbb{1}_{eq} - |0\rangle\langle 0|)^{(i)}_e \otimes \mathbb{1}^{(i+1)}_c \otimes |0\rangle\langle 0|^{(i+1)}_e , && \text{(E.32k)}\\
&+ \mathbb{1}^{(i)}_{ceq} \otimes \mathbb{1}^{(i+1)}_{ceq} && \text{(E.32l)}\\
h^{(1)}_i =& -(1 + \alpha_2(\varphi))\mathbb{1}^{(i)}_{ceq}, && \text{(E.32m)}
\end{aligned}
$$

where

$$\alpha_2(\varphi) := \sum_{4^n+7>|\varphi|} 4^{-2n-1}\lambda_0(H_q(4^n)), \tag{E.33}$$

as defined in Proposition 53 of [1]. Then the k times renormalised Hamiltonian $R^{(k)}(H_u)^{\Lambda(L\times H)}$ has the following properties:

1. *For any finite region of the lattice, the restriction of the Hamiltonian to that region has an eigenbasis of the form $|T\rangle_c \otimes |\psi_i\rangle$ where $|T\rangle_c$ is a classical tiling state (cf. Lemma 51 of [1]).*

2. *Furthermore, for any given $|T\rangle_c$, the lowest energy choice for $|\psi\rangle_q$ consists of ground states of $R^{(k)}(H_q)(r)$ on segments between sites in which*

$|T\rangle_c$ contains an $\left|R^{(k)}(L)\right\rangle$ and an $\left|R^{(k)}(R)\right\rangle$, a 0-energy eigenstate on segments between an $\left|R^{(k)}(L)\right\rangle$ or $\left|R^{(k)}(R)\right\rangle$ and the boundary of the region, and $|e\rangle$'s everywhere else. Any eigenstate which is not an eigenstate of $R^{(k)}(H_q)(r)$ on segments between sites in which $|T\rangle_c$ contains an $\left|R^{(k)}(L)\right\rangle$ and an $\left|R^{(k)}(R)\right\rangle$ has an energy > 1 (cf. Lemma 51 of [1]).

3. *The ground state energy is contained in the interval*

$$\Bigg[(g(k) - 4^k\alpha_2(\varphi))LH - 2^{-k}H + \sum_{n=1}^{\lfloor \log_4(L/2)\rfloor}\Bigg(\left\lfloor \frac{H}{2^{2n+1(k\ mod2)}}\right\rfloor \tag{E.34}$$

$$\times\left(\left\lfloor \frac{L}{2^{2n+1-(k\ mod2)}}\right\rfloor - 1\right)\Bigg)\lambda_0(R^{(k)}(H_q)(4^{n-\lfloor (k\ mod2)/2\rfloor})), \tag{E.35}$$

$$(g(k) - 4^k\alpha_2(\varphi))LH - 2^{-k}H + \sum_{n=1}^{\lfloor \log_4(L/2)\rfloor}\left(\left(\left\lfloor \frac{H}{2^{2n+1-(k\ mod2)}}\right\rfloor + 1\right)\right. \tag{E.36}$$

$$\left.\times\left\lfloor \frac{L}{2^{2n+1-(k\ mod2)}}\right\rfloor\right)\lambda_0(R^{(k)}(H_q)(4^{n-\lfloor (k\ mod2)/2\rfloor}))\Bigg] \tag{E.37}$$

where

$$g(k) = 4^k \sum_{4^n+1<2^k} 4^{-2n-1}\lambda_0(H_q(4^n)), \tag{E.38}$$

(cf. Lemma 52 of [1]).

To help the reading flow, we postpone the proof of this Lemma to Section F.

Lemma 34. *Let $S_{br}(k)$ be the subspace spanned by states for which the left-most site is of the form $\left|e^{\times p}\textcircled{<}\{x\}^{\times 2^k-p-1}\right\rangle$ for a fixed integer $1 \le p \le 2^k$ and the right-most site is of the form $\left|\{y\}^{\times 2^k-q-1}\textcircled{>}e^{\times q}\right\rangle$ for fixed integer $1 \le q \le 2^k$. Then*

$$\lambda_0(R^{(k)}(H_q)(L)|_{S_{br}(k)}) = \min_{2^{k-1}L+1\le x\le 2^kL} \lambda_0(H_q(x)) \tag{E.39}$$

Proof. $R^{(k)}(h_q)$ is block-diagonal with respect to the subspaces of $R^{(k)}(\mathcal{H}_{eq})^{\otimes 2}$ spanned by products of $\left|e^{\times p}\textcircled{<}\{x\}^{\times 2^k-p-1}\right\rangle$ and $\left|\{y\}^{\times 2^k-q-1}\textcircled{>}e^{\times q}\right\rangle$ for fixed p, q, together with the orthogonal complement thereof, while acting as identity on $R^{(k)}(\mathcal{H}_c)^{\otimes 2}$.

Thus the ground state energy is equal to $\min_{2^{k-1}L+1\leq x\leq 2^k L} \lambda_0(H_q(x))$.

□

Corollary 35. *If* $\lim_{L\to\infty} \lambda_0(H_u^{\Lambda(L)}) = +\infty$*, then* $\lim_{L\to\infty} \lambda_0(R^{(k)}(H_u)^{\Lambda(L)}) = +\infty$ *for all* $k \geq k_0(|\varphi|)$*, and* $k_0(|\varphi|)$ *is the smallest integer such that* $2^{k_0} > |\varphi|+7$*. If* $\lim_{L\to\infty} \lambda_0(H_u^{\Lambda(L)}) = -\infty$*, then* $\lim_{L\to\infty} \lambda_0(R^{(k)}(H_u)^{\Lambda(L)}) = -\infty$ *for all* $k \geq k_0(\varphi)$*.*

Proof. Consider applying the RG mapping $k > k_0(\varphi)$ times, then we see that

$$g(k) = 4^k \sum_{4^n+1<2^k} 4^{-2n-1}\lambda_0(H_q(4^n)) \tag{E.40}$$

$$= 4^k \sum_{4^n+1<2^{k_0}} 4^{-2n-1}\lambda_0(H_q(4^n)) + 4^k \sum_{2^{k_0}<4^n+1<2^k} 4^{-2n-1}\lambda_0(H_q(4^n)) \tag{E.41}$$

$$= 4^k\alpha_2(\varphi) + 4^k \sum_{2^{k_0}<4^n+1<2^k} 4^{-2n-1}\lambda_0(H_q(4^n)). \tag{E.42}$$

From Lemma 33, the interval the ground state energy is contained in is

$$\Bigg[LH \sum_{2^{k_0}<4^n+1<2^k} 4^{-2n-1}4^k\lambda_0(H_q(4^n)) - 2^{-k}H + \sum_{n=1}^{\lfloor \log_4(L/2)\rfloor} \left(\left\lfloor \frac{H}{2^{2n+1-(k\ mod 2)}}\right\rfloor \left(\left\lfloor \frac{L}{2^{2n+1-(k\ mod 2)}}\right\rfloor - 1\right)\right) \lambda_0(R^{(k)}(H_q)(4^{n-\lfloor (k\ mod 2)/2\rfloor})),$$
$$LH \sum_{2^{k_0}<4^n+1<2^k} 4^{-2n-1}4^k\lambda_0(H_q(4^n)) - 2^{-k}H + \sum_{n=1}^{\lfloor \log_4(L/2)\rfloor} \left(\left(\left\lfloor \frac{H}{2^{2n+1-(k\ mod 2)}}\right\rfloor + 1\right) \left\lfloor \frac{L}{2^{2n+1-(k\ mod 2)}}\right\rfloor\right) \lambda_0(R^{(k)}(H_q)(4^{n-\lfloor (k\ mod 2)/2\rfloor}))\Bigg]. \tag{E.43}$$

From Lemma 34, if $\lambda_0(H_q(4^n+1)) = 0$ for all n, then $\lambda_0(R^{(k)}(H_q)(4^n+1)) = 0$ for all n. In this case the ground state energy becomes $\lambda_0(R^{(k)}(H)^{\Lambda(L)}) = -2^{-k}L \xrightarrow{L\to\infty} -\infty$.

We see that if for any n_0, $\lambda_0(H_q(4^{n_0}+1)) > 0$, then $\lambda_0(R^{(k)}(H_q)(4^n + 1)) > 0\ \forall n \geq n_0'$ (n_0' not necessarily equal to n_0). Define $g(k) = \eta(k) + 4^k\alpha_2(\varphi)$ then $\eta(k) \geq 0$, and we see that the lower bound of the ground state

is

$$L^2\eta(k) - 2^{-k}L + \sum_{n=1}^{\lfloor \log_4(L/2) \rfloor} \left(\left\lfloor \frac{L}{2^{2n+1-(k\ mod2)}} \right\rfloor \left(\left\lfloor \frac{L}{2^{2n+1-(k\ mod2)}} \right\rfloor - 1 \right) \right) \times$$
$$\lambda_0(R^{(k)}(H_q)(4^{n-\lfloor (k\ mod2)/2 \rfloor})) \xrightarrow{L\to\infty} +\infty. \tag{E.44}$$

□

For $2^{k_0} \leq |\varphi| + 7$ the above relationship is not necessarily preserved. To see why, note that for lengths $\ell \leq |\varphi| + 7$ the Gottesman-Irani Hamiltonian will not encode the correct computation and hence will pick up some energy. Since $\lambda_0(R^{(k)}(H_q)(L)|_{S_{br}}) = \min_{2^{k-1}L+1 \leq x \leq 2^k L} \lambda_0(H_q(x))$ rather than $\lambda_0(R^{(k)}(H_q)(L)|_{S_{br}}) = \lambda_0(H_q(x))$, the energies in the summation term and the α_2 term will not exactly cancel out until we reach higher order steps of the RG flow. This is only rectified once we reach $2^{k_0} > |\varphi| + 7$ as the energy integrated out by the projector Π_{gs}, as given in Definition 29, is exactly $\lambda_0(H_q(x))$, not $\lambda_0(R^{(k)}(H_q)(L)|_{S_{br}})$.

E.3 Renormalising H_d

The only part of the Hamiltonian acting on $\mathcal{H}_d$ is H_d; there is no coupling to other parts of the Hilbert space and so we can renormalise this part independently. For concreteness, following [1], we will let H_d be the critical XY-model with local terms $X_i \otimes X_{i+1} + Y_i \otimes Y_{i+1} + \mu/2(Z_i \otimes \mathbb{1}^{(i+1)} + \mathbb{1}^{(i)} \otimes Z_{i+1})$, which can be written as:

$$h_d^{row(i,i+1)} = X_i \otimes X_{i+1} + Y_i \otimes Y_{i+1}, \tag{E.45}$$
$$h_d^{col(i,i+1)} = 0, \tag{E.46}$$
$$h_d^{(1)(i)} = \mu Z_i. \tag{E.47}$$

This has zero gap for any $0 \leq \mu < 1$. However any Hamiltonian with a dense spectrum in the thermodynamic limit could be substituted. Since the critical XY model is critical, it forms a fixed point in any reasonable RG scheme.

In particular, Penson, Jullien and Pfeuty apply the BRG to renormalise this model [9]. Notably, they show that there are multiple fixed points depending on the block size used: here since we are interested in blocking 2×2 blocks, we choose a block size of 2, and set μ equal to one of

the relevant fixed point values, ensuring a gapless spectrum. The authors demonstrate that the coefficient of $h_d^{row(i,i+1)}$ and Z_i terms maintain a constant ratio to each other. This RG scheme can then be expressed in terms of an RG isometry V^d. Since the two coefficients maintain a constant ratio, the renormalisation unitary simply rescales these two terms.

E.4 Renormalising $|0\rangle$

If we wish to preserve the form of the possible ground states depending, it is straightforward to see that this can be done if the states $|0\rangle$ simply get mapped to themselves $|0\rangle^{\otimes(2\times 2)} \to |0\rangle$ under the RG operation. This can be implemented using the isometry

$$V^0_{(i,i+1),(j,j+1)} := |0\rangle_{(i/2,j/2)} \langle 0|_{(i,j)} \langle 0|_{(i+1,j)} \langle 0|_{(i,j+1)} \langle 0|_{(i+1,j+1)} . \tag{E.48}$$

E.5 The Overall Renormalised Hamiltonian

Accounting for the renormalisation of all the different parts of the Hamiltonian, we can now define renormalisation group mapping for the entire Hamiltonian. Recall that the original local terms are:

$$h(\varphi)^{(i,j)} = |0\rangle \langle 0|^{(i)} \otimes (\mathbb{1} - |0\rangle \langle 0|)^{(j)} + (\mathbb{1} - |0\rangle \langle 0|)^{(i)} \otimes |0\rangle \langle 0|^{(j)} \tag{E.49}$$

$$+ h_u^{(i,j)}(\varphi) \otimes \mathbb{1}_d^{(i,j)} + \mathbb{1}_u^{(i,j)} \otimes h_d^{(i,j)} \tag{E.50}$$

$$h(\varphi)^{(1)} = -(1+\alpha_2(\varphi))\Pi_{ud}, \tag{E.51}$$

where $\alpha_2(\varphi)$ is defined in Lemma 33 and $\Pi_{ud}^{(j)} = (\mathbb{1} - |0\rangle \langle 0|)^{(j)}$.

Definition 36 (Full Renormalisation Group Mapping)**.** *Let V^u, V^0, V^d be the isometries defined in Definition 30, eq. (E.48), and Subsection E.3 respectively. Define*

$$V^r_{(i,i+1),(j,j+1)} := V^0_{(i,i+1),(j,j+1)} \oplus \left(V^u_{(i,i+1),(j,j+1)} \otimes V^d_{(i,i+1),(j,j+1)}\right). \tag{E.52}$$

Then the overall RG mapping of local Hamiltonian terms is given by

$$\mathcal{R} : h(\varphi)^{(i,i+1)} \mapsto V^{r\dagger}_{(i,i+1),(j,j+1)} h(\varphi)^{(i,i+1)} V^r_{(i,i+1),(j,j+1)} \tag{E.53}$$

$$\mathcal{R} : h(\varphi)^{(i+1,i+2)} \mapsto V^{r\dagger}_{(i+2,i+3),(j,j+1)} h(\varphi)^{(i+1,i+2)} V^r_{(i,i+1),(j,j+1)} V^r_{(i+2,i+3),(j,j+1)} \tag{E.54}$$

Lemma 37. *Applying the RG mapping from Definition 36 to the terms in eq. (E.49) we see that the renormalised 1- and 2-local terms become:*

$$R^{(k)}(h(\varphi))^{(i,j)} = 2^k(|0\rangle\langle 0|^{(i)} \otimes \Pi_{ud}^{(j)} + \Pi_{ud}^{(i)} \otimes |0\rangle\langle 0|^{(j)}) \tag{E.55}$$

$$+ R^{(k)}(h_u(\varphi))^{(i,j)} \otimes \mathbb{1}_d^{(i,j)} + \mathbb{1}_u^{(i,j)} \otimes R^{(k)}(h_d^{(i,j)}) \tag{E.56}$$

$$R^{(k)}(h(\varphi))^{(1)} = (g(k) - 4^k\alpha_2(\varphi) - 2^k)\Pi_{ud}^{(i)} + R^{(k)}(h_u^{(1)})^{(i)} \tag{E.57}$$

where $g(k)$ is defined in Lemma 33. All the terms are computable.

Proof. Note that the RG isometry acts block-diagonally with respect to the subspaces spanned by $|0\rangle^{\otimes(2\times2)}$ and those spanned by states in $(R^{(k)}(\mathcal{H}_u) \otimes \mathcal{H}_d)^{\otimes(2\times2)}$. Furthermore, any state which is not in one of the two subspaces is projected out. The $h_u(\varphi)$, h_d and 1-local terms transform as they would in the absence of the $|0\rangle$ state, thus giving the terms seen above. The explicit coefficients are calculated in Lemma 46 in the appendix. The term $g(k)$ is computable for any k by calculating the $\lambda_0(H_q)(4^n+1)$ for all $n \le 2k+1$. Since this is a finite dimensional matrix for any finite n, this is a computable quantity.

The form of the overall renormalisation isometry means the $|0\rangle\langle 0|^{(i)} \otimes \Pi_{ud}^{(j)}$ term must be preserved in form, however, we note that because all states of 2×2 blocks in different subspaces in the previous RG step must be in $|0\rangle^{\otimes(2\times2)}$ or $(R^{(k)}(\mathcal{H}_u) \otimes R^{(k)}(\mathcal{H})_d)^{\otimes(2\times2)}$, then two neighbouring blocks must pick up an energy penalty of $\times 2$ of the previous local terms. □

Corollary 38. *The local terms of the initial Hamiltonian $h(\varphi)$ and all further renormalised local terms belong to a family of Hamiltonians $\mathcal{F}(\varphi, \tau_1, \tau_2, \{\alpha_i\}_i, \{\beta_i\}_i)$, which all take the form:*

$$R^{(k)}(h(\varphi))^{(i,j)} = \tau_1(|0\rangle\langle 0|^{(i)} \otimes \Pi_{ud}^{(j)} + \Pi_{ud}^{(i)} \otimes |0\rangle\langle 0|^{(j)}) \tag{E.58}$$

$$+ R^{(k)}(h_u(\varphi, \{\beta_t\}_t))^{(i,j)} \otimes \mathbb{1}_d^{(i,j)} + \mathbb{1}_u^{(i,j)} \otimes R^{(k)}(h_d)^{(i,j)} \tag{E.59}$$

$$R^{(k)}(h(\varphi))^{(1)} = \tau_2\Pi_{ud} + R^{(k)}(h_u(\varphi, \{\alpha_t\}_t))^{(1)}, \tag{E.60}$$

where the sets $\{\alpha_t\}_t$, $\{\beta_i\}$ characterises the parameters of the renormalised Gottesman-Irani Hamiltonian. Furthermore, for any $k \in \mathbb{N}$, the coefficients $\tau_1(k)$, $\tau_2(k)$, $\{\alpha_t(k)\}_t$ and $\{\beta_t(k)\}_t$ are computable.

Proof. Follows immediately from Lemma 37. □

Lemma 39. *Let $R^{(k)}(h(\varphi))^{(i,j)}$, $R^{(k)}(h(\varphi))^{(1)}$ be the local terms defined by the RG mapping in Definition 36 for any $k > k_0(|\varphi|)$. The Hamiltonian $R^{(k)}(H)$ defined by these terms then has the following properties:*

1. *If the unrenormalised Hamiltonian $H(\varphi)$ has a zero energy ground state with a spectral gap of 1/2, then $R^{(k)}(H)$ also has a zero energy ground state with zero correlations functions, and has a spectral gap of $\geq 2^k$.*
2. *If the unrenormalised Hamiltonian $H(\varphi)$ has a ground state energy $-\infty$ with a dense spectrum above this, then $R^{(k)}(H)$ also a ground state energy of $-\infty$ with a dense spectrum, and has algebraically decaying correlation functions.*

Proof. First examine the spectrum of the renormalised Hamiltonian from Lemma 37: for convenience let:

$$R^{(k)}(h_0)^{(i,j)} := 2^k(|0\rangle\langle 0|^{(i)} \otimes \Pi_{ud}^{(j)} + |0\rangle\langle 0|^{(i)} \otimes \Pi_{ud}^{(j)}).$$

Further let

$$R^{(k)}(H_0^{\Lambda(L)}) := \sum_{\langle i,j\rangle} R^{(k)}(h_0)^{(i,j)}, \tag{E.61}$$

$$R^{(k)}(\tilde{H}_u)^{\Lambda(L)} := \sum_{\langle i,j\rangle} \mathbb{1}_d^{(i,j)} \otimes R^{(k)}(h_u)^{(i,j)} \tag{E.62}$$

$$R^{(k)}(\tilde{H}_d)^{\Lambda(L)} := \sum_{\langle i,j\rangle} \mathbb{1}_u^{(i,j)} \otimes R^{(k)}(h_d)^{(i,j)} \tag{E.63}$$

We note $R^{(k)}(H_0)^{\Lambda}$,$R^{(k)}(\tilde{H}_d)^{\Lambda}$, $R^{(k)}(\tilde{H}_u)^{\Lambda}$ all commute. Further note that:

$$\operatorname{spec} R^{(k)}(H_0)^{\Lambda} \subset 2^k\mathbb{Z}_{\geq 0}. \tag{E.64}$$

If $\lambda_0(H(\varphi)) = 0$, then it implies $\lambda_0(H_u(\varphi)) \to +\Omega(L^2)$ (see Subsection A.4). By Corollary 35, this implies $\lambda_0(R^{(k)}(H_u(\varphi))) \to +\Omega(L^2)$ too. Hence the ground state is the zero-energy $|0\rangle^{\Lambda(L)}$ state. Since $\operatorname{spec}(R^{(k)}(H_0)^{\Lambda}) \subset 2^k\mathbb{Z}_{\geq 0}$, then the first excited state (provided L is sufficiently larger) has energy at least 2^k. Finally, the state $|0\rangle^{\Lambda(L)}$ has zero correlations.

If $\lambda_0(H(\varphi)) = -\Omega(L)$, then $\lambda_0(H_u(\varphi)) \to -\Omega(L)$ (see Subsection A.4). By Corollary 35, this implies $\lambda_0(R^{(k)}(H)) \to -\Omega(L)$. Since $\operatorname{spec}(R^{(k)}(H_0)^{\Lambda}) \subset 2^k\mathbb{Z}_{\geq 0}$, then the ground state is the ground state of $R^{(k)}(\tilde{H}_d)^{\Lambda(L)}+R^{(k)}(\tilde{H}_u)^{\Lambda(L)}$.

Since $\mathrm{spec}(R^{(k)}(\tilde{H}_d)^{\Lambda(L)})$ becomes dense in the thermodynamic limit, we see that the overall Hamiltonian $R^{(k)}(H(\varphi))^{\Lambda(L)}$ has a dense spectrum in the thermodynamic limit $L \to \infty$. Let $|\psi\rangle_u$ and $|\phi\rangle_d$ be the ground states of $R^{(k)}(H_u)^{\Lambda(L)}$ and $R^{(k)}(H_d)^{\Lambda(L)}$ respectively, then the ground state of $R^{(k)}(\tilde{H}_d)^{\Lambda(L)} + R^{(k)}(\tilde{H}_u)^{\Lambda(L)}$ is $|\psi\rangle_u |\phi\rangle_d$ Since $R^{(k)}(H_d)^{\Lambda(L)}$ is just the rescaled critical XY-model (as per Subsection E.3) and its ground state has algebraically decaying correlations [2], hence the overall ground state has algebraically decaying correlations. □

E.6 Order Parameter Renormalisation

In Subsection A.3.3 we saw that the observable $O_{A/B}(r)$ functioned as an order parameter which distinguished the two phases. Defining $V_r := V^0_{(i,i+1),(j,j+1)} \oplus \left(V^u_{(i,i+1),(j,j+1)} \otimes V^d_{(i,i+1),(j,j+1)}\right)$, and $V_r[k]$ as the corresponding isometry for the kth step of the RG process, then define:

$$R^{(k)}(O_{A/B})(r) := V^r[k] O_{A/B}(2^k r) V^{r\dagger}[k]. \tag{E.65}$$

The following lemma then holds:

Lemma 40. *Let $|\psi_{gs}\rangle$ be the ground state of H_u. The expectation value of the order parameter satisfies:*

$$\langle\psi_{gs}|\, R^{(k)}(O_{A/B})(r)\, |\psi_{gs}\rangle = \begin{cases} 1 & \textit{if } \lambda_0(R^{(k)}(H(\varphi))) = 0 \\ 0 & \textit{if } \lambda_0(R^{(k)}(H(\varphi))) = -\Omega(L). \end{cases} \tag{E.66}$$

Proof. If $\lambda_0(R^{(k)}(H)) \to -\Omega(L)$, then the ground state is that of $H_u^{(\Lambda(L))}$, and hence the state $|0\rangle$ does not appear anywhere in the ground state.
If $\lambda_0(R^{(k)}(H)) = 0$, the ground state is $|0\rangle^{\Lambda(L)}$. Since, under $V_r[k]$, $|0\rangle^{\otimes 2^k \times 2^k} \to |0\rangle$, the lemma follows. □

Thus the renormalised order parameter still acts as an order parameter for the renormalised Hamiltonian. In particular, it still undergoes a non-analytic change when moving between phases.

E.7 Uncomputability of RG flows

We finally have all the ingredients for the proof of our two main results.

Theorem 41 (Exact RG flow for undecidable Hamiltonian, Formal Restatement of Theorem 1 in the main manuscript)**.** *Let H be the Hamiltonian defined in [1]. The renormalisation group procedure, defined in Definition 36, has the following properties:*

1. *$\mathcal{R}(h)$ is computable.*

2. *If $H(\varphi)$ is gapless, then $R^{(k)}(H(\varphi))$ is gapless, and if $H(\varphi)$ is gapped, then $R^{(k)}(H(\varphi))$ is gapped.*

3. *For the order parameter of the form $O_{A/B}(r)$ which distinguished the phases of $H^{\Lambda(L)}$, there exists a renormalised observable $R^{(k)}(O_{A/B})(r)$ which distinguishes the phases of $R^{(k)}(H)^{\Lambda(L)}$ and is non-analytic at phase transitions.*

4. *For k iterations, the renormalised local interactions of $R^{(k)}(H)$ are computable and belong to the family $\mathcal{F}(\varphi, \tau_1, \tau_2, \{\beta_i\})$, as defined in Corollary 38.*

5. *If $H(\varphi)$ initially has algebraically decaying correlations, then $R^{(k)}(H(\varphi))$ also has algebraically decaying correlations. If $H(\varphi)$ initially has zero correlations, then $R^{(k)}(H(\varphi))$ also has zero correlations.*

Proof. Claim 1 follows from Definition 36, where the renormalisation isometries and subspace restrictions are explicitly written down and are manifestly computable, and hence for any k the coefficients in Lemma 37 are computable. Claim 2 follows from Lemma 39: we see that, for all $k > k_0$ the spectrum below energy 2^{k-1} is either dense with a ground state with energy at $-\infty$, or is empty except for a single zero energy state, corresponding to the gapped and gapless cases of $H(\varphi)$. Claim 3 follows from Lemma 40. Claim 4 follows from Corollary 38. Claim 5 follows from the properties of the ground states in the cases $\lambda_0(H_u^{\Lambda(L)}) \to \pm\infty$ and by Lemma 39.

□

Theorem 42 (Uncomputability of RG flow)**.** *Let $h(\varphi)$, $\varphi \in \mathbb{Q}$, be the full local interaction of the Hamiltonian from [1]. Consider k iterations of the RG map $\mathcal{R}$ from Definition 36 acting on $H(\varphi)$; the renormalised local terms are given by $R^{(k)}(h(\varphi))$ and can be parameterised as per Corollary 38.*

If the UTM is non-halting on input φ, then for all $k > k_0(\varphi)$ we have that $\tau_2(k) = -2^k$, for some computable $k_0(\varphi)$. If the UTM halts on input φ, then

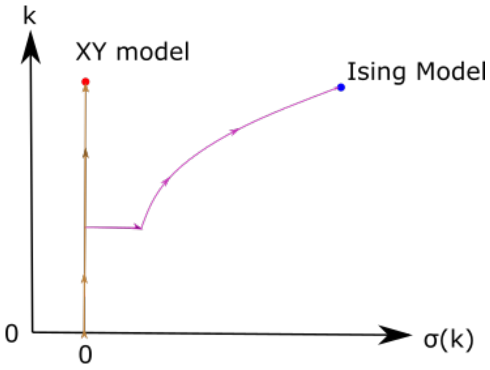

Supplementary Figure 10: chaotic RG flow behaviour in parameter space. The orange line represents the trajectory of the observable σ starting at some value $\varphi = \varphi_0$ for which the QTM does not halt, while purple represents $\varphi = \varphi_0 + \epsilon$ for any algebraic number ϵ for which the QTM halts. For small k, the orange and purple lines coincide. Then, at a particular (but uncomputable) value of k, $\sigma(k)$ becomes non-zero and from that point it increases exponentially.

there exists an uncomputable $k_h(\varphi)$ such that for $k_0(\varphi) < k < k_h(\varphi)$ we have $\tau_2(k) = -2^k$, and for all $k > k_h(\varphi)$ then $\tau_2(k) = -2^k + \Omega(4^{k-k_h(\varphi)})$.

Proof. Consider the expression for τ_2 from Lemma 37:

$$\tau_2(k) = 4^k \sum_{4^n+1<2^k} 4^{-2n-1}\lambda_0(H_q(4^n)) + 4^k\alpha_2(\varphi) - 2^k. \tag{E.67}$$

From the definition of $\alpha_2(\varphi)$, we see that there is a $k_0(\varphi)$ such that $g(k_0(\varphi)) = \alpha_2(\varphi)$ (where $g(k)$ is defined in eq. (E.38)), and hence we get:

$$\tau_2(k) = -2^k + 4^k \sum_{2^{k_0(\varphi)}<4^n+1<2^k} 4^{-2n-1}\lambda_0(H_q(4^n)). \tag{E.68}$$

If the encoded QTM never halts, then by Lemma 19 $\lambda_0(H_q(4^n)) = 0$ for all n such that $4^n+1 > 2^{k_0(\varphi)}$. If the encoded UTM halts then by Lemma 19 there exists an n_0 such that $\lambda_0(H_q(4^n)) > 0$ for all $n > n_0$. Then $k_h(\varphi)$ is defined as the minimum k such that $4^{n_0} + 1 < 2^{k_h(\varphi)}$. Thus determining $k_h(\varphi)$ is at least as hard as computing the halting time and thus is an uncomputable number.

□

F Proof of Lemma 33

For convenience we state Lemma 51 of [1].

Lemma 43 (Tiling + quantum layers, Lemma 51 of [1]). *Let $h_c^{\text{row}}, h_c^{\text{col}} \in \mathcal{B}(\mathbb{C}^C \otimes \mathbb{C}^C)$ be the local interactions of a 2D tiling Hamiltonian H_c, with two distinguished states (tiles) $|L\rangle, |R\rangle \in \mathbb{C}^C$. Let $h_q \in \mathcal{B}(\mathbb{C}^Q \otimes \mathbb{C}^Q)$ be the local interaction of a Gottesman-Irani Hamiltonian $H_q(r)$, as in Section D. Then there is a Hamiltonian on a 2D square lattice with nearest-neighbour interactions $h^{\text{row}}, h^{\text{col}} \in \mathcal{B}(\mathbb{C}^{C+Q+1} \otimes \mathbb{C}^{C+Q+1})$ with the following properties: For any region of the lattice, the restriction of the Hamiltonian to that region has an eigenbasis of the form $|T\rangle_c \otimes |\psi\rangle_q$, where $|T\rangle_c$ is a* product *state representing a classical configuration of tiles. Furthermore, for any given $|T\rangle_c$, the lowest energy choice for $|\psi\rangle_q$ consists of ground states of $H_q(r)$ on segments between sites in which $|T\rangle_q$ contains an $|L\rangle$ and an $|R\rangle$, a 0-energy eigenstate on segments between an $|L\rangle$ or $|R\rangle$ and the boundary of the region, and $|e\rangle$'s everywhere else.*

For the rest of this section we denote $\left|R^{(k)}(L)\right\rangle$ and $\left|R^{(k)}(R)\right\rangle$ to be the states in the set of k-time renormalised Robinson tiles with a down-left and down-right red cross marking on them, respectively. $|L\rangle$ and $|R\rangle$ will be the unrenormalised versions of these tiles. (These can be seen as the first two cross tiles on the left in Supplementary Fig. 1, where the internal markings are coloured red.) For simplicity we break down Lemma 33 into two separate parts: the first deals with the first two claims and the last deals with the third claim.

Lemma 44 (Restatement of Claim 1 and 2 in Lemma 33). *Let $H_u^{\Lambda(L)} =$*

$\sum h_u^{row(j,j+1)} + \sum h_u^{col(i,i+1)}$, *where*

$$h^{\mathrm{col}}_{j,j+1} = h^{\mathrm{col}}_c \otimes \mathbb{1}^{(j)}_{eq} \otimes \mathbb{1}^{(j+1)}_{eq} \tag{F.1a}$$

$$h^{\mathrm{row}}_{i,i+1} = h^{\mathrm{row}}_c \otimes \mathbb{1}^{(i)}_{eq} \otimes \mathbb{1}^{(i+1)}_{eq} \tag{F.1b}$$

$$+ \mathbb{1}^{(i)}_c \otimes \mathbb{1}^{(i+1)}_c \otimes h_q \tag{F.1c}$$

$$+ |L\rangle\langle L|^{(i)}_c \otimes (\mathbb{1}_{eq} - |\textcircled{<}\rangle\langle\textcircled{<}|)^{(i)} \otimes \mathbb{1}^{(i+1)}_{ceq} \tag{F.1d}$$

$$+ (\mathbb{1}_c - |L\rangle\langle L|_c)^{(i)} \otimes |\textcircled{<}\rangle\langle\textcircled{<}|^{(i)} \otimes \mathbb{1}^{(i+1)}_{ceq} \tag{F.1e}$$

$$+ \mathbb{1}^{(i)}_{ceq} \otimes |R\rangle\langle R|^{(i+1)}_c \otimes (\mathbb{1}_{eq} - |\textcircled{>}\rangle\langle\textcircled{>}|)^{(i+1)} \tag{F.1f}$$

$$+ \mathbb{1}^{(i)}_{ceq} \otimes (\mathbb{1}_c - |R\rangle\langle R|)^{(i+1)}_c \otimes |\textcircled{>}\rangle\langle\textcircled{>}|^{(i+1)} \tag{F.1g}$$

$$\tag{F.1h}$$

$$+ \mathbb{1}^{(i)}_c \otimes |e\rangle\langle e|^{(i)}_e \otimes |R\rangle\langle R|^{(i+1)}_c \otimes \mathbb{1}^{(i+1)}_{eq} \tag{F.1i}$$

$$+ |L\rangle\langle L|^{(i)}_c \otimes \mathbb{1}^{(i)}_{eq} \otimes \mathbb{1}^{(i+1)}_c \otimes |e\rangle\langle e|^{(i+1)}_e \tag{F.1j}$$

$$+ \mathbb{1}^{(i)}_c \otimes |e\rangle\langle e|^{(i)}_e \otimes (\mathbb{1}_c - |L\rangle\langle L|)^{(i+1)}_c \otimes (\mathbb{1}_{eq} - |e\rangle\langle e|)^{(i+1)}_e \tag{F.1k}$$

$$+ (\mathbb{1}_c - |R\rangle\langle R|)^{(i)}_c \otimes (\mathbb{1}_{eq} - |e\rangle\langle e|)^{(i)}_e \otimes \mathbb{1}^{(i+1)}_c \otimes |e\rangle\langle e|^{(i+1)}_e \tag{F.1l}$$

$$+ \mathbb{1}^{(i)}_{ceq} \otimes \mathbb{1}^{(i+1)}_{ceq} \tag{F.1m}$$

$$h^{(1)}_i = -(1 + \alpha_2(\varphi))\mathbb{1}^{(i)}_{ceq}, \tag{F.1n}$$

for a constant $\alpha_2(\varphi)$.

Then the k *times renormalised Hamiltonian under the RG mapping of Definition 30,* $R^{(k)}(H_u)^{\Lambda(L\times H)}$*, has the following properties:*

1. *For any finite region of the lattice, the restriction of the Hamiltonian to that region has an eigenbasis of the form* $|T\rangle_c \otimes |\psi_i\rangle$ *where* $|T\rangle_c \in R^{(k)}(\mathcal{H}_c)^{\Lambda(L\times H)}$ *is a classical tiling state,* $|\psi_i\rangle \in R^{(k)}(\mathcal{H}_{eq})^{\Lambda(L\times H)}$.

2. *Furthermore, for any given* $|T\rangle_c$*, the lowest energy choice for* $|\psi\rangle_q$ *consists of ground states of* $R^{(k)}(H_q)(r)$ *on segments between sites in which* $|T\rangle_c$ *contains an* $|R^{(k)}(L)\rangle$ *and an* $|R^{(k)}(R)\rangle$*, a 0-energy eigenstate on segments between an* $|R^{(k)}(L)\rangle$ *or* $|R^{(k)}(R)\rangle$ *and the boundary of the region, and* $|e^{\times 2^k}\rangle$*'s everywhere else. Any eigenstate which is not an eigenstate of* $R^{(k)}(H_q)(r)$ *on segments between sites in which* $|T\rangle_c$ *contains an* $|R^{(k)}(L)\rangle$ *and an* $|R^{(k)}(R)\rangle$ *has an energy* > 1.

Proof.

Claim 1
The fact the eigenstates of the unrenormalised Hamiltonian are a product state across $\mathcal{H}_c$ and $\mathcal{H}_{eq}$, $|T_c\rangle |\psi\rangle_{eq'}$ is from Lemma 43 (Lemma 51 of [1]). The structure of the eigenstates of the renormalised Hamiltonian is then preserved as per Lemma 26.

Claim 2
Start by considering what each of the local terms looks like after applying the renormalisation isometries. We treat each term in the above lemma in succession. Start with local interactions encoding the classical tiling, terms F.1a and F.1b. The isometry decomposes as $V^u_{(i,i+1)(j,j+1)} = (\mathbb{1}\otimes\Pi_{gs})V^c_{(i,i+1)(j,j+1)}\otimes V^{eq}_{(i,i+1)(j,j+1)}$, hence the classical Hamiltonian terms transform as per Lemma 12.

We next consider the renormalisation of the Gottesman-Irani Hamiltonian h_q. All of these states are mapped by $V^{eq}_{(i,i+1)(j,j+1)}$ to a 2×1 chain, which $V^q_{(i,i+1)}$ acts on as per Lemma 23. Thus h_q transforms as per Lemma 23.

Coupling Terms
We first note that given a 2×2 block, we will get two sets of coupling terms: one between c and eq_1 and another set between c and eq_2. Thus the terms will have the structure $h_c^{i,i+1}\otimes h_{eq_1}^{i,i+1}\otimes h_{eq_2}^{i,i+1}$, where $h_{eq_1}^{i,i+1}$ and $h_{eq_2}^{i,i+1}$ are identical except they act on different parts of the local Hilbert space.

We will then "integrate out" eq_2 in the next stage of the renormalisation procedure leaving us with only a single set (as per Section E). Thus for the purposes of the RG procedure, we need only consider how the coupling terms transform for a particular $(i, i+1; j)$ set (as we will integrate out the other set anyway).

We now consider the terms coupling the classical and quantum parts of the Hilbert space. Consider term F.1d. In any 2×2 block in the restricted subspace, at most one free $|L\rangle$ or $|R\rangle$ may appear (i.e. not parity cross), and under the classical renormalisation mapping, we see that a 2×2 block with a free cross is mapped to a cross supertile of the same colour and with relevant orientation. Any parity cross is removed in the renormalisation step, as per Subsection C.2. Then we realise that the 2×2 block only receives the penalty iff $|L\rangle$ is not combined with $|\circledless\rangle$. Since under the RG operations $|L\rangle \to |R(L)\rangle$, and $|\circledless\rangle |x\rangle \to |\circledless x\rangle$ (where $|x\rangle \in \mathfrak{B}$ are single site states

of the original Hamiltonian) we see that the new term must penalise states which do not satisfy these states being paired. The parity $|L\rangle$ tiles will be integrated out, however, these are associated with history states that will be integrated out in the same step, and hence can be ignored. Thus term F.1d becomes:

$$|R(L)\rangle\langle R(L)|^{(i)} \otimes \left(\mathbb{1}_{eq'} - |\circledless x\rangle\langle \circledless x| - |e\circledless\rangle\langle e\circledless|\right) \otimes \mathbb{1}^{(i+1)}_{ceq'}.$$

By similar reasoning, after k applications of the RG mapping, we get:

$$\left|R^{(k)}(L)\right\rangle\left\langle R^{(k)}(L)\right|^{(i)} \otimes \left(\mathbb{1}_{eq'} - \sum_m \sum_{x_t \in \mathfrak{B}} \left|e^{\times m}\circledless\{x_t\}^{\times 2^k - m - 1}\right\rangle\left\langle e^{\times m}\circledless\{x_t\}^{\times 2^k - m - 1}\right|\right) \otimes \mathbb{1}^{(i+1)}_{ceq'}.$$

The term F.1f transforms analogously.

Now consider term F.1e. Again, 2×2 blocks in the restricted subspace with the free tile being $|L\rangle$ get renormalised to $|R(L)\rangle$. We see that this term penalises anything but $|\circledless\rangle$ being combined with it, and hence we see it is mapped to:

$$(\mathbb{1}_c - |R(L)\rangle\langle R(L)|_c)^{(i)} \otimes \left(|\circledless x\rangle\langle \circledless x|^{(i)} + |e\circledless\rangle\langle e\circledless|^{(i)}\right) \otimes \mathbb{1}^{(i+1)}_{ceq}.$$

By similar reasoning, after k iterations we get:

$$(\mathbb{1}_c - \left|R^{(k)}(L)\right\rangle\left\langle R^{(k)}(L)\right|_c)^{(i)} \otimes \left(\sum_m \sum_{x_t \in \mathfrak{B}} \left|e^{\times m}\circledless\{x_t\}^{\times 2^k - m}\right\rangle\left\langle e^{\times m}\circledless\{x_t\}^{\times 2^k - m}\right|\right)^{(i)} \otimes \mathbb{1}^{(i+1)}_{ceq}.$$

The F.1g transforms analogously.

We now consider term F.1j. If we consider the term acting between 2×2 blocks, then this is only violated if there is a $|L\rangle_c$ at site (i, j) and at the neighbouring site $(i+1, j)$ is in state $|e\rangle_e$. The renormalised basis states which get penalised by this are then:

$$|R(L)\rangle\langle R(L)|^{(i)}_c \otimes \mathbb{1}^{(i)}_{eq} \otimes \mathbb{1}^{(i+1)}_c \otimes \left(|ee\rangle\langle ee|_e + \sum_{|x\rangle \in \mathfrak{B}} |ex\rangle\langle ex|_{q'}\right)^{(i+1)}.$$

After k iterations this becomes:

$$\left|R^{(k)}(L)\right\rangle\!\left\langle R^{(k)}(L)\right|_c^{(i)} \otimes \mathbb{1}_{eq}^{(i)} \otimes \mathbb{1}_c^{(i+1)} \otimes \left(\sum_m \sum_{x_t\in\mathfrak{B}} \left|e^{\times m}\{x\}^{\times 2^k-m}\right\rangle\!\left\langle e^{\times m}\{x\}^{\times 2^k-m}\right|\right)^{(i+1)}.$$

Term F.1i transforms analogously.

We now consider term F.1k. This term forces a non-$|e\rangle_e$ to the left of any other non-blank in the q-layer, except when a non-blank coincides with an $|L\rangle$ in the c-layer. Again, we see that this penalty term is zero within any 2×2 blocks in the restricted subspace $\kappa_{i,j}$, so we need only consider the interactions between such states. If there is a $|e\rangle_e$ state next to a $|x\rangle$ state in the blocks, then we see that the quantum part of this tile must get mapped to $|e\rangle_e$ or $|R(x)\rangle$. The new term in the Hamiltonian becomes:

$$\mathbb{1}_c^{(i)} \otimes \left(|ee\rangle\langle ee|_e + \sum_{|y\rangle\in\mathfrak{B}} |ye\rangle\langle ye|_q\right)^{(i)} \otimes (\mathbb{1}_c - |R(L)\rangle\langle R(L)|)_c^{(i+1)} \otimes (\mathbb{1}_{eq} - |ee\rangle\langle ee|)_e^{(i+1)}.$$

After k iterations of the RG map the term becomes:

$$\mathbb{1}_c^{(i)} \otimes \left(\sum_{m=1}\sum_{x_t\in\mathfrak{B}} \left|\{x_t\}^{\times 2^k-m}, e^{\times m}\right\rangle\!\left\langle\{x_t\}^{\times 2^k-m}, e^{\times m}\right|\right)^{(i)} \otimes (\mathbb{1}_c - |R(L)\rangle\langle R(L)|)_c^{(i+1)}$$
$$\otimes\left(\mathbb{1}_{eq} - \sum_{m=1}\sum_{x_t\in\mathfrak{B}} \left|e^{\times m}, \circledless, \{x_t\}^{\times 2^k-m-1}\right\rangle\!\left\langle e^{\times m}, \circledless, \{x_t\}^{\times 2^k-m-1}\right|\right)_e^{(i+1)}.$$

Term F.1l transforms analogously.

Identity Terms

Finally we need to consider how terms of the form $\mathbb{1}_{ceq}^{(i)}$ and $\mathbb{1}_{ceq}^{(i)} \otimes \mathbb{1}_{ceq}^{(i+1)}$ transform; as per Remark 32 these terms appear as the Hamiltonian is iterated. consider the two local terms $\mathbb{1}^{(i,j)} \otimes \mathbb{1}^{(i+1,j)}$:

$$\left(\mathbb{1}^{(i,j)} \otimes \mathbb{1}^{(i+1,j)} + \mathbb{1}^{(i,j+1)} \otimes \mathbb{1}^{(i+1,j+1)}\right) \to 2\mathbb{1}^{(i/2,j/2)}.$$

Similarly, consider

$$\left(\mathbb{1}^{(i+1,j)} \otimes \mathbb{1}^{(i+2,j)} + \mathbb{1}^{(i+1,j+1)} \otimes \mathbb{1}^{(i+2,j+1)}\right) \to 2\mathbb{1}^{(i/2,j/2)} \otimes \mathbb{1}^{(i/2+1,j/2)}.$$

Consider the $\mathbb{1}^{(i,j)}$ terms, then

$$\mathbb{1}^{(i,j)} + \mathbb{1}^{(i+1,j)} + \mathbb{1}^{(i,j+1)} + \mathbb{1}^{(i+1,j+1)} \to 4\mathbb{1}^{(i/2,j/2)}. \tag{F.2}$$

Combining these terms, we see that these create new 1-local terms which, after k iterations have coefficients:

$$(-4^k + \sum_{m=0}^{k} (4^m \times 2^{m-k}))\mathbb{1}^{(i/2+1,j/2)} = -2^{-k}\mathbb{1}^{(i/2+1,j/2)},$$

and 2-local terms of the form:

$$2^k \mathbb{1}^{(i/2,j/2)} \otimes \mathbb{1}^{(i/2+1,j/2)}.$$

Note that these 2-local terms only occur in the row interactions, and remain zero for the column interactions.

So far we have shown that all terms in the Hamiltonian transform to an analogous term to one in the original Hamiltonian. Now note the fact the Hamiltonian can be block-decomposed into subspaces with respect to states containing ⧀ and ⧁, and into a classical and quantum part. Then realise that the local quantum Hilbert space can be decomposed as $R^{(k)}(\mathcal{H}_e) \oplus R^{(k)}(\mathcal{H}_q)$. These properties allow the proof from Lemma 51 of [1] to be applied (we refer the reader to this proof for brevity) which also shows that states which are not $R^{(k)}(H_q)$ eigenstates between $|R^{(k)}(L)\rangle$ and $|R^{(k)}(R)\rangle$ markers have energy at least 1.

□

With this, we now wish to prove claim 3 of Lemma 33 and hence need to find the ground state energy for the renormalised Hamiltonian. To do so we need the concept of *tiling defects*:

Definition 45 (Tiling Defect)**.** *A pair $|t_a\rangle_{i,j}, |t_b\rangle_{i+1,j} \in \mathcal{H}_c$ form a tiling defect if they violate the local term between them: $\langle t_a| \langle t_b| h_c^{i,i+1} |t_a\rangle |t_b\rangle = 1$. Similarly, $|t_a\rangle_{i,j}, |t_b\rangle_{i+1,j} \in R^{(k)}(\mathcal{H}_c)$ form a tiling defect if they violate the*

renormalised local term between them: $\langle t_a| \langle t_b| R^{(k)}(h_c)^{(i,i+1)} |t_a\rangle |t_b\rangle = 1$.

In the following lemma we show the ground state is a state with no tiling defects, and as a result the only energy contribution comes from ground states of the Gottesman-Irani Hamiltonians.

Lemma 46 (Restatement of Claim 3 in Lemma 33)**.**
Let $h_c^{\text{row}}, h_c^{\text{col}} \in \mathcal{B}(\mathbb{C}^C \otimes \mathbb{C}^C)$ be the local interactions of the tiling Hamiltonian associated with the modified Robinson tiles, let $R^{(k)}(h_c^{row})^{i,i+1}, R^{(k)}(h_c^{col})^{j,j+1}$ be the local interactions after k RG iterations, and let $h^{\text{row}}, h^{\text{col}} \in \mathcal{B}(\mathbb{C}^{C+Q+1} \otimes \mathbb{C}^{C+Q+1})$ be the local interactions defined in Lemma 44. For a given ground state configuration (tiling) of $R^{(k)}(H_c)$, let $\mathcal{L}$ denote the set of all horizontal line segments of the lattice that lie between down/right-facing and down/left-facing red crosses (inclusive) in the Robinson tiling after k RG mappings.

Then the renormalised Hamiltonian on a 2D square lattice of width L and height H with nearest-neighbour interactions $R^{(k)}(h^{\text{row}}), R^{(k)}(h^{\text{col}})$ has a ground state energy $\lambda_0(R^{(k)}(H)^{\Lambda(L\times H)})$ contained in the interval

$$\Bigg[(g(k) - 4^k\alpha_2(\varphi))LH - 2^{-k}H + \sum_{n=1}^{\lfloor \log_4(L/2)\rfloor} \left(\left\lfloor \frac{H}{2^{2n+1(k\ mod 2)}} \right\rfloor \times \left(\left\lfloor \frac{L}{2^{2n+1-(k\ mod 2)}} \right\rfloor - 1 \right) \right) \lambda_0(R^{(k)}(H_q)(4^{n-\lfloor (k\ mod 2)/2 \rfloor})),$$
$$(g(k) - 4^k\alpha_2(\varphi))LH - 2^{-k}H + \sum_{n=1}^{\lfloor \log_4(L/2)\rfloor} \left(\left(\left\lfloor \frac{H}{2^{2n+1-(k\ mod 2)}} \right\rfloor + 1 \right) \times \left\lfloor \frac{L}{2^{2n+1-(k\ mod 2)}} \right\rfloor \right) \lambda_0(R^{(k)}(H_q)(4^{n-\lfloor (k\ mod 2)/2 \rfloor}))\Bigg]$$

where

$$g(k) = 4^k \sum_{4^n+1<2^k} 4^{-2n-1} \lambda_0(H_q(4^n)). \tag{F.3}$$

Proof. We identify the red down-left and down-right cross tiles from the k-times renormalised tile set with the $|R^{(k)}(L)\rangle$ and $|R^{(k)}(R)\rangle$ state respectively. For convenience, assume $k \in 2\mathbb{N}$ (we will deal with the other case separately $k \in 2\mathbb{N}+1$). From Lemma 44 the ground state of the Hamiltonian is a product state $|T\rangle_c \otimes |\psi_0\rangle_{eq}$ has a $\left|e^{\times 2^k}\right\rangle$ state combined with every tile except those between $|R^{(k)}(L)\rangle$ and $|R^{(k)}(R)\rangle$, where instead there is

a ground state of a $R^{(k)}(H_q)$ Hamiltonian between the two markers. For such states, the terms F.1d-F.1l give zero energy contribution and we need only consider the terms F.1a, F.1b, and F.1c. The terms F.1m and F.1n are constant offsets, and so we will ignore them initially and consider them at the end.

We now consider the energy of the tiling + quantum; from lemma 48 of [1] the number of segments is lower bounded by $\geq \lfloor H2^{-2n-1} \rfloor (\lfloor L2^{-2n-1} - 1 \rfloor)$ and upper bounded by $\leq \lfloor H2^{-2n-1} + 1 \rfloor (\lfloor L2^{-2n-1} \rfloor)$.

In the case we have d defects in the tiling, the energy is at least

$$\begin{aligned} E(d \text{ defects}) &= d + LH(g(k) - 4^k \alpha_2(\varphi)) + \sum_{\ell \in \mathcal{L}} \lambda_0(R^{(k)}(H_q)(|\ell|)) \\ &\geq d + LH(g(k) - 4^k \alpha_2(\varphi)) \\ &+ \sum_{n=1}^{\lfloor \log_4(L/2) \rfloor} \left(\left\lfloor \frac{H}{2^{2n+1}} \right\rfloor \left(\left\lfloor \frac{L}{2^{2n+1}} \right\rfloor - 1 \right) - 2d \right) \lambda_0(R^{(k)}(H_q)(4^n)), \end{aligned}$$

where in the second line we have used the result from lemma 49 of [1] to bound the number of segments of size 2^{2n} is at least $\left\lfloor \frac{H}{2^{2n+1}} \right\rfloor \left(\left\lfloor \frac{L}{2^{2n+1}} \right\rfloor - 1 \right) - 2d$. Note, that lemma 49 of [1] still applies to the renormalised Hamiltonian terms as the tiling rules for the renormalised tile set are identical to the original tile set, as per Lemma 12.

It can be shown from definition 50 of [1] that $\sum_{n=1}^{\infty} \lambda_0(H_q(4^n + 1)) < 1/2$, and since each defect carries an energy penalty of at least 1 we see the ground state is always achieved in the case where there are no defects and hence the Robinson tiling is correct. Thus we see that the ground state is given by

$$E = LH(g(k) - 4^k \alpha_2(\varphi)) + \sum_{\ell \in \mathcal{L}} \lambda_0(R^{(k)}(H_q)(|\ell|)).$$

Again we use the bound on the number of segments allowed from lemma 48 of [1] to show that the ground state energy lies in the bounds

$$\sum_{\ell\in\mathcal{L}} \lambda_0(R^{(k)}(H_q)(|\ell|)) \in \left[\sum_{n=1}^{\lfloor \log_4(L/2)\rfloor} \left(\left\lfloor \frac{H}{2^{2n+1}} \right\rfloor \left(\left\lfloor \frac{L}{2^{2n+1}} \right\rfloor - 1 \right) \right) \lambda_0(R^{(k)}(H_q)(4^n)), \right. \tag{F.4}$$

$$\left. \sum_{n=1}^{\lfloor \log_4(L/2)\rfloor} \left(\left(\left\lfloor \frac{H}{2^{2n+1}} \right\rfloor + 1 \right) \left\lfloor \frac{L}{2^{2n+1}} \right\rfloor \right) \lambda_0(R^{(k)}(H_q)(4^n)) \right] \tag{F.5}$$

Finally consider the constant energy offset from the terms F.1m and F.1n. After k iterations of the RG mapping, from the definition of $g(k)$ in eq. (F.3), the coefficient of the $\mathbb{1}^{(i)}$ term is

$$\begin{aligned} b_1 &:= 4^k \sum_{4^n+1<2^k} 4^{-2n-1}\lambda_0(H_q(4^n)) + 4^k(1-\alpha_2(\varphi)) - 4^k \sum_{m=1}^{k} 2^{-m} \\ &= 4^k \sum_{4^n+1<2^k} 4^{-2n-1}\lambda_0(H_q(4^n)) + 4^k(1-\alpha_2(\varphi)) - 4^k(1-2^{-k}), \end{aligned}$$

where the $-4^k\sum_{m=1}^{k} 2^{-m}$ term arises due to part of the 2-local terms being integrated into the 1-local terms. The coefficient in front of the 2-local term $\mathbb{1}^{(i)}\otimes\mathbb{1}^{(i+1)}$ is then $b_2 := -2^k$. The energy contribution from these term is:

$$\begin{aligned} & b_1 LH + b_2(L-1)H = (b_1+b_2)LH - b_2 H \\ &= \left(4^k \sum_{4^n+1<2^k} 4^{-2n-1}\lambda_0(H_q(4^n)) + 4^k(1-\alpha_2(\varphi)) - 4^k(1-2^{-k}) - 2^{-k} \right) LH + b_2 H \\ &= \left(4^k \sum_{4^n+1<2^k} 4^{-2n-1}\lambda_0(H_q(4^n)) - 4^k\alpha_2(\varphi) \right) LH - 2^k H \\ &= (g(k) - 4^k\alpha_2(\varphi))LH - 2^k H, \end{aligned}$$

where $g(k)$ is defined in the lemma statement. Adding this to the energy contribution from the renormalised Gottesman-Irani segments gives the value in the lemma statement.

For $k \in 2\mathbb{N}+1$ all of the above goes through with

$$L/2^{2n+1} \to L/2^{2n+1-(k\ mod 2)},$$
$$H/2^{2n+1} \to H/2^{2n+1-(k\ mod 2)},$$
$$\lambda_0(H_q(4^n)) \to \lambda_0(H_q(4^{n-\lfloor (k\ mod 2)/2 \rfloor})).$$

This accounts for distances being reduced by a factor of two in alternate RG steps.

□

G Fixed points of the RG flow

Theorem 41 shows that our RG scheme satisfies the expected properties. We now qualitatively examine the Hamiltonian for large values of k.

G.1 Fixed Point for Gapped Instances

Here we show that for gapped instances the Hamiltonian becomes "Ising-like", for appropriately small energy scales. From Corollary 38 the renormalised Hamiltonian is

$$R^{(k)}(h^{row}(\varphi))^{(i,j)} = 2^k(|0\rangle\langle 0|^{(i)} \otimes \Pi_{ud}^{(j)} + |0\rangle\langle 0|^{(i)} \otimes \Pi_{ud}^{(j)}) \tag{G.1}$$
$$+R^{(k)}(h_u^{row}(\varphi)')^{(i,j)} \otimes \mathbb{1}_d^{(i,j)} + \mathbb{1}_u^{(i,j)} \otimes R^{(k)}(h_d)^{(i,j)} \tag{G.2}$$
$$+2^k \Pi_{ud}^{(i)} \otimes \Pi_{ud}^{(j)} \tag{G.3}$$
$$R^{(k)}(h^{col}(\varphi))^{(i,j)} = 2^k(|0\rangle\langle 0|^{(i)} \otimes \Pi_{ud}^{(j)} + |0\rangle\langle 0|^{(i)} \otimes \Pi_{ud}^{(j)}) \tag{G.4}$$
$$+R^{(k)}(h_u^{col}(\varphi)')^{(i,j)} \otimes \mathbb{1}_d^{(i,j)} \tag{G.5}$$
$$R^{(k)}(h(\varphi))^{(1)} = (g(k) - 4^k\alpha_2(\varphi) - 2^k)\Pi_{ud} + R^{(k)}(h_u^{(1)}(\varphi)), \tag{G.6}$$

where here we have explicitly separated out $\Pi_{ud}^{(i)} \otimes \Pi_{ud}^{(j)}$ from the term $R^{(k)}(h_u^{row}(\varphi))^{(i,j)} = R^{(k)}(h_u^{row}(\varphi)')^{(i,j)} + \Pi_{ud}^{(i)} \otimes \Pi_{ud}^{(j)}$.

Define the Ising-like Hamiltonian with local terms:

$$h_{Ising}^{\prime row}(k)^{(i,j)} := 2^k \left(|0\rangle\langle 0|^{(i)} \otimes \Pi_{ud}^{(j)} + \Pi_{ud}^{(j)} \otimes |0\rangle\langle 0|^{(i)} + \Pi_{ud}^{(i)} \otimes \Pi_{ud}^{(j)} \right) \tag{G.7}$$
$$h_{Ising}^{\prime col}(k)^{(i,j)} := 2^k \left(|0\rangle\langle 0|^{(i)} \otimes \Pi_{ud}^{(j)} + \Pi_{ud}^{(j)} \otimes |0\rangle\langle 0|^{(i)} \right) \tag{G.8}$$
$$h'_{Ising}(k)^{(1)} := B(k)\Pi_{ud}. \tag{G.9}$$

This is reminiscent of the Ising interaction with both an ferromagnetic $|0\rangle\langle 0|^{(i)} \otimes |1\rangle\langle 1|^{(j)} + |1\rangle\langle 1|^{(i)} |0\rangle\langle 0|^{(j)}$ along the rows and columns and an anti-ferromagnetic $|1\rangle\langle 1|^{(i)} \otimes |1\rangle\langle 1|^{(j)}$ term along just the rows, with local field $B(k) = (g(k) - 4^k\alpha_2(\varphi) - 2^k) |1\rangle\langle 1|$, but with the orthogonal projector Π_{ud} playing the role of the projector onto the $|1\rangle\langle 1|$ state. However, note that Π_{ud} projects onto a larger dimensional subspace than $|1\rangle\langle 1|$, so e.g. the partition function of this Ising-like Hamiltonian is not identical to that of an Ising model.

We now show the following:

Proposition 47. *Let E be a fixed energy cut-off and $H'_{Ising}(k) = \sum_{\langle i,j\rangle} h'_{Ising}(k)^{(i,j)}$. Then*

$$\left\| R^{(k)}(H(\varphi))|_{\leq E} - H'_{Ising}(k)|_{\leq E} \right\|_{op} \leq \left(\frac{E}{2^k}\right)^2 . \tag{G.10}$$

Proof. Consider the local interaction term $h_0 = |0\rangle\langle 0| \otimes \Pi_{ud} + \Pi_{ud} \otimes |0\rangle\langle 0|$. This commutes with all other terms in both the $R^{(k)}(H(\varphi))$ Hamiltonian and the Ising-like Hamiltonian, and hence the eigenstates of both of the overall Hamiltonians are also eigenstates of $|0\rangle\langle 0| \otimes \Pi_{ud} + \Pi_{ud} \otimes |0\rangle\langle 0|$. As a result, for each eigenstate, a given site $p \in \Lambda$ either has support only on $|0\rangle_p$ or only on $R^{(k)}(\mathcal{H}_{ud})$. Therefore, an eigenstate defines regions (domains) of the lattice where all points in the domain are in $\mathcal{H}_{ud}$.

For a given eigenstate $|\psi\rangle$, let $D := \left\{ i \in \mathbb{Z}^2 | \operatorname{tr}\left(|0\rangle \langle 0|^{(i)} |\psi\rangle \langle \psi| \right) = 0 \right\}$ denote the region of the lattice where the state is supported on $R^{(k)}(\mathcal{H}_{ud})$, and ∂D be the set of sites on the boundary of D. Then we see that the terms in eq. (G.2) act non-trivially only within D, and that the boundaries of D receive an energy penalty of $2^k|\partial D|$ from terms in eq. (G.1) and eq. (G.4).

Note that $\left\|R^{(k)}(h_d)^{(i,j)}\right\|_{op}, \left\|R^{(k)}(h_u(\varphi)')^{(i,j)}\right\|_{op}, \left\|R^{(k)}(h_u^{(1)}(\varphi))\right\|_{op} \leq 2$. For $\left\|R^{(k)}(h_d)^{(i,j)}\right\|_{op}$ this is straightforward to see. For $\left\|R^{(k)}(h_u(\varphi)')^{(i,j)}\right\|_{op}$, any states which pick up non-zero energy, other than those which receive a penalty due to halting, are removed from the local Hilbert space (as per Section D).

Let $m \in \mathbb{N}$ be a cut-off such that $|\partial D| \leq m$, hence $|D| \leq m^2/16$. Since for each boundary term we get an energy penalty of at least 2^k from h_0, we can relate m to the energy cut-off E to m as $E := 2^k m$. If we consider the

Hamiltonians restricted to a subspace with energy $\leq E := 2^k m$, then:

$$\left\| R^{(k)}(H(\varphi))|_{\leq E} - H'_{Ising}(k)|_{\leq E} \right\|_{op} \tag{G.11}$$

$$= \left\| \sum_{\langle i,j \rangle} \left(R^{(k)}(h_u(\varphi)')^{(i,j)} \otimes \mathbb{1}_d^{(i,j)} + \mathbb{1}_u^{(i,j)} \otimes R^{(k)}(h_d)^{(i,j)} \right) \Big|_{\leq E} \right\|_{op} \tag{G.12}$$

$$\leq \frac{m^2}{16} \left(\left\| R^{(k)}(h_u(\varphi)')^{(i,j)} \right\|_{op} + \left\| R^{(k)}(h_d)^{(i,j)} \right\|_{op} + \left\| R^{(k)}(h_u^{(1)}(\varphi)) \right\|_{op} \right) \tag{G.13}$$

$$\leq \frac{m^2}{2} < \left(\frac{E}{2^k} \right)^2. \tag{G.14}$$

Going from eq. (G.12) to eq. (G.13) we have used the fact that the terms in the sum are only non-zero within domains, and $|D| \leq m^2/16$. Going from eq. (G.13) to eq. (G.14) we have used the bound on the individual norms of the local terms. □

Thus, for appropriately small energies, we expect only small deviations from the "Ising-like" Hamiltonian. And these deviations vanish as the RG process is iterated.

G.2 Fixed Point for Gapless Instances

For a φ for which $H(\varphi)$ is gapless, $R^{(k)}(H(\varphi))$ is also gapless and we see that the ground state is that of $R^{(k)}(H_u(\varphi))$. If we restrict to a low energy subspace, one can see that excited states are either the excited states of the Gottesman-Irani Hamiltonians or the excited states of the critical XY-model. Indeed, let $E(k)$ be the subspace of states with energy less than 2^k, then for sufficiently large k we see that

$$R^{(k)}(H)^{\Lambda}|_{E(k)} = R^{(k)}(H_u(\varphi))^{\Lambda}|_{E(k)} \otimes \mathbb{1}_d^{\Lambda} + \mathbb{1}^{\Lambda} \otimes R^{(k)}(H_d)^{\Lambda}|_{E(k)}. \tag{G.15}$$

Since $R^{(k)}(H_d)^{\Lambda}|_{E(k)}$ has the same spectrum as H_d, the spectrum of $R^{(k)}(H)^{\Lambda}|_{E(k)}$ is also dense in the thermodynamic limit. Furthermore, $R^{(k)}(H)^{\Lambda}|_{E(k)}$ has algebraically decaying correlations since $R^{(k)}(H_d)^{\Lambda}|_{E(k)}$ also has algebraically decaying correlations [2].

Supplementary References

[1] T. S. Cubitt, D. Pérez-García and M. M. Wolf. "Undecidability of the spectral gap". In: *Forum of Mathematics, Pi* 10 (2022), e14. DOI: 10.1017/fmp.2021.15.

[2] E.H. Lieb, T.H. Schultz and D.C. Mattis. "Two soluble models of an antiferromagnetic chain". In: *Annals of Physics* 15.3 (1961), 472–473. DOI: 10.1016/0003-4916(61)90208-1.

[3] Daniel Gottesman and Sandy Irani. "The quantum and classical complexity of translationally invariant tiling and Hamiltonian problems". In: *Foundations of Computer Science, 2009. FOCS'09. 50th Annual IEEE Symposium on*. IEEE. 2009, pp. 95–104.

[4] Alexei Yu. Kitaev, Alexander Shen and Mikhail N. Vyalyi. "Classical and quantum computing". In: *Quantum Information*. New York, NY: Springer New York, 2002, pp. 203–217. DOI: 10.1007/978-0-387-36944-0_13.

[5] John Cardy. *Scaling and renormalization in statistical physics*. Cambridge University Press, 1996.

[6] G. Vidal. "Class of Quantum Many-Body States That Can Be Efficiently Simulated". In: *Physical Review Letters* 101.11 (2008). DOI: 10.1103/physrevlett.101.110501.

[7] R. Jullien, P. Pfeuty, J. N. Fields and S. Doniach. "Zero-temperature renormalization method for quantum systems. I. Ising model in a transverse field in one dimension". In: *Phys. Rev. B* 18 (7 1978), pp. 3568–3578. DOI: 10.1103/PhysRevB.18.3568.

[8] R. Jullien and P. Pfeuty. "Zero-temperature renormalization-group method for quantum systems. II. Isotropic $X - Y$ model in a transverse field in one dimension". In: *Phys. Rev. B* 19 (9 1979), pp. 4646–4652. DOI: 10.1103/PhysRevB.19.4646.

[9] K. A. Penson, R. Jullien and P. Pfeuty. "Zero-temperature renormalization-group method for quantum systems. V. Frustration in two dimensions". In: *Phys. Rev. B* 25 (3 1982), pp. 1837–1847. DOI: 10.1103/PhysRevB.25.1837.

[10] Bibhas Bhattacharyya and Shreekantha Sil. “The Hubbard model with bond-charge interaction on a triangular lattice: a renormalization group study”. In: *Journal of Physics: Condensed Matter* 11.17 (1999), pp. 3513–3523. DOI: 10.1088/0953-8984/11/17/309.

[11] Miguel A. Martín-Delgado and Germán Sierra. “Real Space Renormalization Group Methods and Quantum Groups”. In: *Phys. Rev. Lett.* 76 (7 1996), pp. 1146–1149. DOI: 10.1103/PhysRevLett.76.1146.

[12] J. Wang, Sabre Kais and R. Levine. “Real-space renormalization group study of the Hubbard model on a non-bipartite lattice”. In: *International Journal of Molecular Sciences* 3.1 (2002), 4–16. DOI: 10.3390/i3010004.

[13] Johannes Bausch, Toby S. Cubitt and James D. Watson. “Uncomputability of phase diagrams”. In: *Nature Communications* 12.1 (2021). DOI: 10.1038/s41467-020-20504-6.

[14] Toby S. Cubitt, David Pérez-García and Michael M. Wolf. “Undecidability of the spectral gap”. In: *Nature* 528.7581 (2015), pp. 207–211. DOI: 10.1038/nature16059. arXiv: 1502.04573.

[15] Raphael M. Robinson. “Undecidability and nonperiodicity for tilings of the plane”. In: *Inventiones mathematicae* 12.3 (1971), pp. 177–209. DOI: 10.1007/BF01418780.

[16] James D. Watson. “Detailed Analysis of Circuit-to-Hamiltonian Mappings”. In: (2019). arXiv: 1502.04573 [quant-ph].
